# Increasing genomic prediction accuracy for unphenotyped full-sib families by modeling additive and dominance effects with large datasets in white spruce

**DOI:** 10.3389/fpls.2023.1137834

**Published:** 2023-03-22

**Authors:** Simon Nadeau, Jean Beaulieu, Salvador A. Gezan, Martin Perron, Jean Bousquet, Patrick R. N. Lenz

**Affiliations:** ^1^ Natural Resources Canada, Canadian Forest Service, Canadian Wood Fibre Centre, Québec, QC, Canada; ^2^ Canada Research Chair in Forest Genomics, Institute for Systems and Integrative Biology, Université Laval, Québec, QC, Canada; ^3^ VSN International Ltd, Hemel Hempstead, United Kingdom; ^4^ Direction de la Recherche Forestière, Ministère des Ressources Naturelles et des Forêts, Québec, QC, Canada

**Keywords:** Genomic selection (GS), non-additive genetic effects, mate allocation, wood quality traits, growth traits, conifers, GBLUP, tree breeding programs

## Abstract

**Introduction:**

Genomic selection is becoming a standard technique in plant breeding and is now being introduced into forest tree breeding. Despite promising results to predict the genetic merit of superior material based on their additive breeding values, many studies and operational programs still neglect non-additive effects and their potential for enhancing genetic gains.

**Methods:**

Using two large comprehensive datasets totaling 4,066 trees from 146 full-sib families of white spruce (Picea glauca (Moench) Voss), we evaluated the effect of the inclusion of dominance on the precision of genetic parameter estimates and on the accuracy of conventional pedigree-based (ABLUP-AD) and genomic-based (GBLUP-AD) models.

**Results:**

While wood quality traits were mostly additively inherited, considerable non-additive effects and lower heritabilities were detected for growth traits. For growth, GBLUP-AD better partitioned the additive and dominance effects into roughly equal variances, while ABLUP-AD strongly overestimated dominance. The predictive abilities of breeding and total genetic value estimates were similar between ABLUP-AD and GBLUP-AD when predicting individuals from the same families as those included in the training dataset. However, GBLUP-AD outperformed ABLUP-AD when predicting for new unphenotyped families that were not represented in the training dataset, with, on average, 22% and 53% higher predictive ability of breeding and genetic values, respectively. Resampling simulations showed that GBLUP-AD required smaller sample sizes than ABLUP-AD to produce precise estimates of genetic variances and accurate predictions of genetic values. Still, regardless of the method used, large training datasets were needed to estimate additive and non-additive genetic variances precisely.

**Discussion:**

This study highlights the different quantitative genetic architectures between growth and wood traits. Furthermore, the usefulness of genomic additive-dominance models for predicting new families should allow practicing mating allocation to maximize the total genetic values for the propagation of elite material.

## 1 Introduction

Since the seminal paper by Meuwissen et al. (2001) genomic selection (GS) has become widely applied in animal and crop breeding (Bhat et al., 2016; Meuwissen et al., 2016). In tree breeding, GS is expected to be highly advantageous due to the long generation times of conventional programs, and the large cost, time, and space required for testing and phenotyping mature traits. During the last decade, different proof of concept studies have successfully tested and applied GS to forest trees (e.g., Resende et al., 2012; Beaulieu et al., 2014a; Isik et al., 2016; Pégard et al., 2020). Publications have underlined the ability of GS to greatly shorten breeding cycles and increase genetic gains generated per time unit (Grattapaglia and Resende, 2011; Denis and Bouvet, 2013; Beaulieu et al., 2014b; Chen et al., 2018). In GS, a population in which individuals are both phenotyped and genotyped is used to train a model, which is then used to predict the genetic merit of young genotyped, but unphenotyped, offspring. Selections from a large number of genotyped candidates should translate into higher selection intensities, while preserving genetic diversity in improved varieties, or allowing for efficient multi-trait selection strategies (Bouvet et al., 2020; Lenz et al., 2020b; Bousquet et al., 2021; Cappa et al., 2022).

Changing environmental conditions and forest product markets are putting pressure on tree breeding programs to rapidly deliver adapted planting stock with superior end-use quality attributes. Hence, accelerating breeding for improved reforestation material is becoming essential for traits related to wood quality (Hassegawa et al., 2020), biotic stress resistance (Beaulieu et al., 2020; Lenz et al., 2020b; Westbrook et al., 2020; Mphahlele et al., 2021; Gamal El-Dien et al., 2022), and resilience to abiotic stress such as drought (Bouvet et al., 2020; Depardieu et al., 2020; Cappa et al., 2022; Laverdière et al., 2022; Soro et al., 2022). GS can hence play an active role in climate change mitigation strategies and provide more flexibility to tree breeders.

To fully harness the power of GS, models need to fit and rely on both additive and non-additive genetic variances for delivering the most optimal selections for the propagation of elite material. Non-additive genetic variance can be partitioned into dominance and epistasis components; that is the interaction of alleles within genetic loci, and the interaction of alleles among different loci, respectively (Falconer and Mackay, 1996). Simulations showed that considering both additive and non-additive variances, especially dominance, can increase the prediction accuracy of genetic values (Denis and Bouvet, 2013; Nishio and Satoh, 2014; de Almeida Filho et al., 2016, 2019; Nazarian and Gezan, 2016). The inclusion of dominance in GS models improved the prediction of complex traits in animals and crops, such as milk production in dairy cattle (Sun et al., 2014; Aliloo et al., 2016), grain production and drought tolerance in maize hybrids (Dias et al., 2018; Ferrão et al., 2020), or yield in sorghum (Hunt et al., 2020). Still, most tree breeding programs focus solely on the estimation of additive variance and breeding values as seeds for reforestation are generally produced in open-pollinated seed orchards (Mullin et al., 2011). This is because only additive effects are transmitted and accumulate over generations, and they generally account for most of the genetic variance of complex traits (Hill et al., 2008). Thus, non-additive effects are often ignored in tree breeding as their estimation requires more complex crossing schemes, experimental designs, and statistical models, which can be prohibitive considering the short-term costs and benefits. Moreover, they are not easily partitioned from their additive counterpart due to their dependency in practical breeding situations (Muñoz et al., 2014; de Almeida Filho et al., 2019). However, ignoring non-additive effects will result in inflated estimates of additive genetic variance, and lead to biased predictions of breeding values and genetic gains (Araújo et al., 2012; Muñoz et al., 2014). Thus, it is crucial to estimate both additive and non-additive effects to guide the establishment of optimal testing, breeding, selection, and deployment strategies (White et al., 2007; Chen et al., 2020). For deployment, it may be advantageous to exploit dominance by the propagation of elite full-sib families or to utilize both dominance and epistasis when clonal propagation methods are available, for example through rooted cuttings or somatic embryogenesis (Park et al., 2016; Wu, 2019), thus preserving existing allelic combinations within and among loci.

In forest genetics, several studies have attempted to disentangle additive and non-additive effects. Dominance may be separated from additive effects with structured schemes of full-sib crosses, such as in diallel experiments, where each parent is mated with several others. In addition, the partition of additive, dominance, and epistasis components requires clonal repetition of individual genotypes from full-sibs (Foster and Shaw, 1988; Wu, 2019). In tree species, only a few reliable estimates of both dominance and epistatic genetic variances were reported using pedigree-based methods and clonal trials. While some studies reported small non-additive effects (Costa e Silva et al., 2004, 2009; Baltunis et al., 2007), others reported considerable non-additive effects that were of similar magnitude as the additive effects (Mullin and Park, 1992; Baltunis et al., 2009; Araújo et al., 2012; Berlin et al., 2019; Chen et al., 2020). Typically, clonal deployment based on clonal mean selection yielded the largest genetic gains, followed by clonal deployment of elite families, and by seedling deployment from open-pollinated seed orchards (Weng et al., 2008; Baltunis et al., 2009; Wu, 2019; Nguyen et al., 2022). Authors hence concluded that exploiting non-additive variance should be considered in future deployment strategies (Araújo et al., 2012; Park et al., 2016; Li and Dungey, 2018; Berlin et al., 2019).

During the last decade, with the development of high-throughput genotyping methods leading to abundant genetic marker information, quantitative methods have been developed to estimate additive, dominant, and epistasic relationship matrices based on genetic markers (VanRaden, 2008; Su et al., 2012; Vitezica et al., 2013, 2017). While the conventional pedigree-based animal model, often called “ABLUP”, can only describe the expected relationships between individuals (e.g., 0.5 for full-sibs), the genomic additive relationship matrix (***G***) used in GBLUP can estimate the realized relationships based on the fraction of the genome shared between individuals. Furthermore, the ***G***-matrix allows the detection of inbreeding, hidden co-ancestry, and unknown parentage in breeding populations with shallow pedigree, such as for forest trees (Doerksen et al., 2014; Munoz et al., 2014; Lenz et al., 2017, 2020a; Gamal El-Dien et al., 2022).

An increasing number of empirical GS studies in forest trees considered non-additive effects and aimed at their separation from additive effects (Muñoz et al., 2014; Bouvet et al., 2016; de Almeida Filho et al., 2016, 2019; Gamal El-Dien et al., 2016, 2018, 2022; Resende et al., 2017; Tan et al., 2018; Chen et al., 2019; Beaulieu et al., 2020; Pégard et al., 2020; Thavamanikumar et al., 2020; Calleja-Rodriguez et al., 2021; Thumma et al., 2022). Compared with ABLUP, GBLUP was shown to better separate both types of variance and reduce confounding between genetic and environmental effects (Muñoz et al., 2014; Gamal El-Dien et al., 2016; Tan et al., 2018). Nevertheless, even under GBLUP, some level of confounding occurs, and estimates of non-additive effects sometimes carry high standard errors (Gamal El-Dien et al., 2016, 2018; Tan et al., 2018; Chen et al., 2019). In most cases, when significant dominance variance was detected, the additive-dominance model did not result in significant improvements in the prediction ability or accuracy of total genetic values compared with the additive model (Muñoz et al., 2014; Bouvet et al., 2016; de Almeida Filho et al., 2016; Resende et al., 2017; Tan et al., 2018; Beaulieu et al., 2020; Calleja-Rodriguez et al., 2021). Given that the vast majority of GS studies in full-sib populations relied on fewer than 1,000 trees, often from a relatively small number of parents and families (Lebedev et al., 2020), these observations underscore the need for larger datasets with good overlapping crossing schemes and connectivity among families, large numbers of parents, families, and genets, as well as high-quality genotyping data to successfully partition and predict additive and non-additive effects.

One advantage of GBLUP over ABLUP is its ability to predict genetic values within full-sib families of young unphenotyped material by modeling the random Mendelian sampling of alleles (VanRaden, 2008; Legarra et al., 2009). In terms of breeding for the next generation, a realistic approach for GS would be to predict the genetic values of new unphenotyped full-sib families not represented in the training population. Another potentially overlooked application of GS in tree breeding is mating allocation, which also involves predicting the genetic values of future parental crosses (Toro and Varona, 2010). However, the genomic predictions for new families suffer from a large decrease in accuracy due to reduced relatedness between training and validation populations (e.g., Beaulieu et al., 2014b; Lenz et al., 2017; Chen et al., 2018), thus limiting the use of GS in this context (Pégard et al., 2020). In forest trees, most previous studies did not model non-additive effects when predicting new full-sib families (Beaulieu et al., 2014b; Lenz et al., 2017; Chen et al., 2018), or, if they did, they still only estimated the prediction accuracy of breeding values instead of using the total genetic values (Pégard et al., 2020; Shalizi et al., 2021; Lauer et al., 2022; Walker et al., 2022). Thus, more research is needed to determine whether the dominance deviations of offspring in new controlled crosses can be successfully predicted.

In white spruce (*Picea glauca* [Moench] Voss), a widespread Canadian conifer being the subject of major reforestation and breeding efforts (Mullin et al., 2011), very few studies have estimated non-additive effects, with mixed results obtained. Weng et al. (2008) used a large clonal trial and pedigree-based methods to conclude that 81% of the genetic variance for growth was additive, with the rest mostly explained by dominance, and not by epistasis. In contrast, Gamal El-Dien et al. (2016) found that epistatic variances were larger than their additive counterparts for growth and wood traits in an open-pollinated trial, but the standard errors of estimates were large. More recently, Beaulieu et al. (2020) found significant dominance variance for growth and acoustic velocity using GBLUP in a full-sib trial. However, in both Beaulieu et al. (2020) and Gamal El-Dien et al. (2016), modeling non-additive effects did not increase the accuracy of breeding or total genetic values. Finally, Lenz et al. (2020a) did not detect significant dominance variance for both growth and wood traits in a polycross trial. Hence, more studies are required to elucidate whether common traits carry significant proportions of non-additive genetic variance.

The present study uses two uniquely large datasets of 2,458 and 1,608 white spruce trees from 90 and 56 full-sib families, respectively, to (1) evaluate the ability of GS-based GBLUP and conventional pedigree-based ABLUP models to partition additive and dominance variances for growth and wood traits; (2) compare the predictive ability of ABLUP and GBLUP when predicting for the same families as those included in the training population, or for new unphenotyped full-sib families that were not part of the training population; and (3) investigate the effect of sample size on the precision of genetic parameter estimates and the accuracy of genetic values.

## 2 Materials and methods

### 2.1 Genetic material and phenotyping

**Figure 1:**
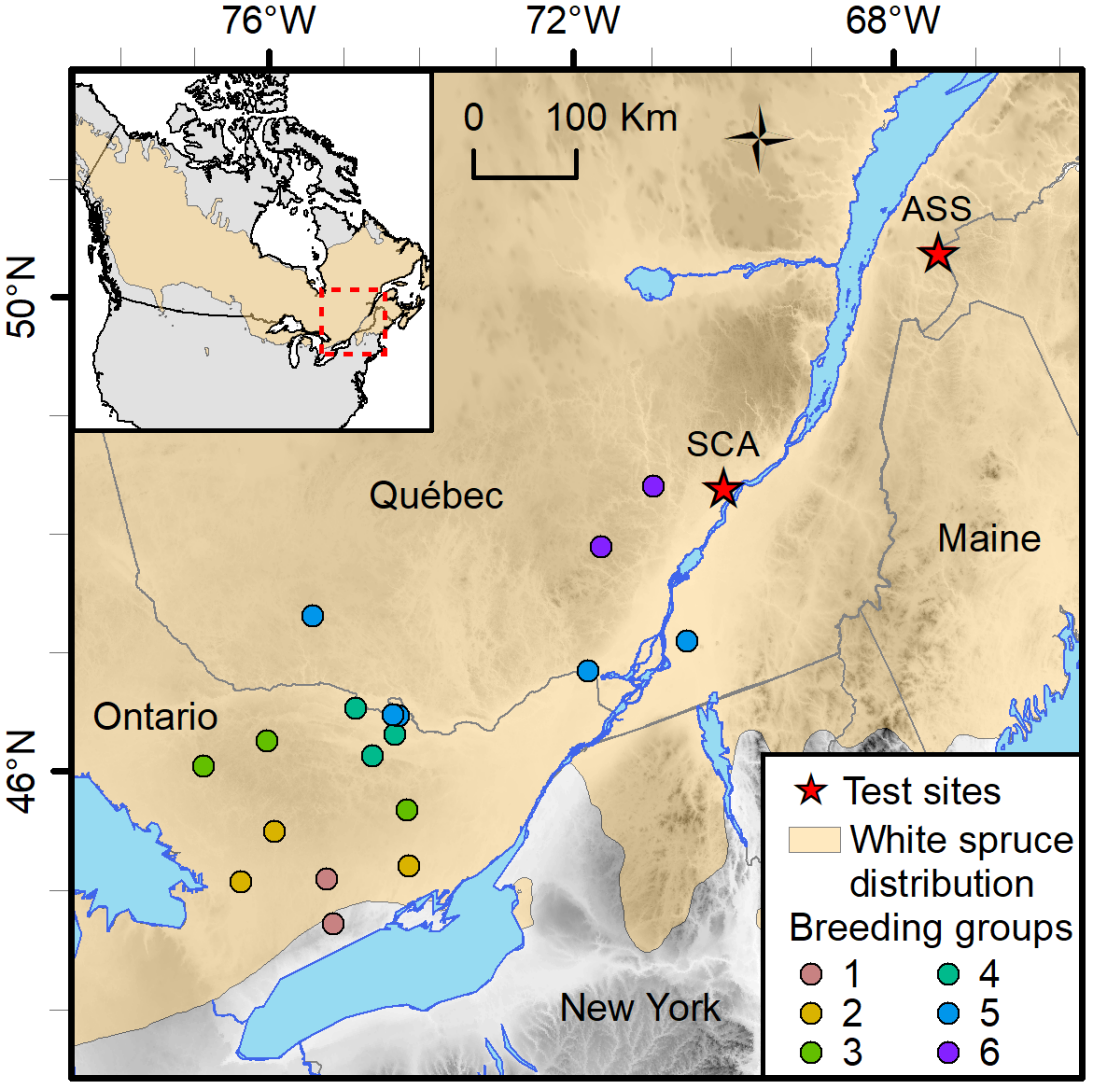
Location of the St. Casimir (SCA) and Asselin (ASS) white spruce test sites in the province of Québec, Canada. The provenances of the genetic material tested in both sites are colored by breeding groups. Each breeding group is composed of two to five provenances.

The material was sampled from a test series designed to assess the genetic merit of first-generation selections of the white spruce breeding program in Québec, Canada, which had been subdivided into six breeding groups (BGs) delineated by their geographic region (**Figure 1**). Crosses were made using a partial diallel mating design within each of the six BGs to limit future inbreeding within groups and to control inbreeding buildup in the production population. Each parent was crossed 1–6 times, giving rise to a mixture of full- and half-sib families within BGs, with no relatedness between BGs (**Figure S1**). The genetic trial was established on two sites in 1999 with 2-year-old nursery-grown seedlings. These sites are Asselin Township (ASS, located in the balsam fir–yellow birch ecological zone, thus cooler climate; Lat. 47° 55’ N, Long. 68° 26’W, Elev. 278 m), and St. Casimir (SCA, located in the maple–basswood ecological zone, thus milder climate; Lat. 46° 42’ N, Long. 72° 06’W, Elev. 52 m; **Figure 1**). The experiment layout was a randomized complete block design with 10 replications. Trees were assigned to row-plots of five trees per plot (2 m × 2 m spacing).

For the present study, 4,245 trees were sampled in the six BGs from 151 full-sib families involving 101 parents. The sampled trees were roughly equally distributed among the two test sites (ASS: 2,061 trees; SCA: 2,184 trees). The phenotypic traits tree height (HT), diameter at breast height (DBH), average wood density (WD), and acoustic velocity (AV) were assessed at age 16 since plantation for BGs 1, 2, 5, and 6, and at age 13 since plantation for BGs 3 and 4. The total volume (VOL, dm^3^), excluding the bark, was calculated following Prégent et al. (2010) as:


(1) $$VOL=0.0344(DBH^{1.8329})(HT^{1.1793})$$

with HT in m and DBH in cm. Average wood density was determined with X-ray densitometry as previously described (Beaulieu et al., 2014b). Acoustic velocity, which is a proxy for wood stiffness measured at standing trees (Lenz et al., 2013), was measured with the Hitman ST300 tool (Fibre-gen, New Zealand).

### 2.2 DNA extraction and SNP genotyping

DNA for the 4,245 trees was isolated from needles and twig buds with the Qiagen DNeasy Plant Kit and quantified with PicoGreen fluorescent dye (Invitrogen). Trees were genotyped using an Infinium iSelect SNP array (Illumina, San Diego, CA) as described in Lenz et al. (2020a). After applying several filters for retaining high-quality data, imputing only a small proportion of genotypes (0.9%), and correcting the registered pedigree using marker data (see Suppl. Methods), a total 4,066 trees from 146 families, genotyped on 4,092 SNPs, were retained for quantitative genetic analyses. The retained SNPs had an average call rate of 99.1%, an average genotyping reproducibility rate of 99.99% as assessed by replicated control genotypes, an average minor allele frequency MAF = 0.211, and an average fixation index *F*_e_ = 0.022.

Genetic diversity within each BG was estimated with the status effective number (*Ns*):


(2) Ns=1/2θ

where θ is the group coancestry (Lindgren et al., 1996), as estimated from the corrected full-sib pedigree. Descriptive statistics of BGs and phenotypes are presented in **Tables 1**, **2**, respectively. Boxplots of phenotypes grouped by sites and BGs are shown in **Figure S2**.

**Table 1:** Number of white spruce trees, full-sib families, and parents sampled for each breeding group (BG) and summarized for all the 4,066 individuals retained for analyses, after pedigree correction using marker information.

	Age of measurements	Number of trees	*Ns*	Number of full-sib families	Number of trees per family			Number of parents	Number of crosses per parent		
					Min.	Max.	Mean		Min	Max.	Mean
Dataset 1	16	2,458	53.4	90	4	56	27.3	62	1	5	2.9
BG 1	16	495	14.7	19	4	50	26.1	17	1	4	2.2
BG 2	16	784	16.6	31	9	34	25.3	19	1	5	3.4
BG 5	16	677	14.1	23	15	56	29.4	16	1	4	2.9
BG 6	16	502	9.4	17	18	43	29.5	10	2	5	3.4
Dataset 2	13	1,608	34.0	56	17	54	28.7	39	1	6	3.1
BG 3	13	713	15.7	24	19	34	29.7	18	1	6	2.9
BG 4	13	895	18.4	32	17	54	28.0	21	1	5	3.2

Also reported are the age of measurements, the status number (*Ns*), the number of trees per family, and the number of crosses per parent. The data was subdivided into dataset 1 (BGs 1, 2, 5 and 6) and dataset 2 (BGs 3 and 4).

**Table 2:** Number of missing values (NA’s), phenotypic mean, standard deviation (SD), and coefficient of variation (CV) for sites Asselin (ASS) and St. Casimir (SCA) using the 4,066 white spruce trees retained for analyses.

Trait^1^	ASS				SCA			
	NA’s	Mean	SD	CV (%)	NA’s	Mean	SD	CV (%)
Dataset 1: (*n*=2,458 trees)^2^								
AV_16_ (km/s)	49	2.8	0.4	14.9	15	3.4	0.4	10.7
WD_16_ (kg/m³)	36	362.4	31.0	8.5	27	371.6	31.3	8.4
HT_16_ (cm)	1	718.0	97.4	13.6	4	818.2	107.7	13.2
DBH_16_ (mm)	1	110.5	19.9	18.0	4	116.2	20.1	17.3
VOL_16_ (dm³)	1	30.6	13.4	43.7	4	39.1	16.8	43.0
Dataset 2: (*n*=1,608 trees)^2^								
AV_13_ (km/s)	57	2.7	0.4	13.9	48	2.9	0.3	11.9
WD_13_ (kg/m³)	102	380.6	29.6	7.8	30	394.4	28.5	7.2
HT_13_ (cm)	8	491.9	77.0	15.6	3	565.7	99.7	17.6
DBH_13_ (mm)	8	74.9	16.0	21.3	3	75.7	16.8	22.2
VOL_13_ (dm³)	8	9.9	5.1	51.6	3	12.1	6.8	56.4

^1^ Measured traits in descending order are acoustic velocity (AV) as a proxy for wood stiffness, average wood density (WD), tree height (HT), diameter at breast height (DBH), and volume (VOL). ^2^ The data was subdivided into two subsets: dataset 1 included breeding groups (BGs) 1, 2, 5, and 6 with phenotypes measured at age 16 since plantation and dataset 2 included BGs 3 and 4 with phenotypes measured at age 13.

### 2.3 Quantitative genetic analyses

For analysis, we subdivided the data into two subsets: 1) the phenotypes measured at age 16 in BGs 1, 2, 5, and 6, hereafter referred to as “dataset 1” (*n*=2,458 trees), and 2) the phenotypes measured at age 13 in BGs 3 and 4, hereafter referred to as “dataset 2” (*n*=1,608 trees). These two datasets were analyzed separately because the measurements were taken at different ages, thus having different means and variances, and also because there was no genetic relatedness between these two datasets (no parents in common). All analyses were conducted in the R v.4.0.2 environment (R Core Team, 2020). The R code is provided in **Supplementary Material**.

For each dataset, we ran individual-tree linear mixed models using pedigree-based relationship matrices among trees (***A, D***), referred to as “ABLUP” models, or using realized genomic relationship matrices (***G_a_, G_d_***), referred to as “GBLUP” models. We ran two additive models (ABLUP-A and GBLUP-A) and two additive-dominance models (ABLUP-AD and GBLUP-AD). The full additive-dominance models were fitted using ASReml-R v.4.1 (Butler et al., 2017) based on the following expression:


(3) $$y=X\beta +Z_{1}b(s)+Z_{2}p(s)+Z_{3}bg+Z_{4}s:bg+Z_{5}a(s)+Z_{6}d(s)+e$$

where ***y*** is the phenotype; ***β*** is a vector of fixed effects including the overall mean and the site effect; ***b*** (***s***) is the random block within site effect; ***p*** (***s***) is the random plot within site effect; and ***e*** is the residual term. The terms ***b*** (***s***), ***p*** (***s***), and ***e*** were fitted with heterogeneous (block diagonal) variance among sites, as $b\left(s\right)∼N\left(0,{⊕}_{i=1}^{2}\sigma_{bi}^{2}\right)$ , p(s)∼N(0,⊕i=12σpi2) and e∼N(0,⊕i=12σei2) , respectively. The term ***bg*** is the random breeding group (BG) effect, with $bg∼N\left(0,\sigma_{bg}^{2}I\right)$ ; ***s*:*bg*** is the random effect of site-by-BG interaction, with $s:bg∼N\left(0,\sigma_{s:bg}^{2}I\right)$ ; ***a*** (***s***) is the random additive genetic effect nested within site, using the pedigree-based relationship matrix ***A*** for ABLUP, with ***a*(*s*)**~ ***N*** **(0,*V_a_*** ⊗ ***A*)**, and using the additive genomic relationship matrix ***G_a_*** for GBLUP, with ***a*(*s*)**~ ***N*(0,*V_a_*** ⊗ ***G_a_*)** ; ***d*** (***s***) is the random dominance genetic effect nested within site, using the pedigree-based dominant relationship matrix ***D*** for ABLUP, with ***d*(*s*)**~ ***N*** **(0,*V***_***d***_⊗ ***D***), and using the dominant genomic relationship matrix ***G_d_*** for GBLUP, with ***d*(*s*)**~*N* **(0,*V***_*d*_⊗ ***G_d_***). The term ***d*(*s*)** was not included for the additive ABLUP-A and GBLUP-A models. The matrices ***X*** and ***Z*_*x*_**  are incidence matrices of their corresponding effects. The matrices ***I*** are identity matrices of their appropriate size. The symbols ⊕ and ⊗ refer to the direct sum and Kronecker product of matrices, respectively.

The matrix ***V*_*a*_** is a 2 x 2 variance-covariance matrix defined by the correlation of additive effects between sites (*r*_*Ba*_) and unique additive variances for site ASS ( $\sigma_{a\_ASS}^{2}$ ) and site SCA ( $\sigma_{a\_SCA}^{2}$ ; i.e., CORH variance structure in ASReml):


(4) $$V_{a}=\;\left[\begin{array}{cc} \sigma_{a\_ASS}^{2} & r_{\mathrm{Ba}}\;\sigma_{a\_ASS}\;\sigma_{a\_SCA}\\ r_{\mathrm{Ba}\;}\sigma_{a\_ASS}\;\sigma_{a\_SCA} & \sigma_{a\_SCA}^{2} \end{array}\right]$$

Similarly, the matrix ***V*_*d*_** was defined by the correlation of dominance effects between sites (*r*_*Bd*_) and unique dominance variances for site ASS ( $\sigma_{d\_ASS}^{2}$ ) and site SCA ( $\sigma_{d\_SCA}^{2}$ ):


(5) $$V_{d}=\;\left[\begin{array}{cc} \sigma_{d\_ASS}^{2} & r_{\mathrm{Bd}}\;\sigma_{d\_ASS}\;\sigma_{d\_SCA}\\ r_{\mathrm{Bd}\;}\sigma_{d\_ASS}\;\sigma_{d\_SCA} & \sigma_{d\_SCA}^{2} \end{array}\right]$$

These heterogeneous additive and dominance genetic variance structures accounted for the fact that the two sites are in different breeding zones of the white spruce breeding program in Québec with contrasting climates (Li et al., 1997).

The additive relationship matrix (***A***) and its inverse were computed from the corrected pedigree using the “Amatrix” and the “ainverse” functions of the R packages AGHmatrix (Amadeu et al., 2016) and ASReml-R v.4.1, respectively. The realized additive genomic relationship matrix (***G_a_***; **Figure S3**) was computed from the marker data following VanRaden (2008) using the “Gmatrix” function of the R package AGHmatrix. To make the matrix ***G_a_*** invertible, it was blended with the matrix ***A*** in the following proportions:


(6) $$G_{a\_blended}=0.98*G_{a}+0.02*A$$

The inverse of the matrix ***G_a_blended_*** was calculated using the “solve” function in the R base package.

The dominant relationship matrix ***D*** and its inverse were computed from the corrected pedigree using the “Amatrix” and the “makeD” functions of the R packages AGHmatrix and nadiv (Wolak 2012), respectively. The The realized dominant genomic relationship matrix ***G_d_*** was computed using the “Gmatrix” function (AGHmatrix) following Vitezica et al. (2013; **Figure S4**) and was blended with the ***D*** matrix using the same proportions as in Eq. [6] before computing its inverse (“solve” function). Blending ***G_a_*** with ***A***, or ***G_d_*** with ***D***, using different proportions of ***G_x_*** (0.95, 0.995) did not change the genetic parameter estimates (results not shown).

Variance components estimated using the full GBLUP-AD models (Eq. [3]) are presented in **Table S1** (dataset 1) and **S2** (dataset 2). Because the effects of ***bg*** and ***s:bg*** were small with large standard errors, we removed these terms from the final models. The final additive-dominance models were then expressed as:


(7) $$y\;=\;X\beta \;+\;Z_{1}b\left(s\right)\;+\;Z_{2}p\left(s\right)\;+\;Z_{3}a\left(s\right)\;+\;Z_{4}d\left(s\right)\;+\;e$$

where the terms are defined in Eq. [3]. The final models (Eq. [7]) had a similar AIC compared to the full models (Eq. [3]; ΔAIC ± 4), but the BIC was always smaller for the final models (ΔBIC: -7 to -15), indicating that the final models were the most parsimonious for all traits (**Tables S1**, **S2**). Similar to this study, Beaulieu et al. (2014b) did not find significant differences in phenotypic trait averages between BGs 3 and 4. Results are also consistent with the relatively weak, though significant, genetic differentiation found among widespread white spruce populations from eastern Canada for quantitative traits (Li et al., 1997; Jaramillo-Correa et al., 2001; Depardieu et al., 2020).

The equations used to obtain genetic parameter estimates within each site from the final additive and additive-dominance models are presented in **Table S3**. For the additive models, across-site estimates of individual narrow-sense heritability were calculated as:


(8) $${\hat{h}}_{ind}^{2}={\hat{r}}_{\mathrm{Ba}}\bar{{\hat{\sigma }}_{a}^{2}}/\left(\bar{{\hat{\sigma }}_{p}^{2}}+\bar{{\hat{\sigma }}_{a}^{2}}+\bar{{\hat{\sigma }}_{e}^{2}}\right)$$

where $\bar{{\hat{\sigma }}_{a}^{2}}$ , $\bar{{\hat{\sigma }}_{p}^{2}}$ , and $\bar{{\hat{\sigma }}_{e}^{2}}$ are the average additive, plot, and residual error variances of the two sites, and ${\hat{r}}_{\mathrm{Ba}}$ is the correlation of additive effects between sites.

For the additive-dominance models, across-site estimates of individual narrow-sense heritability ( ${\hat{h}}_{ind}^{2}$ ), broad-sense heritability ( ${\hat{H}}_{ind}^{2}$ ), and of the portion of individual phenotypic variation due to dominance (the dominance ratio, ${\hat{d}}_{ind}^{2}$ ) were computed as:


(9) $${\hat{h}}_{ind}^{2}={\hat{r}}_{\mathrm{Ba}}\bar{{\hat{\sigma }}_{a}^{2}}/\left(\bar{{\hat{\sigma }}_{p}^{2}}+\bar{{\hat{\sigma }}_{a}^{2}}+\bar{{\hat{\sigma }}_{d}^{2}}+\bar{{\hat{\sigma }}_{e}^{2}}\right)$$

(10) $${\hat{d}}_{ind}^{2}={\hat{r}}_{\mathrm{Bd}}\bar{{\hat{\sigma }}_{d}^{2}}/\left(\bar{{\hat{\sigma }}_{p}^{2}}+\bar{{\hat{\sigma }}_{a}^{2}}+\bar{{\hat{\sigma }}_{d}^{2}}+\bar{{\hat{\sigma }}_{e}^{2}}\right)$$

(11) $${\hat{H}}_{ind}^{2}=({\hat{r}}_{\mathrm{Ba}}\bar{{\hat{\sigma }}_{a}^{2}}+{\hat{r}}_{\mathrm{Bd}}\bar{{\hat{\sigma }}_{d}^{2}})/\left(\bar{{\hat{\sigma }}_{p}^{2}}+\bar{{\hat{\sigma }}_{a}^{2}}+\bar{{\hat{\sigma }}_{d}^{2}}+\bar{{\hat{\sigma }}_{e}^{2}}\right)$$

where $\bar{{\hat{\sigma }}_{d}^{2}}$ is the average dominance variance of the two sites, and ${\hat{r}}_{\mathrm{Bd}}$ is the correlation of dominance effects between sites. The narrow-sense type-B genetic correlation between sites was simply given by the estimated parameter ${\hat{r}}_{\mathrm{Ba}}$ , and the broad-sense type-B genetic correlation was calculated as:


(12) $${\hat{r}}_{\mathrm{Bg}}=\frac{{\hat{r}}_{\mathrm{Ba}}\sqrt{{\hat{\sigma }}_{a\_ASS}^{2}\;{\hat{\sigma }}_{a\_SCA}^{2}}+\;{\hat{r}}_{\mathrm{Bd}}\sqrt{{\hat{\sigma }}_{d\_ASS}^{2}\;{\hat{\sigma }}_{d\_SCA}^{2}}\;}{\sqrt{\left({\hat{\sigma }}_{a\_ASS}^{2}+{\hat{\sigma }}_{d\_ASS}^{2}\right)\left({\hat{\sigma }}_{a\_SCA}^{2}+{\hat{\sigma }}_{d\_SCA}^{2}\right)}}$$

It should be noted that the estimated broad-sense heritability and broad-sense type-B genetic correlation are approximations since epistatic effects were not modeled for this experimental design.

Standard errors of genetic parameter estimates were obtained using the delta method (“vpredict” function from the ASReml-R v.4.1 package). Estimated breeding values of individual trees on each site were obtained as the best linear unbiased predictions (BLUPs) of the random additive effect (***a*(s)**). Estimated genetic values of individual trees on each site were obtained by adding the dominance deviations (BLUPs of the dominance effect ***d*(*s*)**) to the breeding values.

### 2.4 Cross-validations

The predictive ability (*PA*) and prediction accuracy (*PACC*) of ABLUP and GBLUP models were estimated using two cross-validation (CV) procedures. The CV1 scenario evaluated the potential for predicting additional unphenotyped progeny trees within the same families as those included in the training population, while the CV2 scenario evaluated the prediction of new unphenotyped full-sib families. For CV1, trees were randomly split into 10 folds, making sure that each fold contained ~10% of the trees from each family (i.e., folding within families). For CV2, families were randomly split into 10-folds such that there were only half-sib relationships between the training and validation datasets (i.e., folding over families). For each round of CV, nine folds were used in model training, which was used to predict the breeding and genetic values for the remaining fold (i.e., the validation dataset). This 10-fold cross-validation was repeated 10 times to obtain the standard deviation of estimates.

All *PA* and *PACC* estimates were calculated across folds (i.e., using the predicted breeding and genetic values from all the sampled trees), within each repetition (Legarra et al., 2008). For each repetition, estimates of *PA* and *PACC* were first calculated within each site separately, and then averaged across sites. This procedure was done because each site had different heritabilities (see results), which can in turn affect *PA* and *PACC* estimates. We reported the mean and standard deviation of *PA* and *PACC* estimates across repetitions.

The predictive ability of the models was evaluated as the Pearson’s correlation coefficient between the predicted breeding (*PA_BV_*) or total genetic values (*PA_GV_*) and the observed phenotypes, within each site. The prediction accuracy of breeding value estimates (*PACC_BV_*) was obtained as $PACC_{BV}=PA_{BV}/\sqrt{{\hat{h}}_{ind}^{2}}$ (Dekkers, 2007; Legarra et al., 2008), where ${\hat{h}}_{ind}^{2}$ is the within-site heritability estimate. Similarly, the prediction accuracy of genetic value estimates (*PACC_GV_*) was obtained as $PACC_{GV}=PA_{GV}/\sqrt{{\hat{H}}_{ind}^{2}}$ . For the calculation of *PACC_BV_* and *PACC_GV_* of both ABLUP and GBLUP models, we used the within-site ${\hat{h}}_{ind}^{2}$ and ${\hat{H}}_{ind}^{2}$ estimated from the corresponding GBLUP models and using 100% of samples, thus representing our best estimates of the “true” narrow-sense and broad-sense heritability, respectively (**Tables S4–S7**). Hence, comparisons of *PACC* between the corresponding ABLUP and GBLUP models were possible because we used the same heritability estimates for both models.

### 2.5 Resampling simulations

To investigate the ability of ABLUP-AD and GBLUP-AD models to estimate additive and dominance effects at different sample sizes, we ran two scenarios of resampling simulations: 1) varying the number of families while keeping the number of trees per family constant, and 2) varying the number of trees per family while keeping constant the number of families. For this analysis, we used dataset 1 (BGs 1, 2, 5, and 6) and identified 72 families in which at least 26 trees per family were sampled. This was the number of trees per family that allowed us to keep the largest maximum number of trees for these resampling simulations (*n* = 1,872). This subset of 72 families was used for the two resampling scenarios.

For the first resampling scenario, we randomly sampled 12, 20, 28, 36, 48, and 60 families, and compared the results with those using all 72 families. To ensure roughly equal representation of families across BGs, the families were sampled in proportions of the status number in each BG, such that BGs with larger effective population sizes had a larger number of families sampled. Then, we sampled 26 individuals per family to keep the number of trees per family constant. For the second scenario, we randomly sampled 6, 8, 12, 16, 20, and 26 trees per family in each of the 72 families. For each scenario, resampling was repeated 10 times. For each repetition, the ***A, D, G_a_***, and ***G_d_*** matrices were recalculated based only on the sampled trees, and the ABLUP-AD and GBLUP-AD models were run to re-estimate the genetic parameters of interest, that is ${\hat{H}}_{ind}^{2}$ , ${\hat{d}}_{ind}^{2}$ , and ${\hat{H}}_{ind}^{2}$ .

In each repetition, a 10-fold cross-validation using CV2 (i.e., folding over families) was conducted to estimate *PA_GV_* and *PACC_GV_* because it simulates the most interesting use of GS, that is making new crosses for the next-generation or predicting new parental combinations for mating allocation. For the first resampling scenario, in which we varied the number of families sampled, folding over families resulted in ~90% of families in the training dataset, that is 11, 18, 25, 32, 43, 54, and 65 families. For the calculation of *PACC_GV_* of both ABLUP-AD and GBLUP-AD, we used the ${\hat{H}}_{ind}^{2}$ estimated from the GBLUP-AD models trained with all the trees available from the 72 families. Estimates of heritability, *PA_GV_*, and *PACC_GV_* were then averaged across the 10 resampling repetitions. The models that did not converge were not included in the calculations of *PA_GV_* and *PACC_GV_*.

## 3 Results

### 3.1 Genetic parameter estimates

Genetic parameters were estimated by modeling additive effects in the pedigree-based ABLUP-A and genomic-based GBLUP-A models, or by modeling both additive and dominance genetic effects in ABLUP-AD and GBLUP-AD. In both datasets, the within-site narrow-sense and broad-sense heritabilities differed between sites (**Tables S4**–**S7** contain the variance components and within-site heritability estimates obtained for each dataset and model). For simplicity, we focus on the across-site heritability estimates.

**Figure 2:**
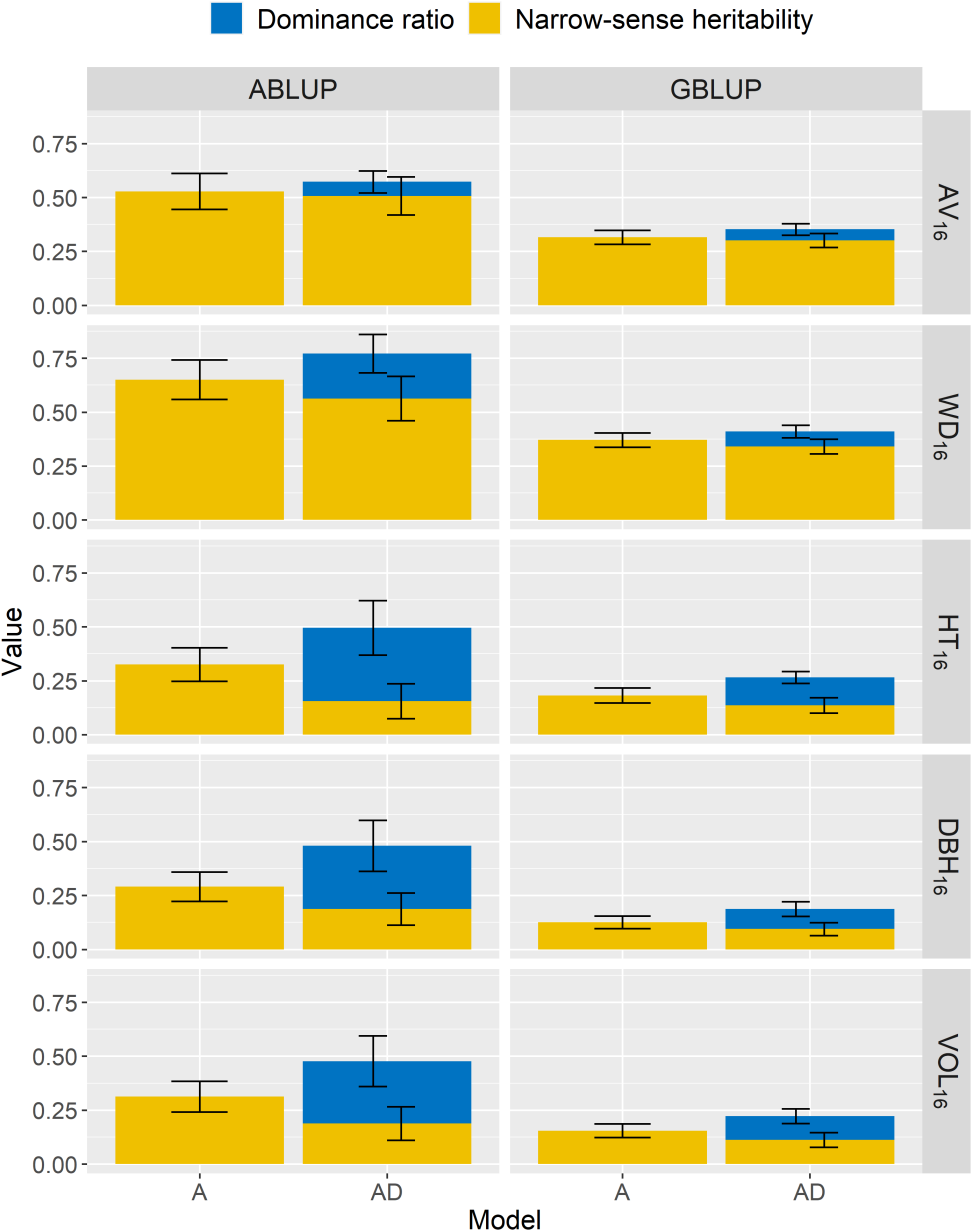
For the complete dataset 1, across-site narrow-sense heritabilities ( ${\hat{h}}_{ind}^{2}$ ) and dominance ratios ( ${\hat{d}}_{ind}^{2}$ ) estimated using additive (A) or additive-dominance (AD) models, with ABLUP or GBLUP. The broad-sense heritabilities $({\hat{H}}_{ind}^{2}$ ) are estimated as the sum of ${\hat{h}}_{ind}^{2}$ and ${\hat{d}}_{ind}^{2}$ . The error bars represent the approximated standard errors of estimates calculated using the delta method. See **Table 2** for a description of traits.

For the first dataset and the additive models, the two wood traits, that is acoustic velocity (AV_16_) and average wood density (WD_16_), were moderately to highly heritable (ABLUP-A: ${\hat{h}}_{ind}^{2}$ =0.53–0.65; GBLUP-A: ${\hat{h}}_{ind}^{2}$ =0.32–0.37), while the growth traits height (HT_16_), DBH_16_, and volume (VOL_16_) exhibited low to moderate heritabilities (ABLUP-A: ${\hat{h}}_{ind}^{2}$ =0.29–0.33; GBLUP-A: ${\hat{h}}_{ind}^{2}$ =0.13–0.18; **Table 3**; **Figure 2**). Similar results were found for the additive-dominance ABLUP-AD and GBLUP-AD models, that is higher narrow-sense and broad-sense heritabilities for wood than growth traits. The ABLUP-AD models estimated high broad-sense heritabilities ( ${\hat{H}}_{ind}^{2}$ ), ranging from 0.48 for DBH_16_ and VOL_16_ to as much as 0.77 for WD_16_. The estimates of ${\hat{H}}_{ind}^{2}$ obtained using GBLUP-AD were considerably lower and varied from 0.19 for DBH_16_ to 0.41 for WD_16_. The two wood traits showed very low genotype-by-environment interactions (GxE), as indicated by high narrow-sense ( ${\hat{r}}_{\mathrm{Ba}}$ >0.98) and broad-sense ( ${\hat{r}}_{\mathrm{Bg}}$ >0.92) type-B genetic correlations, while moderately higher GxE was found for growth traits under the additive or additive-dominance models.

**Table 3:** For the complete dataset 1, across-site genetic parameters estimated using the ABLUP and GBLUP additive (A) and additive-dominance (AD) models.

	AV_16_		WD_16_		HT_16_		DBH_16_		VOL_16_	
	A^1^	AD^2^	A^1^	AD^2^	A^1^	AD^2^	A^1^	AD^2^	A^1^	AD^2^
ABLUP										
${\hat{h}}_{ind}^{2}$	0.53 (0.08)	0.51 (0.09)	0.65 (0.09)	0.56 (0.10)	0.33 (0.08)	0.16 (0.08)	0.29 (0.07)	0.19 (0.07)	0.31 (0.07)	0.19 (0.08)
${\hat{d}}_{ind}^{2}$	—	0.07 (0.05)	—	0.21 (0.09)	—	0.34 (0.13)	—	0.29 (0.12)	—	0.29 (0.12)
${\hat{H}}_{ind}^{2}$	—	0.57 (0.09)	—	0.77 (0.10)	—	0.50 (0.11)	—	0.48 (0.10)	—	0.48 (0.10)
${\hat{r}}_{\mathrm{Ba}}$	0.98 (0.03)	0.98 (0.03)	0.99 (0.02)	0.99 (NA)†	0.78 (0.09)	0.62 (0.19)	0.84 (0.09)	0.85 (0.18)	0.85 (0.08)	0.83 (0.17)
${\hat{r}}_{\mathrm{Bg}}$	—	0.98 (0.03)	—	0.96 (0.05)	—	0.80 (0.10)	—	0.79 (0.11)	—	0.80 (0.11)
AIC	**-2949**	-2945	17897	**17880**	24370	**24348**	16521	**16493**	15091	**15065**
BIC	**-2897**	-2876	17949	17950	24422	**24417**	16573	**16563**	15143	**15134**
${\hat{V}}_{A}$ (%)^3^	—	89	—	73	—	31	—	39	—	39
${\hat{V}}_{D}$ (%)^3^	—	11	—	27	—	69	—	61	—	61
GBLUP										
${\hat{h}}_{ind}^{2}$	0.32 (0.03)	0.30 (0.03)	0.37 (0.03)	0.34 (0.03)	0.18 (0.03)	0.14 (0.04)	0.13 (0.03)	0.09 (0.03)	0.15 (0.03)	0.11 (0.03)
${\hat{d}}_{ind}^{2}$	—	0.05 (0.03)	—	0.07 (0.03)	—	0.13 (0.03)	—	0.09 (0.03)	—	0.11 (0.03)
${\hat{H}}_{ind}^{2}$	—	0.35 (0.04)	—	0.41 (0.04)	—	0.27 (0.04)	—	0.19 (0.04)	—	0.22 (0.04)
${\hat{r}}_{\mathrm{Ba}}$	0.99 (0.04)	0.99 (NA)†	0.99 (0.04)	0.99 (NA)†	0.73 (0.11)	0.63 (0.13)	0.73 (0.14)	0.70 (0.20)	0.77 (0.12)	0.67 (0.17)
${\hat{r}}_{\mathrm{Bg}}$	—	0.99 (NA)†	—	0.92 (0.07)	—	0.76 (0.09)	—	0.67 (0.14)	—	0.80 (0.14)
AIC	-2948	-2946	17892	**17874**	24380	**24342**	16538	**16496**	15105	**15069**
BIC	**-2896**	-2876	17944	17943	24433	**24412**	16590	**16565**	15157	**15139**
${\hat{V}}_{A}$ (%)^3^	—	85	—	83	—	51	—	50	—	50
${\hat{V}}_{D}$ (%)^3^	—	15	—	17	—	49	—	50	—	50

The parameters reported are individual narrow-sense ( ${\hat{h}}_{ind}^{2}$ ) and broad-sense heritability ( ${\hat{H}}_{ind}^{2}$ ), the dominance ratio ( ${\hat{d}}_{ind}^{2}$ ), and the narrow-sense ( ${\hat{r}}_{\mathrm{Ba}}$ ) and broad-sense ( ${\hat{r}}_{\mathrm{Bg}}$ ) type-B genetic correlations. Standard errors of estimates are in parentheses. The AIC and BIC of the best models between the A and AD models are in bold (Δ>2). The details of all variance components and within-site genetic parameter estimates are in **Table S4** (A models) and **Table S5** (AD models). See **Table 2** for a description of traits.

^1^ The additive model fitted is described in Eq. [7], by excluding the dominance term ***d(s)***. ^2^ The additive-dominance model fitted is described in Eq. [7].

^3^ The across-site additive ( ${\hat{V}}_{A}={\hat{r}}_{\mathrm{Ba}}\bar{{\hat{\sigma }}_{a}^{2}}$ ) and dominance variances ( ${\hat{V}}_{D}={\hat{r}}_{\mathrm{Bd}}\bar{{\hat{\sigma }}_{d}^{2}}$ ) are reported as a percentage of the total genetic variance ( ${\hat{V}}_{A}+{\hat{V}}_{D}$ ).

† These terms were at boundary (very close to 1) after convergence of the model.

For wood traits and dataset 1, only small dominance effects were detected (**Table 3**; **Figure 2**). For AV_16_, the dominance ratio was close to zero ( ${\hat{d}}_{ind}^{2}$ <0.07) for both ABLUP-AD and GBLUP-AD, and the smaller AIC and BIC values indicated that the additive models were more parsimonious than the additive-dominance models. For WD_16_, the AIC favored the additive-dominance models, but the BIC was similar between the additive and additive-dominance models. The dominance ratio was moderate under ABLUP-AD ( ${\hat{d}}_{ind}^{2}$ =0.21), but low under GBLUP-AD ( ${\hat{d}}_{ind}^{2}$ =0.07). Nevertheless, the dominance variance for WD_16_ represented a small proportion of the total genetic variance (ABLUP-AD: 27%; GBLUP-AD: 17%).

For growth traits and dataset 1, both the AIC and BIC values favored the additive-dominance models over the additive models (**Table 3**). The dominance ratios were moderate for ABLUP-AD ( ${\hat{d}}_{ind}^{2}$ =0.29–0.34) or low for GBLUP-AD ( ${\hat{d}}_{ind}^{2}$ =0.09–0.13). For growth traits under ABLUP-AD, the dominance variance represented the majority (61%–69%) of the total genetic variance (**Table 3**). In contrast, under GBLUP-AD, the dominance variance was about equal to the additive variance.

For the second dataset, we found results similar to those of dataset 1, that is moderate to high across-site heritabilities for wood traits, while growth traits presented lower heritabilities (**Table S8**; **Figure S5**). The genetic variation in wood traits was mostly due to additive effects, with null or low dominance effects, and low GxE. For growth traits, dominance ( ${\hat{d}}_{ind}^{2}$ ) represented the largest portion of the total genetic variance under both ABLUP-AD (63%–85%) and GBLUP-AD (60%–82%). Again, for both datasets, heritability estimates obtained using GBLUP-AD were markedly lower than those obtained using ABLUP-AD.

### 3.2 Predictive ability and accuracy of the additive models

The predictive abilities of breeding value estimates (*PA_BV_*), that is the correlation between the predicted breeding values and the observed phenotypes, considerably differed between sites for most traits. For simplicity, we present the averaged estimates across sites. For the first cross-validation scenario (CV1; i.e., folding within families) in dataset 1 and the additive ABLUP-A and GBLUP-A models, we found *PA_BV_* values ranging from 0.48–0.55 for wood traits, and from 0.36–0.39 for growth traits (**Table 4**). After standardizing by the square root of heritability, the prediction accuracies of breeding values (*PACC_BV_*) were similar between wood (*PACC_BV_* =0.84–0.90) and growth traits (*PACC_BV_* =0.76–0.91).

**Table 4:** For the complete dataset 1, average across-site predictive ability (*PA*) and prediction accuracy (*PACC*) obtained from cross-validation using the ABLUP and GBLUP additive (A) and additive-dominance (AD) models.

	AV_16_		WD_16_		HT_16_		DBH_16_		VOL_16_	
	A^1^	AD^2^	A^1^	AD^2^	A^1^	AD^2^	A^1^	AD^2^	A^1^	AD^2^
ABLUP										
CV1^3^										
*PA_BV_* ^4^	0.48 (0.01)	0.48 (0.01)	0.54 (0.03)	0.54 (0.03)	0.39 (0.03)	0.38 (0.02)	0.38 (0.04)	0.36 (0.05)	0.38 (0.03)	0.37 (0.03)
*PA_GV_* ^4^	—	0.48 (0.01)	—	0.55 (0.03)	—	0.41 (0.04)	—	0.41 (0.04)	—	0.41 (0.03)
*PACC_BV_* ^5^	0.84 (0.02)	0.86 (0.03)	0.88 (0.03)	0.92 (0.04)	0.78 (0.02)	0.81 (0.03)	0.91 (0.01)	1.00 (0.02)	0.87 (0.01)	0.93 (0.01)
*PACC_GV_* ^5^	—	0.80 (0.03)	—	0.82 (0.01)	—	0.70 (0.01)	—	0.78 (0.02)	—	0.79 (0.01)
CV2^3^										
*PA_BV_* ^4^	0.41 (0.02)	0.40 (0.02)	0.43 (0.02)	0.43 (0.02)	0.22 (0.02)	0.19 (0.03)	0.20 (0.05)	0.20 (0.05)	0.21 (0.03)	0.19 (0.04)
*PA_GV_* ^4^	—	0.40 (0.02)	—	0.43 (0.02)	—	0.19 (0.03)	—	0.20 (0.05)	—	0.19 (0.04)
*PACC_BV_* ^5^	0.72 (0.03)	0.73 (0.04)	0.71 (0.03)	0.74 (0.04)	0.44 (0.04)	0.42 (0.05)	0.48 (0.08)	0.53 (0.07)	0.47 (0.06)	0.47 (0.07)
*PACC_GV_* ^5^	—	0.68 (0.03)	—	0.65 (0.02)	—	0.33 (0.05)	—	0.37 (0.08)	—	0.36 (0.06)
GBLUP										
CV1^3^										
*PA_BV_* ^4^	0.48 (0.02)	0.48 (0.02)	0.55 (0.03)	0.54 (0.03)	0.38 (0.03)	0.37 (0.03)	0.36 (0.05)	0.35 (0.05)	0.37 (0.03)	0.36 (0.03)
*PA_GV_* ^4^	—	0.48 (0.02)	—	0.55 (0.03)	—	0.41 (0.04)	—	0.40 (0.04)	—	0.40 (0.03)
*PACC_BV_* ^5^	0.84 (0.01)	0.86 (0.01)	0.90 (0.03)	0.94 (0.04)	0.76 (0.03)	0.80 (0.05)	0.87 (0.02)	0.95 (0.02)	0.84 (0.01)	0.90 (0.01)
*PACC_GV_* ^5^	—	0.80 (0.01)	—	0.83 (0.01)	—	0.70 (0.03)	—	0.75 (0.03)	—	0.76 (0.01)
CV2^3^										
*PA_BV_* ^4^	0.42 (0.02)	0.41 (0.02)	0.45 (0.03)	0.45 (0.03)	0.22 (0.03)	0.23 (0.03)	0.22 (0.06)	0.24 (0.05)	0.22 (0.04)	0.24 (0.04)
*PA_GV_* ^4^	—	0.41 (0.02)	—	0.46 (0.02)	—	0.26 (0.03)	—	0.29 (0.04)	—	0.28 (0.03)
*PACC_BV_* ^5^	0.74 (0.02)	0.75 (0.02)	0.74 (0.03)	0.78 (0.04)	0.44 (0.04)	0.49 (0.05)	0.52 (0.08)	0.64 (0.06)	0.50 (0.06)	0.59 (0.05)
*PACC_GV_* ^5^	—	0.69 (0.02)	—	0.68 (0.02)	—	0.45 (0.03)	—	0.55 (0.04)	—	0.53 (0.03)

Standard deviations of estimates across repetitions are in parentheses. For the ABLUP-AD models and CV2, the predicted dominance deviations were null for all individuals, such that the predicted genetic values were equal to the predicted breeding values. Hence, the *PA_BV_* was equal to *PA_GV_*. See **Table 2** for a description of traits.

^1^ The additive model fitted is described in Eq. [7], by excluding the dominance term ***d(s)***. ^2^ The additive-dominance model fitted is described in Eq. [7].

^3^ CV1: trees were randomly split into 10 folds, making sure that each fold contained ~10% of the trees from each family; CV2: families were randomly split into 10 folds.

^4^ The predictive ability of breeding values (*PA_BV_*) and of genetic values (*PA_GV_*) were calculated as the correlation between the predicted breeding or genetic values and the observed phenotypes, respectively, within each site. The average across sites is reported.

^5^ The prediction accuracy of breeding values (*PACC_BV_*) was equal to $PA_{BV}/\sqrt{{\hat{h}}_{ind}^{2}}$ . The prediction accuracy of genetic values (*PACC_GV_* ) was equal to $PA_{GV}/\sqrt{{\hat{H}}_{ind}^{2}}$ . The heritabilities obtained using GBLUP were used to calculate the *PACC* of ABLUP and GBLUP models. The average across sites is reported.

To compare ABLUP and GBLUP models, we simply use the predictive ability (*PA*). The results were identical in terms of prediction accuracy (*PACC*) since we used the same heritability estimates for both methods. For CV1, The *PA_BV_* were similar between ABLUP-A and GBLUP-A for all traits.

For the second cross-validation scenario (CV2) in dataset 1, in which the predicted unphenotyped full-sib families were not part of the model training (i.e., folding over families), the *PA_BV_* and *PACC_BV_* were smaller than for CV1 (**Table 4**). Under CV2, the reduction was more important for growth (*PA_BV_* =0.20–0.22) than for wood traits, with *PA_BV_* values remaining above 0.41. These results translated into much smaller *PACC_BV_* for growth (0.44–0.52) than for wood traits (0.71–0.74) under CV2. Again, we obtained similar *PA_BV_* values between ABLUP-A and GBLUP-A for all traits.

The results for dataset 2 were similar to those of dataset 1 (**Table S9**). We found no clear advantage of GBLUP-A versus ABLUP-A in terms of predictive ability or accuracy of breeding values for both CV1 and CV2 scenarios. Under CV2, we also found much larger *PA_BV_* and *PACC_BV_* estimates for wood than for growth traits.

### 3.3 Predictive ability and accuracy of the additive-dominance models

For dataset 1 under CV1, and for both ABLUP and GBLUP, the additive-dominance models improved the predictive ability of genetic values (breeding values + dominance deviations, *PA_GV_*) for growth traits by 0.02–0.04 compared with that of breeding values (*PA_BV_*) from the additive models, but little to no improvement was observed for wood traits (increase of 0–0.01; **Table 4**). However, all traits showed a reduction of 0.04–0.13 of prediction accuracy of genetic values (*PACC_GV_*) under the additive-dominance models, after standardizing by the broad-sense heritability, compared with that of breeding values (*PACC_BV_*) under the additive models, which were standardized by the narrow-sense heritability.

When predicting for new unphenotyped families under CV2, the *PA_GV_* of GBLUP-AD models was again larger than the *PA_BV_* of GBLUP-A models for growth traits (increase of 0.04–0.07), but not for wood traits (increase of 0–0.01; **Table 4**). This increasing trend in favor of GBLUP-AD versus GBLUP-A for growth traits was also observed for prediction accuracies (*PACC_GV_* versus *PACC_BV_*; increase of 0.01–0.03). We found the opposite trend for ABLUP models under CV2, with a reduction of 0–0.03 from *PA_BV_* (ABLUP-A) to *PA_GV_* (ABLUP-AD), and a large reduction of 0.11 from *PACC_BV_* to *PACC_GV_* for the three growth traits.

Under CV1, we found almost equal predictive ability of genetic values (*PA_GV_*) between ABLUP-AD and GBLUP-AD (**Table 4**). However, when predicting for unphenotyped families (CV2), there was a clear advantage of GBLUP-AD over ABLUP-AD for *PA_GV_*. The advantage of GBLUP-AD over ABLUP-AD for predicting genetic values was larger for growth (increase of up to 0.09 in *PA_GV_*) than for wood traits (increase of up to 0.03 in *PA_GV_*). Interestingly, GBLUP-AD also increased the predictive ability of breeding values (*PA_BV_*) over ABLUP-AD, again only under CV2, and especially for growth traits (increase of *PA_BV_* up to 0.05). Thus, GBLUP-AD was better than ABLUP-AD for predicting both breeding and genetic values for unphenotyped families.

For dataset 2, we obtained very similar results (**Table S9**). Most importantly, for growth traits, modeling dominance with GBLUP-AD and predicting the total genetic values for unphenotyped families (CV2) was clearly advantageous compared with predicting only breeding values with GBLUP-A. In contrast, a reduction of predictive ability and accuracy was observed when comparing ABLUP-AD with ABLUP-A models. Furthermore, GBLUP-AD outperformed ABLUP-AD under the CV2 scenario for growth traits. For example, the *PA_GV_* almost doubled using GBLUP-AD versus ABLUP-AD for DBH (ABLUP-AD: *PA_GV_* =0.11; GBLUP-AD: *PA_GV_* =0.20) and volume (ABLUP-AD: *PA_GV_* =0.12; GBLUP-AD: *PA_GV_* =0.20).

### 3.4 Varying the sample size

**Figure 3:**
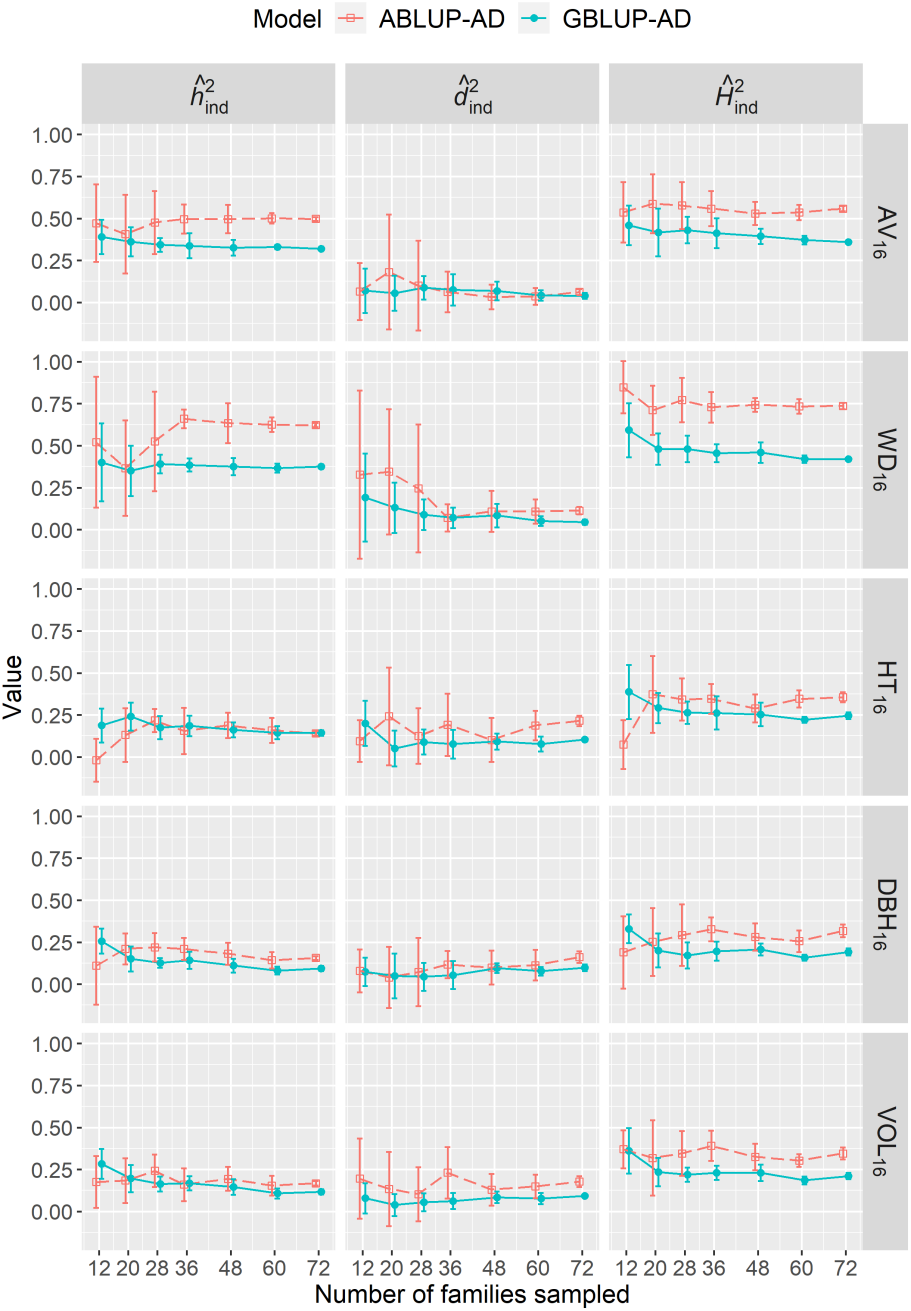
For dataset 1, across-site narrow-sense heritabilities ( ${\hat{h}}_{ind}^{2}$ ), dominance ratios ( ${\hat{d}}_{ind}^{2}$ ), and broad-sense heritabilities $({\hat{H}}_{ind}^{2}$ ) estimated when varying the number of families sampled using the ABLUP-AD and GBLUP-AD additive-dominance models. The error bars represent the standard deviations of estimates across the 10 replications for each level of number of families sampled. See **Table 2** for a description of traits.

We varied the number of full-sib families sampled from 12 to 72 in dataset 1 to determine the effect of sample size on genetic parameter estimates. For the majority of sample sizes, either the narrow-sense heritability ( ${\hat{h}}_{ind}^{2}$ ; e.g., AV_16_, WD_16_), the dominance ratio ( ${\hat{d}}_{ind}^{2}$ ; e.g., HT_16_), or sometimes both parameters (e.g., DBH_16_, VOL_16_) were generally overestimated using ABLUP-AD, leading to higher broad-sense heritabilities ( ${\hat{H}}_{ind}^{2}$ ) compared with GBLUP-AD (**Figure 3**). Most importantly, we found that genetic parameter estimates were more stable at all sample sizes using GBLUP-AD compared with ABLUP-AD. The standard deviations of ${\hat{h}}_{ind}^{2}$ , ${\hat{d}}_{ind}^{2}$ , and ${\hat{H}}_{ind}^{2}$ estimates were on average 36%, 48%, and 35% smaller, respectively, using GBLUP-AD compared with ABLUP-AD. For GBLUP-AD, estimates of ${\hat{H}}_{ind}^{2}$ had very small standard deviations when 60 families or more were sampled, while the ABLUP-AD models required using all 72 families to obtain similarly small standard deviations.

**Figure 4:**
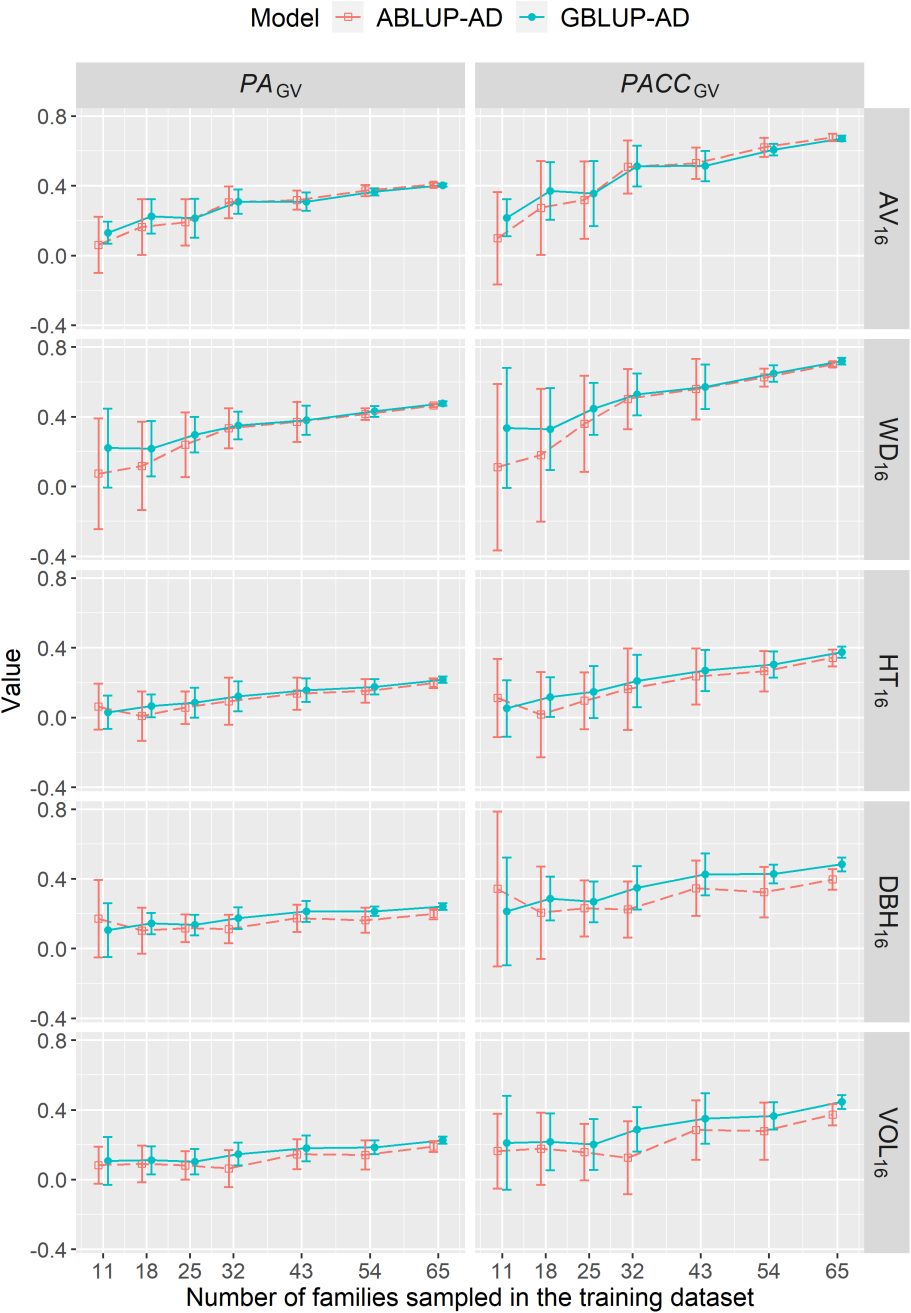
For dataset 1, average across-site predictive ability (*PA_GV_*) and prediction accuracy of genetic values (*PACC_GV_*) estimated using CV2 and when varying the number of families sampled in the training dataset using the ABLUP-AD and GBLUP-AD additive-dominance models. The error bars represent the standard deviations of estimates across the 10 replications for each level of number of families sampled. See **Table 2** for a description of traits.

The predictive ability (*PA_GV_*) or accuracy (*PACC_GV_*) of genetic values under CV2 steadily increased with increasing the number of families sampled in the training dataset up to the maximum number of families (**Figure 4**). Depending on the trait, the *PACC_GV_* increased by 53%–86% and by 14%–86% using ABLUP-AD and GBLUP-AD, respectively. For growth traits, the *PA_GV_* of GBLUP-AD were generally higher than those of ABLUP-AD across all sample sizes. For wood traits, GBLUP-AD was better than ABLUP-AD when 18 families or less were sampled in the training dataset, but they performed similarly for larger sample sizes. Finally, the standard deviations of *PA_GV_* estimates were on average 27% smaller using GBLUP-AD compared with ABLUP-AD.

We found similar results when varying the number of trees per family while keeping constant the number of families. Increasing the number of trees per family decreased standard deviations of $H_{ind}^{2}$ estimates up to the maximum of 26 trees per family using both ABLUP-AD and GBLUP-AD (**Figure S6**). Again, standard deviations of estimates were generally smaller under GBLUP-AD than ABLUP-AD. In cross-validations, the increase in *PACC*_GV_ from 6 to 26 trees per family (ABLUP: 6%–43%; GBLUP: 8%–40%; **Figure S7**) was less pronounced than when varying the number of families. *PA_GV_* and *PACC_GV_* reached a plateau at around 12 (AV_16_, WD_16_, DBH_16_, VOL_16_) or 20 (HT_16_) trees per family for both ABLUP-AD and GBLUP-AD.

## 4 Discussion

### 4.1 Wood traits are good candidates for tree breeding, with mostly additive inheritance and low GxE

We found that the studied wood traits, acoustic velocity, a proxy for wood stiffness, and average wood density, were under moderate to high genetic control, with almost no GxE interactions, indicating very little rank changes of families between the two contrasting environments tested. These conclusions based on GxE are limited as we only considered two sites. However, these results were confirmed in the two datasets analyzed here, which can be seen as independent replications since they consisted of different breeding populations with no relatedness between them. Our results are also in line with previous studies, which generally found higher across-site heritability and lower GxE estimates for wood quality versus growth traits in white spruce (Beaulieu et al., 2014b, 2020; Lenz et al., 2020a), Norway Spruce (Chen et al., 2018; Lenz et al., 2020b; Nguyen et al., 2022), lodgepole pine (Ukrainetz and Mansfield, 2020), Douglas-fir (Ukrainetz et al., 2008), and radiata pine (Baltunis et al., 2010; Raymond, 2011), with some exceptions (e.g., in interior spruce, Gamal El-Dien et al., 2015, 2018; in black spruce, Lenz et al., 2017). Thus, evidence is increasing for many conifer species that wood traits are moderately to highly heritable, with low GxE, compared with growth traits (Beaulieu and Bousquet, 2010; Hassegawa et al., 2020).

Our results for the second dataset can be directly compared to those of Beaulieu et al. (2014b) given that the same genotypic and phenotypic data (WD_13_, HT_13_, and DBH_13_) were used, although we performed additional SNP and individual quality control filtering following pedigree correction. The reported within-site narrow-sense heritabilities using the ABLUP-A models in Beaulieu et al. (2014b) were in the same range as in this study (**Table S6**). In both studies, the within-site heritabilities were moderate to high for wood and growth traits, but a lower GxE component was found for wood than for growth traits, emphasizing the importance of multi-site analyses and reporting the across-site heritability estimates.

In this study, we further separated the genetic variance into additive and dominance effects. We found that, in both analyzed datasets, acoustic velocity and wood density exhibited small dominance effects, accounting for 15%–17% of the total genetic variance in the genomic GBLUP-AD models. However, the dominance effects were close to 0 considering the standard errors of estimates, and the AIC and BIC generally favored the additive models (GBLUP-A) over the additive-dominance models (GBLUP-AD) for both datasets. The literature is sparse regarding the evaluation of non-additive effects, especially in white spruce (Weng et al., 2008), and even more so for wood traits. A recent study that analyzed a multi-site full-sib trial reported moderate dominance for acoustic velocity ( ${\hat{d}}_{ind}^{2}$ =0.25), similar to the narrow-sense heritability ( ${\hat{h}}_{ind}^{2}$ =0.30), using GBLUP-AD (Beaulieu et al., 2020). These contrasting results across studies for acoustic velocity is not unexpected given that the decomposition of genetic variance into additive and dominance components is population specific as it depends on the population allele frequencies (Falconer and Mackay, 1996; Hill et al., 2008; Huang and Mackay, 2016). In full-sib or clonally replicated trials in other conifers, null or small dominance effects for wood traits were detected in Norway spruce (Chen et al., 2019, 2020; Nguyen et al., 2022) and in Scots pine (Calleja-Rodriguez et al., 2021). In the well-studied *Eucalyptus* species and their hybrids, the genetic variance of wood density was found to be mostly additive (Costa e Silva et al., 2004, 2009; Resende et al., 2017; Tan et al., 2018; Thumma et al., 2022). Thus, our results and those of previous studies point towards mostly additive inheritance for wood traits, although the presence of some dominance or epistasis at the gene level is possible (Beaulieu et al., 2011; Huang and Mackay, 2016).

High narrow-sense heritabilities make wood traits excellent candidates for genetic improvement. The additive genetic variation is of utmost importance to tree breeders because it can be utilized in a simple and efficient random mating design such as open-pollinated seed orchards for the deployment of improved genetic material. Considerable genetic gains can be achieved for wood traits under such conditions (Lenz et al., 2013; Rashidi-Jouybari et al., 2022). Here, we found that the selected material for wood traits should perform well in a wide variety of environments, as evidenced by the very low GxE observed across the two study sites located in different breeding zones (Li et al., 1997). Indeed, Beaulieu et al. (2014b) found moderately high accuracies for wood traits when predicting across sites, confirming that selections for wood traits could be successfully deployed across breeding zones. In particular, acoustic velocity is quick to assess on standing trees and is generally found uncorrelated or positively correlated with height growth (Beaulieu et al., 2020; Hassegawa et al., 2020), thus showing promise for simultaneous improvements in multi-trait selection schemes (Lenz et al., 2020b; Laverdière et al., 2022).

### 4.2 GBLUP better estimates additive and dominance effects for growth traits

Improvement for growth has been the main focus in most forest tree improvement programs, yet non-additive effects have not been frequently evaluated or used in tree breeding, including for white spruce (Weng et al., 2008; Beaulieu et al., 2020). Compared to wood traits, we found significant dominance variance for growth traits, which was of the same magnitude or even larger than the additive variance. Tangible dominance variance for growth traits has been commonly observed in tree species (de Almeida Filho et al., 2016; Resende et al., 2017; Tan et al., 2018; Chen et al., 2019; Beaulieu et al., 2020; Thumma et al., 2022). In this study, ABLUP-AD and GBLUP-AD appeared to differ in their ability to partition the genetic variance. In the first dataset, GBLUP-AD partitioned the genetic variance of growth traits into smaller and roughly equal additive and dominance variances, while ABLUP-AD assigned the largest proportion of genetic variance to dominance. Using a full-sib trial, Beaulieu et al. (2020) also found large dominance effects for height, DBH, and volume under ABLUP-AD ( ${\hat{d}}_{ind}^{2}$ =0.22–0.51), with close to zero additive effects, while GBLUP-AD partitioned the genetic variance into relatively equal additive ( ${\hat{h}}_{ind}^{2}$ =0.09–0.18) and dominance effects ( ${\hat{d}}_{ind}^{2}$ =0.10–0.14).

In this study, there was a large reduction in ${\hat{h}}_{ind}^{2}$ when dominance was included in the additive-dominance models compared with the additive models, indicating that additive and dominance effects were partly confounded. In dataset 1, this observed reduction in ${\hat{h}}_{ind}^{2}$ was more pronounced for ABLUP (reduction of 34%–52%) than for GBLUP (reduction of 22%–31%). In dataset 2, the reduction in ${\hat{h}}_{ind}^{2}$ from additive to additive-dominance models was even more drastic (ABLUP: reduction of 50%–74%; GBLUP: reduction of 47%–67%). Interestingly, the reduction in ${\hat{h}}_{ind}^{2}$ was the steepest under ABLUP for the trait that showed the highest dominance ratio in each dataset (HT_16_ in dataset 1; DBH_13_ in dataset 2), clearly showing the important confounding of genetic variances occurring in the ABLUP-AD models. The reduction of the estimated additive variance when non-additive effects are included in the model has also been reported previously (e.g., Muñoz et al., 2014; Bouvet et al., 2016; Tan et al., 2018). Such reduction should not occur if the genetic variance components were orthogonal (i.e., independent, Vitezica et al., 2013) as assumed in quantitative genetic theory (Falconer and Mackay, 1996). However, as noted by de Almeida Filho et al. (2019), important theoretical assumptions such as Hardy-Weinberg equilibrium, random mating, and linkage equilibrium do not hold in real breeding populations.

Previous empirical studies found that the additive, non-additive, and environmental variances were less confounded under GBLUP than under ABLUP after examining the sampling correlation matrix of variance components, although estimates of variance components were not orthogonal even under GBLUP (Muñoz et al., 2014; Bouvet et al., 2016; Gamal El-Dien et al., 2016; Tan et al., 2018). Furthermore, simulations showed that GBLUP-AD better estimates additive and dominance variances because it uses the observed genomic relationships rather than expected relationships based on the pedigree (Vitezica et al., 2013; de Almeida Filho et al., 2019). Our resampling simulations provided additional evidence that GBLUP-AD is better than ABLUP-AD in estimating additive and dominance variances. The genetic parameter estimates were more stable, with lower standard deviations at all sample sizes using GBLUP-AD compared with ABLUP-AD. In addition, ABLUP-AD suffered from convergence problems at lower sample sizes (**Figure S8**), indicating difficulties in estimating all model parameters. Taken together, we found that GBLUP-AD provided more realistic estimates of both the additive and dominance variances and better separated these effects than ABLUP-AD (also see Nazarian and Gezan, 2016).

Besides the fact that estimates of additive and dominance variances are not orthogonal in practical breeding populations, these variances cannot be directly interpreted in terms of the relative importance of additive and non-additive gene actions (Falconer and Mackay, 1996; Hill et al., 2008; Huang and Mackay, 2016). Vitezica et al. (2013) described a matrix of dominant genomic relationships that can be used in a mixed model framework, such that the variances obtained using this “classical” or “breeding” parametrization can be directly compared to pedigree-based models. Under this parametrization of GBLUP-AD, the additive variance is prioritized over non-additive components, such that the statistical genetic variance decomposition does not reflect the biological or functional effects of the genes (Huang and Mackay, 2016). This is because the majority of dominant or epistatic gene actions (functional effects) contribute to additive genetic variance (statistical effects) in various ways depending on the allele frequencies in the population (Vitezica et al., 2013; Huang and Mackay, 2016). Indeed, Weng et al. (2008) used a large clonally replicated trial and pedigree-based models to infer that the additive variance accounted for ~80% of the total genetic variance for growth traits in white spruce, with dominance explaining most of the remaining genetic variance, and thus with little epistatic effects. The results of Weng et al. (2008) are in line with theory and empirical observations that additive variance is generally the major source of genetic variation for complex traits (Hill et al., 2008).

In any case, genetic variance analysis and the estimation of non-additive effects should remain highly useful for genetic predictions and selection in plant and animal breeding (Varona et al., 2018). Our results suggest that there exist significant non-additive effects for growth traits in white spruce because the broad-sense heritabilities found under the additive-dominance models were greater than the narrow-sense heritabilities under the additive models. Thus, the additive-dominance models captured additional genetic variance that was left aside by the additive models. Therefore, exploiting both additive and non-additive variances by propagating elite families or superior clones should prove to be beneficial and provide superior genetic gains for growth productivity in white spruce (Weng et al., 2008).

Recent studies attempted to decompose additive, dominance, and epistatic (ADE) variances using open-pollinated (Gamal El-Dien et al., 2016, 2018) or full-sib progeny trials (Tan et al., 2018; Chen et al., 2019; Calleja-Rodriguez et al., 2021). However, for the studies and traits that showed considerable epistatic variances, it was always associated with large standard errors, and, in all but one case (Gamal El-Dien et al., 2016), GBLUP-ADE was not the best model compared with GBLUP-AD or GBLUP-A based on AIC (Gamal El-Dien et al., 2018; Tan et al., 2018; Calleja-Rodriguez et al., 2021). Although gene-gene interactions have been found to be pervasive in model organisms (Mackay, 2014), there may be little power to detect epistatic variance for polygenic traits in practice (Hill et al., 2008; Mäki-Tanila and Hill, 2014). Clonally replicated trials are ideal to obtain accurate estimates of all genetic variance components in forest trees using either pedigree-based (Foster and Shaw, 1988; Wu, 1996) or genomic-based models (Muñoz et al., 2014; Walker et al., 2022). But even in such trials, a sufficient number of parents, full-sib families, genotypes per family, and clonal replication is required (Baltunis et al., 2009; Berlin et al., 2019; Nguyen et al., 2022). Given the difficulties mentioned above, we suggest that epistatic variance in white spruce should be estimated with a genomic-based model using only large clonally replicated trials.

### 4.3 The benefits of calculating predictive ability and accuracy within sites

When a completely independent dataset is unavailable to perform true model validation, cross-validation (CV) techniques can be used to evaluate model predictive ability and accuracy. The predictive ability (*PA*) is obtained as the correlation between the predicted breeding/genetic values and the observed phenotypes, and the prediction accuracy (*PACC*) is obtained as the correlation between the predicted breeding/genetic values and the true breeding/genetic values. In operational breeding populations, the true breeding/genetic values are unknown and many previous studies used, for this purpose, the BLUPs obtained using the complete dataset (i.e., using all phenotypic information) with either ABLUP or GBLUP as a surrogate for the true breeding values (e.g., Lenz et al., 2017; Ukrainetz and Mansfield, 2020; Walker et al., 2022). However, this is biased towards the method used to estimate those true values, and it can often result in an overestimation of the *PACC* (Beaulieu et al., 2020). We trust that *PA* and *PACC* calculated as $PA/\sqrt{h^{2}}$ are better estimators to compare the performance of ABLUP and GBLUP models since they do not require assumptions about the true breeding/genetic values. We used the heritability estimates obtained from GBLUP to calculate *PACC* for both the ABLUP and GBLUP models. Thus, the comparison of *PACC* between ABLUP and GBLUP models depended only on *PA*, not on the heritability estimates.

Here, we evaluated *PA* and *PACC* slightly differently than in previous GS studies in spruce (Beaulieu et al., 2020; Lenz et al., 2020a, 2020b). We used a two-site model that took GxE interaction into account to predict the breeding/genetic values of individual trees within each site, and estimated the correlation between those predicted breeding/genetic values and the observed phenotypes within each site separately. We then averaged the results across sites to simplify the presentation of the results. This was done for three main reasons. First, we used the raw phenotype, which was not adjusted for site effects, as a response variable in the mixed-models and fitted all fixed and random effects simultaneously. Second, each individual genotype was located in only one site because there was no clonal replication. By calculating *PA* and *PACC* within sites, there was a better correspondence between the observed phenotype for a particular individual and its predicted breeding/genetic value on that site than if the predicted breeding/genetic value represented an average across sites, such as commonly done when fitting a homogeneous genetic effect across sites (e.g., Beaulieu et al., 2020; Lenz et al., 2020a, 2020b). Third, the site St. Casimir (SCA) had generally larger heritability estimates than the site Asselin (ASS), and so it was expected that the *PA* and *PACC* would also differ between sites (Hayes et al., 2009). For example, for the first cross-validation scenario (CV1) using GBLUP-A and dataset 1, the *PA_BV_* was 8.70%–9.62% and 17.14%–29.03% larger at SCA versus ASS for wood and growth traits, respectively (**Table S10**). These differences were reduced for the *PACC_BV_* estimates, but were still considerable for some traits (e.g., -6.45% for wood density and +8.22% for height). Similar results were found for GBLUP-AD and for dataset 2. Given the differences observed between sites, we suggest that computing *PA* and *PACC* within sites as done here is more accurate, especially for traits with higher GxE, and when there is a sufficient number of samples per site.

### 4.4 GBLUP showed similar predictive ability compared to ABLUP when predicting within trained families

In our first CV scenario (CV1), individuals were divided into 10 folds, with ~10% of individuals from each family in each fold so that all families were well represented in training and validation datasets (i.e., folding within families). Using the CV1 scenario and the additive models, we obtained very high prediction accuracies of breeding values (*PACC_BV_*) for all traits, datasets, and models (ABLUP: 0.67–0.91; GBLUP: 0.69–0.90).

Comparing the performance of GS across studies is difficult because of the different methods used to estimate prediction accuracy in forest trees (Bousquet et al., 2021; Calleja-Rodriguez et al., 2021). However, several studies in spruce used similar CV schemes to those used here and calculated the predictive ability (*PA_BV_*) or accuracy (*PACC_BV_* as $PA/\sqrt{h^{2}}$ ) of breeding values using additive models (**Table 5**). The *PACC_BV_* is a better estimator to compare across studies because it accounts for the different heritabilities. We found that the accuracies obtained in this study for the GBLUP-A models and dataset 1 were similar to those reported for Norway spruce (Lenz et al., 2020b) or black spruce (Lenz et al., 2017) full-sib trials exhibiting lower genetic diversity, as measured by the status number (*Ns*). The accuracies obtained for both datasets in this study were larger than those obtained for a white spruce polycross trial with similar *Ns*, which used a smaller sample size (Lenz et al., 2020a). The *PACC_BV_* found in this study were also much larger than that obtained for other white spruce full-sib or open-pollinated trials with larger *Ns* (Beaulieu et al., 2014a, 2020), or with much smaller sample sizes (Laverdière et al., 2022). It should be noted that the markers used in the above-mentioned white spruce studies largely overlapped. Considering that the marker densities and trait heritabilities in all above-mentioned studies were in the same range, the higher accuracies of breeding values obtained in this study are likely due to the higher linkage disequilibrium existing in the breeding groups with small *Ns* and to the large training datasets that we used (Hayes et al., 2009; Grattapaglia and Resende, 2011). With the current marker density, our GBLUP models are mostly tracing relatedness through the large co-segregating haplotype blocks formed by controlled crossing, but probably not much of the short-range linkage disequilibrium between markers and QTLs (Beaulieu et al., 2014b; Lenz et al., 2017). As for the influence of the overall size of the training population, it was also clearly illustrated by a reduction of accuracy in dataset 2 compared with dataset 1.

**Table 5:** Summary of previous spruce studies that estimated the predictive ability of breeding values (*PA_BV_*: the correlation between the predicted breeding values and the phenotypes) using GBLUP-A models.

Study	Species	mating design	Cross-validation scheme^4^	*PACC_BV_*	*Ns*	Number of trees	Number of SNPs
Lenz et al. (2020b) ^1^	*Picea abies*	Full-sib (partial diallel)	Folding within families (10-folds)	0.86 (0.71–0.96)	24	714	3,934
**This study:** **dataset 1**	***Picea glauca***	**Full-sib** **(partial diallel)**	**Folding within families** **(10-folds; CV1)**	**0.84** **(0.76**–**0.90)**	**53**	**2,458**	**4,092**
Lenz et al. (2017) ^1,2,3^	*Picea mariana*	Full-sib (partial diallel)	Folding within families (10-folds)	0.81 (0.78–0.88)	19	734	4,993
**This study:** **dataset 2**	***Picea glauca***	**Full-sib** **(partial diallel)**	**Folding within families** **(10-folds; CV1)**	**0.71** **(0.69**–**0.76)**	**34**	**1,608**	**4,092**
Lenz et al. (2020a)	*Picea glauca*	Polycross	Folding within families (10-folds)	0.64 (0.61–0.70)	39	856	4,092
Beaulieu et al. (2020) ^1^	*Picea glauca*	Full-sib (partial diallel)	Folding within families (10-folds)	0.54 (0.38–0.67)	161	1,310	4,148
Beaulieu et al. (2014a) ^2^	*Picea glauca*	Open-pollinated	Folding within families (10-folds)	0.53 (0.40–0.74)	620	1,694	6,385
Laverdière et al. (2022) ^1^	*Picea glauca*	Polycross, site Watford	Folding within families (10-folds)	0.45 (0.27–0.57)	33	279	4,091
Laverdière et al. (2022) ^1^	*Picea glauca*	Polycross, site Normandin	Folding within families (10-folds)	0.41 (0.28–0.50)	31	281	4,091

The reported parameters are the average prediction accuracy of breeding values (*PACC_BV_*), with the range across the different traits shown in parentheses, the status number (*Ns*), the number of trees sampled, and the number of SNPs. The results obtained for the white spruce datasets 1 and 2 presented in this study are in bold. ^1^ We calculated the status number (*Ns*) based on the pedigree according to Eq. [2] when it was not reported in the original study. For Lenz et al., 2020a, 2020b, Laverdière et al. (2022), and this study, we used the available corrected pedigree following marker-based parental assignments.

^2^ We calculated the *PACC_BV_* as $PA_{BV}/\sqrt{{\hat{h}}_{ind}^{2}}$ using the ${\hat{h}}_{ind}^{2}$ obtained from GBLUP-A when it was not reported in the original study.

^3^ For Lenz et al., (2017), we reported the results obtained with the marker-based combined-site analyses.

^4^ Folding within families: trees were randomly split into 10 folds, making sure that each fold contained ~10% of the trees from each family.

When comparing the ability of ABLUP and GBLUP models to predict the breeding and genetic values of validation trees under the first CV scenario, we found no marked differences in *PA* between ABLUP-A and GBLUP-A, nor between ABLUP-AD and GBLUP-AD. The results were identical for *PACC* since we used the same heritability estimates for ABLUP and GBLUP. Other studies that calculated *PA* using similar CV schemes in full-sib trials (random folding or folding within families as in this study) found either no improvements (Lenz et al., 2017, 2020b; Chen et al., 2018, 2019; Pégard et al., 2020; Calleja-Rodriguez et al., 2021) or small improvements of *PA* using GBLUP versus ABLUP (+7% on average in Beaulieu et al., 2020). One study reported very large improvements (+55%) using GBLUP-A or GBLUP-AD over the corresponding ABLUP models with a corrected pedigree (Tan et al., 2018).

The absence of improvement of *PA* under CV1 in our study between GBLUP and ABLUP may be in part due to the pedigree correction that we performed using marker data, as ~10% of the trees were found to be misclassified. Pedigree corrections informed by markers in full-sib tree breeding populations with different percentage of errors (7% in Munoz et al., 2014; 78% in Tan et al., 2018; 15% in Pégard et al., 2020) markedly improved the predictive abilities/accuracies, sometimes to levels that were similar to GS models (Pégard et al., 2020). Yet, even with a corrected pedigree, GBLUP should describe more precisely the variation around expected relationships between individuals due to Mendelian sampling within families (VanRaden, 2008; Legarra et al., 2009; Beaulieu et al., 2022). When predictive ability or accuracy is calculated among-families as in this study for the CV1 and CV2 scenarios, it includes both the parent average component (family means) and the Mendelian sampling term, while within-family predictions only measure the prediction of the Mendelian sampling term (Werner et al., 2020). The lack of substantial increase of *PA* or *PACC* between GBLUP and ABLUP in this and some other studies may be due to the relatively small number of trees per family (~28) or to the expected smaller within- versus among-family genetic variances (Falconer and Mackay, 1996), which may reduce the contribution of any significant within-family predictive ability that would confer an advantage to GBLUP.

The within-family accuracy of genomic predictions in white spruce with the current effective and training population sizes, and marker density remains to be tested. Using a clonally replicated trial, Pégard et al. (2020) reported similar *PACC* between GBLUP and ABLUP using a random folding CV scenario (i.e., *PACC* calculated among families) and 7K SNPs, but were still able to show an advantage of GBLUP when ranking individuals within full-sib families. Other studies reported significantly positive within-family *PA* or *PACC* values, either from full-sib family trials without clonal replication (Resende et al., 2017; Ukrainetz and Mansfield, 2020), or with clonal replication (Walker et al., 2022). Thus, we expect that GS models would allow performing both among-family and within-family selections when phenotypes are not available, if there is high relatedness between the training and validation populations (Lauer et al., 2022).

### 4.5 Including dominance improved the predictive ability, but reduced the accuracy of models when predicting within trained families

Under CV1 with both datasets 1 and 2, the inclusion of dominance in the additive-dominance models increased the predictive ability of genetic values (breeding values + dominance deviations, *PA_GV_*) compared with the predictive ability of breeding values (*PA_BV_*) obtained from the additive models. This was observed only for growth traits because wood traits showed little dominance variance. For growth traits, the *PA* increased from the additive to the additive-dominance models by 6.4% and 8.2% on average for ABLUP and GBLUP, respectively. This increase was expected because the additive-dominance models could predict a larger portion of the phenotypic variation due to the inclusion of significant dominance genetic effects. However, this improvement in *PA* was rather small considering that the dominance variance accounted for about half of the genetic variance for growth traits and that the broad-sense heritabilities obtained from the additive-dominance models were ~55% higher on average than the narrow-sense heritabilities estimated by the additive models. The standardization of *PA* as $PA/\sqrt{h^{2}}$ ( ${\hat{h}}_{ind}^{2}$ for the additive models; ${\hat{H}}_{ind}^{2}$ for the additive-dominance models) resulted in a decrease of the estimated accuracies (*PACC*) from the additive to the additive-dominance models for all traits, but more so for growth traits (ABLUP: -10.1%; GBLUP: -8.5%). These results indicated that the additive-dominance models could predict with less accuracy the total genetic values compared with breeding values under CV1.

Previous studies in forest trees also reported increases in *PA* by including dominance in GBLUP models when considerable dominance variance was detected (0 to 21%; average 9%; **Table 6**), but the accuracy based on $PA/\sqrt{h^{2}}$ was not estimated. If we use the reported GBLUP heritabilities to calculate *PACC*, we find a decrease from the additive to the additive-dominance models in all cases, similar to this study (-1% to -29%; average -12.3%; **Table 6**). Thus, in this and previous studies, the *PA* of the additive-dominance models did not increase to the extent that would be expected based on the increase in broad-sense heritabilities. Tan et al. (2018) attributed these results to the large standard errors of the dominance variance estimates, the large effective population size, and the small number of individuals per family. In our study, the standard errors of the dominance variances for growth traits were rather small in the larger dataset 1 (ratio standard error/variance=0.23–0.33) and roughly equal to the standard errors of the additive variances. Similar results were found by de Almeida Filho et al. (2016; 2019). using both empirical and simulated data for a loblolly pine clonal population with a full-sib mating design. In simulations, these authors found that the prediction accuracy of dominance deviations was lower (0.24–0.26) than the accuracy of breeding values (0.55–0.61), even under high levels of dominance ( ${\hat{d}}_{ind}^{2}$ =0.20; de Almeida Filho et al., 2019). However, they still found an increase in *PACC* of total genetic values with the additive-dominance models when dominance was high ( ${\hat{d}}_{ind}^{2}$ >0.20). In our study, ${\hat{d}}_{ind}^{2}$ was below 0.20 for all traits under GBLUP. Indeed, accurately predicting dominance demands much more information since it relies on measurements of phenotypes in heterozygous individuals (Toro and Varona, 2010), and the use of a large training population of full-sibs, including markers with high minor allele frequency, could improve estimates (Denis and Bouvet, 2013; Ertl et al., 2014; Nishio and Satoh, 2014). We conclude that, although the *PA* of total genetic values was improved under the additive-dominance models and CV1, indicating that we can predict dominance deviations to some extent, the *PACC* of genetic values decreased due to a lower accuracy of dominance deviations compared with that of breeding values.

**Table 6:** Summary of studies that previously compared additive (A) and additive-dominance (AD) genomic selection models in tree species.

Study	Species	Model	Cross-validation scheme^4^	Trait	*PA_BV_* (A model)^5^	*PA_GV_* (AD model)^5^	% change	*PACC_BV_* (A model)	*PACC_GV_* (AD model)	% change
de Almeida Filho et al. (2016) ^1^	*Pinus taeda*	BRR^3^	Random folding (10-folds)	Height (6 yr.)	0.40 (0.04)	0.42 (0.03)	4.0	0.64	0.60	-6.1
Resende et al. (2017) ^1^	*Eucalyptus urophylla x E. grandis*	GBLUP	Random folding (10-folds)	Mean annual increment (3 yr.)	0.49	0.52	6.1	0.96	0.75	-21.9
Tan et al. (2018) ^1^	*Eucalyptus urophylla x E. grandis*	GBLUP	Random folding (10-folds)	Circumference at breast height (3 yr.)	0.16 (0.10)	0.18 (0.10)	12.5	0.43	0.42	-0.8
				Circumference at breast height (4 yr.)	0.27 (0.11)	0.30 (0.11)	11.1	0.60	0.55	-9.3
				Height (3 yr.)	0.26 (0.10)	0.30 (0.08)	15.4	0.57	0.49	-13.1
				Height (6 yr.)	0.32 (0.11)	0.36 (0.11)	12.5	0.68	0.65	-5.2
Chen et al. (2019) ^1^	*Picea abies*	GBLUP	MET site 1 to site 1 (10-folds)^4^	Height (17 yr.)	0.23 (0.04)	0.26 (0.03)	13.0	0.64	0.45	-29.0
			MET site 2 to site 2 (10-folds)^4^	Height (17 yr.)	0.25 (0.03)	0.27 (0.03)	8.0	0.59	0.50	-14.9
Beaulieu et al. (2020)	*Picea glauca*	GBLUP	Folding within families (10-folds)	Height (16 to 28 yr.)	0.25 (0.00)	0.27 (0.01)	8.0	0.51	0.47	-7.8
				DBH (16 to 28 yr.)	0.15 (0.01)	0.15 (0.01)	0.0	0.41	0.35	-14.6
				Volume (16 to 28 yr.)	0.14 (0.01)	0.15 (0.01)	7.1	0.38	0.33	-13.2
				Acoustic velocity (17 to 29 yr.)	0.40 (0.01)	0.42 (0.01)	5.0	0.63	0.57	-9.5
Thumma et al. (2022) ^1,2^	*Eucalyptus nitens*	GBLUP	Random folding (10-folds)	DBH (8.8 to 18.6 yr.)	0.29 (0.03)	0.35 (0.02)	21.0	0.73	0.62	-15.0

We only considered studies that calculated the predictive ability of breeding values (*PA_BV_*) and of genetic values (*PA_GV_*) (i.e., the correlation between the predicted values and the phenotypes) and only reported traits that showed considerable dominance variance under the genomic AD model.

^1^ For these studies, we calculated the prediction accuracy of breeding (*PACC_BV_*) and of genetic values (*PACC_GV_*) based on $PA_{BV}/\sqrt{{\hat{h}}_{ind}^{2}}$ and $PA_{GV}/\sqrt{{\hat{H}}_{ind}^{2}}$ , respectively, using the reported GBLUP heritability estimates.

^2^ The *PA_BV_* and *PA_GV_* results of Thumma et al. (2022; **Figure 1)** were provided by B. R. Thumma (personal communication). We used the results obtained with the 868 genotyped progeny trees. For the GBLUP additive model, we used the results obtained from the identity-by-state model.

^3^ BRR: Bayesian ridge regression.

^4^ Random folding: trees were randomly split into 10 folds; folding within families: trees were randomly split into 10 folds, making sure that each fold contained ~10% of the trees from each family; MET: We used the reported multi-environment trial analysis of Chen et al. (2019).

^5^ The reported standard errors are in parentheses.

The CV1 scenario simulated the prediction of additional genotypes from the same families as those included in the training population, which is within the current generation, and has direct applications in forest tree breeding. For example, GS models could be beneficial for the selection of somatic embryogenesis (SE) lines, for which the pedigree-based methods do not allow within-family ranking. As such, thousands of cryo-conserved SE lines can be genotyped to predict their genetic values. The best individuals, both across and within families, can then be deployed *via* vegetative propagation techniques such as rooted cuttings or somatic embryogenesis (SE), which is highly amenable for a large diversity of clonal lines in spruce species (Park et al., 2016). Such clonal deployment of SE lines is already undergoing for reforestation purposes in the provinces of New Brunswick and Québec in Canada (Perron et al., 2018). This strategy has the potential to yield larger genetic gains per unit of time because 1) it can exploit more of the genetic variance, that is the additive and dominance variances, as shown by the higher broad-sense heritabilities and increased predictive abilities of genetic values (*PA_GV_*) for growth traits in this study; 2) the selection intensity could be largely increased by genotyping more individuals; and 3) it dramatically reduces the breeding cycle length because no field testing is required for the SE lines. Thus, we expect that the predictions of total genetic values by GS models, although less accurate than those of breeding values, should still allow increasing genetic gains.

### 4.6 GBLUP showed superior predictive ability compared to ABLUP when predicting in unphenotyped families by utilizing dominance variance

We found different trends under the second cross-validation scenario (CV2), in which the training and validation datasets included different families, thus simulating the prediction of breeding and genetic values for new unphenotyped full-sib families. However, one must keep in mind that those predicted families were half-sib related to a few families in the training dataset. While there were no differences in predictive ability of breeding values (*PA_BV_*) between the additive ABLUP-A and GBLUP-A models, the results were different for the additive-dominance models. We found that the predictive ability of breeding (*PA_BV_*) and of genetic values (*PA_GV_*) for growth traits were substantially increased by 22% and 53% on average, respectively, for GBLUP-AD versus ABLUP-AD. For wood traits, there was a slight advantage of GBLUP-AD for wood density (~5–10%), which presented small dominance effects, but no advantage was observed for acoustic velocity.

This marked advantage of GBLUP-AD over ABLUP-AD for growth traits has two explanations. On one hand, fitting dominance under ABLUP-AD decreased *PA* and *PACC* of both breeding and genetic values for most traits, compared with ABLUP-A. It should also be noted that *PA_BV_* was always equal to *PA_GV_* under ABLUP-AD. This is because the predicted dominance deviations were null for all individuals under ABLUP-AD in the absence of phenotypic information for these new families, leaving only the predicted breeding values for the calculation of *PA_GV_*. Thus, ABLUP cannot capitalize on dominance deviations in the prediction of new families. On the other hand, GBLUP-AD could use genomic information in the dominant genomic relationship matrix to estimate dominance deviations in unphenotyped families. Indeed, fitting dominance under GBLUP-AD slightly increased the predictive ability of breeding values (*PA_BV_*) by 5%, but substantially increased the predictive ability of genetic values (*PA_GV_*) by 24%, on average, compared with GBLUP-A. This increase in *PA_GV_* led to an increase in *PACC_GV_* of 10% from GBLUP-A to GBLUP-AD, on average. The trait that showed the largest improvement was DBH in dataset 2 (+54% in *PA_GV_* and +27% in *PACC_GV_*), which was also the trait that had the highest proportion of genetic variation attributed to dominance under GBLUP-AD (82%; **Table S8**). Thus, the large differences in predictive ability of genetic values that we found between GBLUP-AD and ABLUP-AD could be mostly attributed to the ability of GBLUP to predict dominance deviations in unphenotyped families, and the inability of ABLUP to do so. Moreover, we found that GBLUP-AD was superior to ABLUP-AD in predicting both breeding and genetic values for new families, likely because of a better estimation of additive and dominance variances.

The findings that GBLUP-AD can predict dominance for unphenotyped families, but not ABLUP, are novel, and, to our knowledge, have never been reported for full-sib mating designs in tree species. Resende et al. (2017) tested a similar CV scenario using a full-sib trial and found an increased predictive ability of genetic values (+25%) for unphenotyped families from GBLUP-A to GBLUP-AD for mean annual increment, similarly to this study, but they did not compare with ABLUP. Our results are encouraging from a tree breeding perspective. Although the predictive abilities and accuracies decreased under the CV2 scenario with half-sib relatedness between training and validation datasets, compared with the CV1 scenario with full-sib relatedness, as previously noted (e.g., Beaulieu et al., 2014b; de Almeida Filho et al., 2019; Lauer et al., 2022), the accuracies of genetic values obtained using GBLUP-AD were still acceptable for growth traits (0.45–0.53 in dataset 1) and high for wood traits (0.68–0.69 in dataset 1).

**Figure 5:**
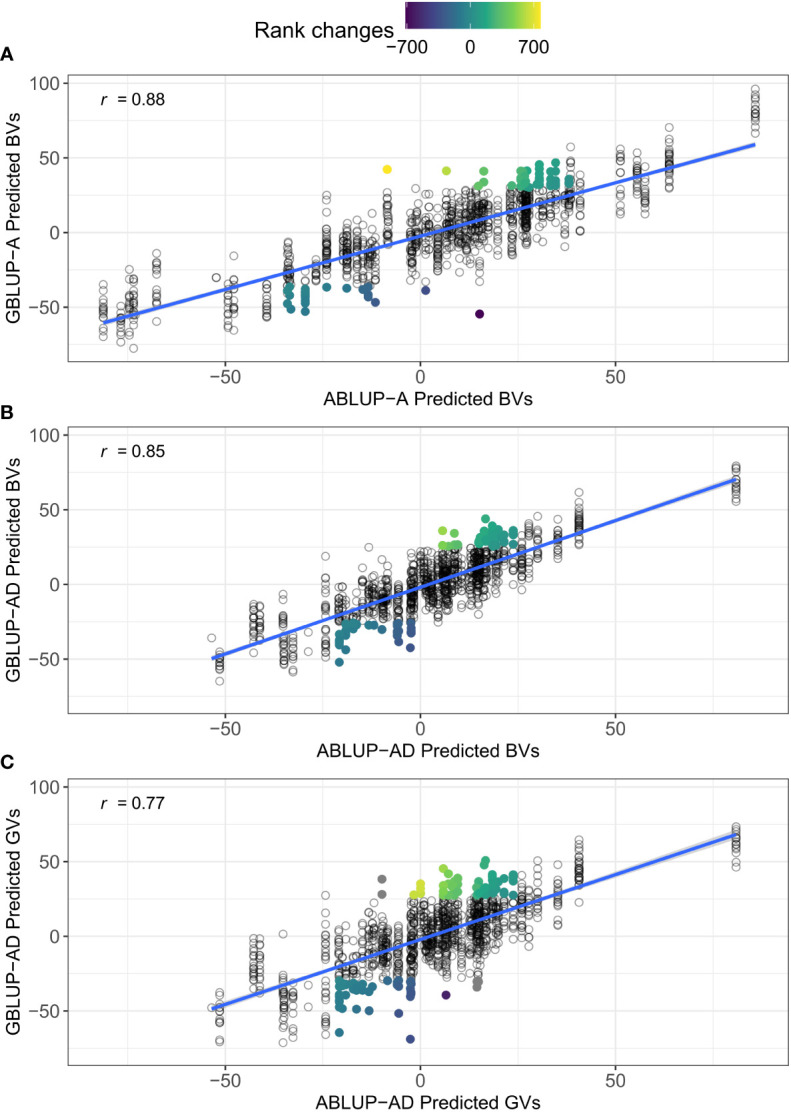
Comparisons between the predicted breeding (BVs) and genetic values (GVs) between ABLUP and GBLUP. An example is shown for height growth on site Asselin (dataset 1) and the cross-validation scenario CV2, which simulated the prediction of new unphenotyped families. **(A)** correlation between the predicted BVs from ABLUP-A and GBLUP-A; **(B)** correlation between the predicted BVs from ABLUP-AD and GBLUP-AD; and **(C)** correlation between the predicted GVs from ABLUP-AD and GBLUP-AD. The Pearson correlation coefficient (*r*) is given for each panel. In each panel, the top and bottom 10% individuals that would be selected or culled using only GBLUP, but not using ABLUP, are highlighted. The color gradient for highlighted individuals represents the rank changes between ABLUP and GBLUP selections. The overlap between ABLUP and GBLUP top and bottom 10% trees was 73% in **(A)**, 64% in **(B)**, and 55% in **(C)**. Note that under ABLUP, only the mid-parent breeding values can be assigned in the absence of phenotypes. Also, for ABLUP-AD and CV2, the predicted dominance deviations were null for all individuals, such that the predicted BVs (x-axis in **B**) were equal to the predicted GVs (x-axis in **C**).

The CV2 scenario is of most interest to breeders because it is closer to producing new crosses for the next generation cycle. One promising application of GS would be to select the best individuals at a very young age based on their genomic predictions, thus skipping field testing and greatly reducing the length of the breeding cycle. Here, we highlight another potentially fruitful application of GS in tree breeding for the current generation, that is mating allocation (Toro and Varona, 2010). The findings that GBLUP-AD increases the predictive ability/accuracy of genetic values for new families in CV2 suggest that we could identify crosses that would produce offspring with the highest total genetic values. Indeed, it is straightforward to predict the genetic merit of offspring of a future mating by modeling additive and dominance effects using genomic data (Toro and Varona, 2010). The resulting embryos or seedlings from these new crosses could be propagated as elite families or undergo a further forward selection step by genotyping and predicting their individual genetic value before vegetative propagation and clonal deployment. As an example, **Figure 5** shows that very different individual trees would be selected or culled when the predictions for height growth were based on GBLUP in this study, with only 64% and 55% overlap between ABLUP-AD and GBLUP-AD for the top and bottom 10% individuals based on breeding (panel B) or genetic values (panel C), respectively.

Mate allocation using GS models was found to increase the selection response in animal breeding (Toro and Varona, 2010; Ertl et al., 2014; Aliloo et al., 2017). This only applies to the production population in the current generation because only additive effects, not dominance, are transmitted to the next generation. In dataset 1, there are *n*(*n*−1)/2=1,891 possible ways to combine the 62 parents of the four breeding groups, including crosses between breeding groups, but only 90 crosses were tested in the present study. Although the accuracy was smaller when predicting new families (-25% on average for CV2 versus CV1 in dataset 1), this could be compensated by the large increase in selection intensity from predicting additional parent-pairs and thus, ultimately, could lead to higher genetic gains.

At the operational level, this strategy would be relatively quick to implement because the parents are already sexually mature and good phenotypic data is available for their tested progeny, also allowing the measurement of new relevant traits, for instance in relation to adaptation to climate (Laverdière et al., 2022). Another major advantage is that it would allow performing the induction of somatic embryogenesis only for the crosses with high predicted genetic values given that not all embryos succeed forward this first step (~60% in white spruce; Laurence Tremblay, Ministère des Ressources naturelles et des Forêts du Québec, personal communication). Moreover, this would allow obtaining predicted genetic values even for crosses between breeding groups, which may have higher genetic potential due to the combination of different genetic backgrounds and higher heterozygosity, although the realized prediction accuracy may be smaller because only crosses within breeding groups were tested in this study. Overall, we find that the inclusion of dominance in GS models is promising for the genomic evaluation of new full-sib crosses for mating allocation within the same generation, which in turn should allow to substantially increase genetic gains.

### 4.7 Large sample sizes were required for accurate estimation of genetic parameters and genetic value predictions

We used resampling to evaluate the ability of the ABLUP-AD and GBLUP-AD models to estimate genetic parameters and predict genetic values for different levels of number of families (12–72) or trees per family (6–26) sampled in dataset 1. For our breeding population, we found that a large sample size of 60-72 families and the maximum of 26 trees per family was required for accurate estimation of broad-sense heritabilities ( ${\hat{H}}_{ind}^{2}$ ), as measured by the standard deviations of estimates. Fewer trees per family (12-20) were required for accurate prediction of genetic values, as measured by *PA_GV_* or *PACC_GV_* under CV2. Evaluating the minimum number of families needed for accurate predictions of genetic values was more difficult because, as the number of families sampled decreased, some families in the validation datasets became unrelated with the training population in CV2 (**Table S11**). Hence, the reduction in relatedness between training and validation datasets explained the larger decrease of *PA_GV_* and *PACC_GV_* with decreasing the number of families versus decreasing the number of trees per family. Perron et al. (2013) used similar resampling approaches in open-pollinated progeny trials of black spruce and tamarack to determine that a minimum of 75 families and 12 trees per family per site was needed for accurate estimation of genetic parameters using pedigree-based models. Similarly, Chen et al. (2018) found that a large number of families (up to 120) or trees per family (6–18) were required for accurate prediction of breeding values using GS models in a Norway spruce full-sib trial. In our study, we did not determine the best sampling strategy for a given number of sampled trees, that is sampling more families or more trees per family. Addressing that question would have required a larger sample size, and the results would be specific to the breeding population, crossing scheme, and experimental design employed.

An important finding of our study is that GBLUP-AD estimated genetic parameters and predicted genetic values with greater accuracy than ABLUP-AD at all sample sizes tested, especially for growth traits. For these traits, smaller sample sizes were needed in GBLUP-AD to obtain similar prediction abilities of genetic values compared to ABLUP-AD under CV2. Similarly, Walker et al. (2022) showed that GBLUP needed a smaller number of ramets per clone to obtain similar accuracies compared to ABLUP. We conclude that GS programs should focus on genotyping a sufficient number of trees in the phenotyped training population to obtain accurate genetic parameter estimates and genetic value predictions.

### 4.8 Conclusion

Using two large full-sib datasets, we evaluated the inclusion of dominance in pedigree-based ABLUP and genomic-based GBLUP models for wood and growth traits. Wood traits were found to be optimal candidates for tree breeding efficiency, as they presented higher narrow-sense heritabilities and lower GxE than growth traits. High accuracies of GS models were even maintained when predicting for new unphenotyped families, which were half-sib related to a few families in the training dataset, a scenario that is closer to the production of the next generation.

Predictions for growth traits will highly benefit from application of GS instead of using pedigree-based methods. GBLUP led to more realistic estimates of genetic variances and better partitioning of additive and non-additive variances, thus allowing to better plan the methods of selection for breeding purposes and the propagation of reforestation material. For growth traits, the use of GBLUP-AD led to higher predictive abilities for new families (CV2) than ABLUP-AD, mainly due to the ability of GBLUP-AD to predict dominance. The predictive ability of breeding values was slightly improved under GBLUP-AD for new families, compared with ABLUP-AD. Dominance was generally predicted with smaller accuracy than breeding values in GBLUP-AD (CV1). Still, it was sufficiently accurate to substantially increase predictive abilities and accuracies for unphenotyped families (CV2), and to outperform ABLUP-AD. It can be concluded that the dominance term should always be included into models when significant dominance variance is expected, such as for growth traits in this study.

By subsampling various sets of families and trees per family, we found that GBLUP produced better estimates of genetic variances and higher predictive abilities than ABLUP for all subsamples, especially for growth traits. Results also highlight the need to rely on sufficiently large sample sizes to obtain accurate estimates of genetic parameters and predictions of breeding and genetic values.

The ability of GBLUP to predict for new unphenotyped families also provided useful insights for next-generation prediction accuracies. Nonetheless, there is a need for next-generation studies in white spruce and other important tree breeding species. Meanwhile, predicting for new crosses may be used to perform mating allocation and maximize the total genetic values for elite family or clonal propagation in the current generation.

## Data availability statement

The datasets presented in this study can be found in online repositories. The names of the repository/repositories and accession number(s) can be found below: https://treesource.rncan.gc.ca/en.

## Author contributions

SN, JBe, JBo, and PRNL conceived the study. SN and PRNL wrote the manuscript. JBo, PRNL, SG, JBe, and MP edited the manuscript. SN and SG performed the statistical analyses. JBe, JBo, PRNL, and MP set up and maintained the field trial. JBo, JBe, and PRNL obtained funding to support genotyping and phenotyping. All authors contributed to the article and approved the submitted version.
