# Supplementary material for: Increasing genomic prediction accuracy for unphenotyped full-sib families by modeling additive and dominance effects with large datasets in white spruce
Source: Front Plant Sci. 2023 Mar 22;14:1137834. doi: 10.3389/fpls.2023.1137834 (PMC10073444; doi:10.3389/fpls.2023.1137834)
Supplement: Supplementary file 1 [file DataSheet_1.pdf]

SUPPLEMENTARY TABLES:

Table S1. For the complete **dataset 1**, variance components estimated using the **full additive-dominance GBLUP-AD models** (Eq. [3] in the manuscript) that included the effects of the breeding group ($\boldsymbol{bg}$) and site-by-BG interaction ($\boldsymbol{s}:\boldsymbol{bg}$).

Variance components[1]	AV_{16}	WD_{16}	HT_{16}	DBH_{16}	VOL_{16}
$\hat{\sigma}^2_{bg}$	0.00 (0.00)	111.98 (110.12)	0.00 (NA)†	14.94 (17.53)	2.92 (5.74)
$\hat{\sigma}^2_{s:bg}$	0.00 (0.00)	0.00 (NA)†	84.73 (127.51)	3.82 (5.43)	2.18 (3.15)
$\hat{\sigma}^2_{b\_ASS}$	0.04 (0.02)	68.93 (37.41)	2319.13 (1194.22)	29.41 (16.29)	18.76 (10.08)
$\hat{\sigma}^2_{b\_SCA}$	0.01 (0.00)	132.70 (68.26)	1730.16 (883.57)	55.01 (27.84)	39.92 (20.16)
$\hat{\sigma}^2_{p\_ASS}$	0.03 (0.00)	111.18 (28.24)	1511.05 (296.83)	65.57 (14.58)	26.25 (6.08)
$\hat{\sigma}^2_{p\_SCA}$	0.01 (0.00)	80.16 (22.51)	1725.20 (335.36)	71.99 (11.95)	44.88 (8.22)
$\hat{r}_{Ba}$	0.99 (NA)†	0.99 (NA)†	0.67 (0.14)	0.66 (0.24)	0.68 (0.17)
$\hat{\sigma}^2_{a\_ASS}$	0.04 (0.01)	198.62 (36.44)	1489.86 (366.11)	28.37 (13.20)	19.21 (6.34)
$\hat{\sigma}^2_{a\_SCA}$	0.04 (0.01)	286.26 (42.62)	1996.59 (487.67)	45.58 (13.91)	37.62 (10.49)
$\hat{r}_{Bd}$	0.99 (NA)†	0.66 (0.27)	0.99 (NA)†	0.70 (0.29)	0.99 (NA)†
$\hat{\sigma}^2_{d\_ASS}$	0.01 (0.01)	88.54 (32.04)	657.67 (270.51)	50.61 (17.04)	16.07 (6.01)
$\hat{\sigma}^2_{d\_SCA}$	0.01 (0.00)	74.18 (32.02)	1507.07 (429.32)	38.85 (14.26)	25.46 (9.08)
$\hat{\sigma}^2_{e\_ASS}$	0.06 (0.01)	364.26 (36.54)	3762.69 (348.30)	201.26 (17.96)	90.57 (7.49)
$\hat{\sigma}^2_{e\_SCA}$	0.06 (0.01)	302.92 (33.50)	4067.99 (428.94)	149.35 (14.68)	110.04 (10.18)
AIC of model in Eq. [3]	-2943	**17870**	24345	**16493**	15071
AIC of model in Eq. [7]	**-2946**	17874	**24342**	16496	**15069**
BIC of model in Eq. [3]	-2862	17951	24427	16574	15152
BIC of model in Eq. [7]	**-2876**	**17943**	**24412**	**16565**	**15139**

The smallest AIC and BIC between the full model in Eq. [3] and the final model in Eq. [7] without the $\boldsymbol{bg}$ and $\boldsymbol{s}:\boldsymbol{bg}$ effects for each trait are in bold. See Table 2 for a description of traits.

[1] $\hat{\sigma}^2_{bg}$ is the BG variance; $\hat{\sigma}^2_{s:bg}$ is the site-by-BG interaction variance; $\hat{\sigma}^2_{b\_ASS}$ and $\hat{\sigma}^2_{b\_SCA}$ are the block variances for site Asselin (ASS) and St. Casimir (SCA) respectively; $\hat{\sigma}^2_{p\_ASS}$ and $\hat{\sigma}^2_{p\_SCA}$ are the plot variances for site ASS and SCA respectively; $\hat{\sigma}^2_{a\_ASS}$ and $\hat{\sigma}^2_{a\_SCA}$ are the additive genetic variances for site ASS and SCA respectively; $\hat{\sigma}^2_{d\_ASS}$ and $\hat{\sigma}^2_{d\_SCA}$ are the dominance genetic variances for site ASS and SCA respectively; $\hat{r}_{Ba}$ and $\hat{r}_{Bd}$ are the correlation of additive and dominance effects between sites, respectively; $\hat{\sigma}^2_{e\_ASS}$ and $\hat{\sigma}^2_{e\_SCA}$ are the residual error variances for site ASS and SCA respectively. All models converged.

† These terms were at boundary (very close to 0 or 1) after convergence of the model.

Table S2. For the complete **dataset 2**, variance components estimated using the **full additive-dominance GBLUP-AD models** (Eq. [3] in the manuscript) that included the effects of the breeding group ($\boldsymbol{bg}$) and site-by-BG interaction ($\boldsymbol{s}:\boldsymbol{bg}$).

Variance components[1]	AV_{13}	WD_{13}	HT_{13}	DBH_{13}	VOL_{13}
$\hat{\sigma}^2_{bg}$	0.00 (NA)†	0.00 (NA)†	0.00 (NA)†	0.00 (NA)†	0.00 (NA)†
$\hat{\sigma}^2_{s:bg}$	0.00 (NA)†	0.00 (NA)†	18.96 (101.53)	0.00 (NA)†	0.00 (NA)†
$\hat{\sigma}^2_{b\_ASS}$	0.02 (0.01)	60.57 (40.10)	1208.00 (682.07)	28.57 (17.58)	2.92 (1.80)
$\hat{\sigma}^2_{b\_SCA}$	0.01 (0.01)	25.70 (19.46)	681.33 (462.06)	14.73 (11.05)	2.78 (1.98)
$\hat{\sigma}^2_{p\_ASS}$	0.02 (0.01)	73.91 (31.07)	691.35 (220.73)	30.38 (10.12)	3.12 (1.01)
$\hat{\sigma}^2_{p\_SCA}$	0.02 (0.00)	55.91 (26.87)	467.75 (320.09)	16.09 (9.73)	3.11 (1.59)
$\hat{r}_{Ba}$	0.99 (0.06)	0.99 (NA)†	0.43 (0.23)	0.20 (0.28)	0.30 (0.25)
$\hat{\sigma}^2_{a\_ASS}$	0.05 (0.01)	296.90 (62.18)	806.30 (298.15)	28.10 (12.28)	3.27 (1.28)
$\hat{\sigma}^2_{a\_SCA}$	0.03 (0.01)	239.73 (53.22)	1807.99 (625.01)	41.53 (16.30)	7.23 (2.78)
$\hat{r}_{Bd}$	0.99 (NA)†	0.67 (0.50)	0.79 (0.41)	0.99 (NA)†	0.99 (NA)†
$\hat{\sigma}^2_{d\_ASS}$	0.01 (0.01)	94.85 (54.19)	623.48 (341.31)	28.87 (13.90)	2.57 (1.31)
$\hat{\sigma}^2_{d\_SCA}$	0.01 (0.01)	46.95 (46.59)	1442.09 (599.72)	35.76 (15.90)	6.10 (2.68)
$\hat{\sigma}^2_{e\_ASS}$	0.06 (0.01)	336.15 (52.38)	2957.01 (328.59)	144.25 (14.42)	14.52 (1.42)
$\hat{\sigma}^2_{e\_SCA}$	0.05 (0.01)	435.12 (50.51)	5217.00 (589.64)	170.13 (17.15)	26.25 (2.77)
AIC of model in Eq. [3]	-1931	11095	15581	10338	7055
AIC of model in Eq. [7]	**-1935**	**11091**	**15577**	**10334**	**7051**
BIC of model in Eq. [3]	-1857	11169	15656	10413	7130
BIC of model in Eq. [7]	**-1871**	**11155**	**15641**	**10398**	**7115**

The smallest AIC and BIC between the full model in Eq. [3] and the final model in Eq. [7] without the $\boldsymbol{bg}$ and $\boldsymbol{s}:\boldsymbol{bg}$ effects for each trait are in bold. See Table 2 for a description of traits.

[1] Variance components are described in Table S1. All models converged.

† These terms were at boundary (very close to 0 or 1) after convergence of the model.

Table S3. Equations used to estimate the genetic parameters within each site from the final additive and additive-dominance models (Eq. [7] in the manuscript). The equations are given using the variance parameters obtained for site Asselin (ASS). The same equations were used for site St. Casimir (SCA), but using the variances obtained for site SCA.

Genetic parameter	Equation
**Additive model**	
Individual narrow-sense heritability	$\hat{h}^2_{ind\_ASS} = \hat{\sigma}^2_{a\_ASS} / (\hat{\sigma}^2_{p\_ASS} + \hat{\sigma}^2_{a\_ASS} + \hat{\sigma}^2_{e\_ASS})$
**Additive-dominance model**	
Individual narrow-sense heritability	$\hat{h}^2_{ind\_ASS} = \hat{\sigma}^2_{a\_ASS} / (\hat{\sigma}^2_{p\_ASS} + \hat{\sigma}^2_{a\_ASS} + \hat{\sigma}^2_{d\_ASS} + \hat{\sigma}^2_{e\_ASS})$
Individual dominance ratio	$\hat{d}^2_{ind\_ASS} = \hat{\sigma}^2_{d\_ASS} / (\hat{\sigma}^2_{p\_ASS} + \hat{\sigma}^2_{a\_ASS} + \hat{\sigma}^2_{d\_ASS} + \hat{\sigma}^2_{e\_ASS})$
Individual broad-sense heritability	$\hat{H}^2_{ind\_ASS} = (\hat{\sigma}^2_{a\_ASS} + \hat{\sigma}^2_{d\_ASS}) / (\hat{\sigma}^2_{p\_ASS} + \hat{\sigma}^2_{a\_ASS} + \hat{\sigma}^2_{d\_ASS} + \hat{\sigma}^2_{e\_ASS})$

$\sigma^2_{p\_ASS}$ is the plot variances for site ASS; $\sigma^2_{a\_ASS}$ is the additive genetic variances for site ASS; $\sigma^2_{d\_ASS}$ is the dominance genetic variances for site ASS; $\sigma^2_{e\_ASS}$ is the residual error variances for site ASS.

Table S4. For the complete **dataset 1**, variance components and within-site heritabilities estimated using the **final additive ABLUP-A and GBLUP-A models** (Eq. [7] in the manuscript[1]).

Variance components[2]	AV_{16}	WD_{16}	HT_{16}	DBH_{16}	VOL_{16}
**ABLUP-A**					
$\hat{\sigma}^2_{b\_ASS}$	0.04 (0.02)	68.35 (37.32)	2339.48 (1204.83)	28.58 (15.96)	18.67 (10.05)
$\hat{\sigma}^2_{b\_SCA}$	0.01 (0.00)	131.33 (67.72)	1871.94 (961.01)	57.10 (29.07)	41.86 (21.26)
$\hat{\sigma}^2_{p\_ASS}$	0.03 (0.01)	135.47 (30.03)	1566.33 (298.70)	79.34 (15.07)	29.72 (6.20)
$\hat{\sigma}^2_{p\_SCA}$	0.01 (0.00)	90.08 (23.15)	2034.02 (353.64)	77.85 (12.17)	48.99 (8.43)
$\hat{r}_{Ba}$	0.98 (0.03)	0.99 (0.02)	0.78 (0.09)	0.84 (0.09)	0.85 (0.08)
$\hat{\sigma}^2_{a\_ASS}$	0.07 (0.02)	496.88 (110.77)	3103.23 (811.36)	108.28 (30.43)	52.24 (14.19)
$\hat{\sigma}^2_{a\_SCA}$	0.07 (0.02)	675.55 (143.41)	4533.06 (1183.36)	141.60 (36.37)	97.33 (25.20)
$\hat{\sigma}^2_{e\_ASS}$	0.04 (0.01)	255.48 (63.33)	3312.24 (496.45)	191.15 (21.03)	82.86 (9.50)
$\hat{\sigma}^2_{e\_SCA}$	0.05 (0.01)	141.50 (76.47)	3757.52 (677.50)	127.33 (21.20)	96.90 (14.91)
$\hat{h}^2_{ind\_ASS}$	0.51 (0.09)	0.56 (0.09)	0.39 (0.08)	0.29 (0.07)	0.32 (0.07)
$\hat{h}^2_{ind\_SCA}$	0.57 (0.09)	0.74 (0.10)	0.44 (0.09)	0.41 (0.09)	0.40 (0.09)
**GBLUP-A**					
$\hat{\sigma}^2_{b\_ASS}$	0.04 (0.02)	67.31 (36.73)	2312.61 (1192.57)	28.17 (15.81)	18.46 (9.97)
$\hat{\sigma}^2_{b\_SCA}$	0.01 (0.00)	138.29 (71.30)	1938.00 (993.49)	60.00 (30.56)	43.32 (22.00)
$\hat{\sigma}^2_{p\_ASS}$	0.03 (0.01)	127.29 (29.13)	1715.43 (306.87)	84.64 (15.39)	32.17 (6.35)
$\hat{\sigma}^2_{p\_SCA}$	0.01 (0.00)	92.44 (22.98)	2158.39 (355.94)	82.86 (12.40)	51.48 (8.52)
$\hat{r}_{Ba}$	0.99 (0.04)	0.99 (0.04)	0.73 (0.11)	0.73 (0.14)	0.77 (0.12)
$\hat{\sigma}^2_{a\_ASS}$	0.04 (0.01)	249.26 (36.96)	1709.42 (364.59)	49.54 (13.43)	25.94 (6.36)
$\hat{\sigma}^2_{a\_SCA}$	0.04 (0.01)	332.14 (42.32)	2578.27 (496.64)	67.23 (14.28)	50.55 (10.57)
$\hat{\sigma}^2_{e\_ASS}$	0.07 (0.01)	412.87 (32.21)	4124.89 (319.19)	225.96 (15.67)	98.40 (6.87)
$\hat{\sigma}^2_{e\_SCA}$	0.07 (0.00)	343.09 (28.19)	4851.14 (369.49)	168.34 (12.20)	123.09 (8.91)
$\hat{h}^2_{ind\_ASS}$	0.29 (0.04)	0.32 (0.04)	0.23 (0.04)	0.14 (0.04)	0.17 (0.04)
$\hat{h}^2_{ind\_SCA}$	0.35 (0.04)	0.43 (0.04)	0.27 (0.04)	0.21 (0.04)	0.22 (0.04)

See Table 2 for a description of traits.

[1] The additive model fitted is described in Eq. [7], by excluding the dominance term $\boldsymbol{d}(\boldsymbol{s})$. All models converged.

[2] Variance components are described in Table S1. Within-site heritability estimates were calculated using the equations described in Table S3.

Table S5. For the complete **dataset 1**, variance components and within-site heritabilities estimated using the **final additive-dominance ABLUP-AD and GBLUP-AD models** (Eq. [7] in the manuscript[1]).

Variance components[1]	AV_{16}	WD_{16}	HT_{16}	DBH_{16}	VOL_{16}
**ABLUP-AD**					
$\hat{\sigma}^2_{b\_ASS}$	0.04 (0.02)	69.18 (37.6)	2371.79 (1219.86)	29.22 (16.16)	19.03 (10.19)
$\hat{\sigma}^2_{b\_SCA}$	0.01 (0.00)	123.04 (63.09)	1709.34 (875.78)	53.10 (26.90)	39.07 (19.76)
$\hat{\sigma}^2_{p\_ASS}$	0.03 (0.01)	117.73 (29.56)	1428.30 (293.50)	63.15 (14.41)	24.56 (5.97)
$\hat{\sigma}^2_{p\_SCA}$	0.01 (0.00)	70.33 (22.48)	1700.74 (338.50)	65.46 (11.76)	40.70 (8.10)
$\hat{r}_{Ba}$	0.98 (0.03)	0.99 (NA)†	0.62 (0.19)	0.85 (0.18)	0.83 (0.17)
$\hat{\sigma}^2_{a\_ASS}$	0.07 (0.02)	415.12 (113.08)	2074.65 (791.48)	56.06 (31.75)	28.53 (14.43)
$\hat{\sigma}^2_{a\_SCA}$	0.07 (0.02)	584.80 (145.97)	2372.58 (1062.44)	102.62 (36.84)	63.06 (24.69)
$\hat{r}_{Bd}$	0.99 (NA)†	0.89 (0.17)	0.95 (0.22)	0.78 (0.21)	0.79 (0.21)
$\hat{\sigma}^2_{d\_ASS}$	0.01 (0.01)	199.84 (100.82)	2004.02 (1052.25)	153.81 (60.13)	59.08 (24.80)
$\hat{\sigma}^2_{d\_SCA}$	0.01 (0.01)	214.16 (100.20)	4380.79 (1704.88)	115.99 (50.63)	86.62 (36.35)
$\hat{\sigma}^2_{e\_ASS}$	0.04 (0.01)	145.62 (86.10)	2291.92 (764.25)	101.76 (41.86)	49.85 (17.31)
$\hat{\sigma}^2_{e\_SCA}$	0.04 (0.01)	25.72 (94.75)	1534.11 (1171.66)	60.22 (36.75)	48.99 (25.86)
$\hat{h}^2_{ind\_ASS}$	0.49 (0.10)	0.47 (0.10)	0.27 (0.09)	0.15 (0.08)	0.18 (0.09)
$\hat{h}^2_{ind\_SCA}$	0.55 (0.10)	0.65 (0.12)	0.24 (0.10)	0.30 (0.10)	0.26 (0.09)
$\hat{d}^2_{ind\_ASS}$	0.08 (0.08)	0.23 (0.11)	0.26 (0.13)	0.41 (0.16)	0.36 (0.15)
$\hat{d}^2_{ind\_SCA}$	0.05 (0.07)	0.24 (0.11)	0.44 (0.17)	0.34 (0.14)	0.36 (0.15)
$\hat{H}^2_{ind\_ASS}$	0.57 (0.10)	0.70 (0.11)	0.52 (0.11)	0.56 (0.12)	0.54 (0.12)
$\hat{H}^2_{ind\_SCA}$	0.60 (0.10)	0.89 (0.11)	0.68 (0.13)	0.63 (0.12)	0.63 (0.12)

Table S5 [Continued]. For the complete **dataset 1**, variance components and within-site heritabilities estimated using the **final additive-dominance ABLUP-AD and GBLUP-AD models** (Eq. [7] in the manuscript[1]).

Variance components[1]	AV_{16}	WD_{16}	HT_{16}	DBH_{16}	VOL_{16}
**GBLUP-AD**					
$\hat{\sigma}^2_{b\_ASS}$	0.04 (0.02)	69.23 (37.56)	2313.60 (1191.47)	29.08 (16.13)	18.62 (10.01)
$\hat{\sigma}^2_{b\_SCA}$	0.01 (0.00)	133.34 (68.57)	1731.68 (884.29)	54.99 (27.83)	39.94 (20.17)
$\hat{\sigma}^2_{p\_ASS}$	0.03 (0.00)	108.76 (28.21)	1511.98 (296.95)	65.46 (14.61)	26.38 (6.11)
$\hat{\sigma}^2_{p\_SCA}$	0.01 (0.00)	79.12 (22.53)	1715.26 (335.11)	71.19 (11.94)	44.47 (8.23)
$\hat{r}_{Ba}$	0.99 (NA)†	0.99 (NA)†	0.63 (0.13)	0.70 (0.20)	0.67 (0.17)
$\hat{\sigma}^2_{a\_ASS}$	0.04 (0.01)	219.97 (35.74)	1504.51 (365.78)	33.35 (12.94)	20.32 (6.36)
$\hat{\sigma}^2_{a\_SCA}$	0.04 (0.01)	303.83 (42.29)	2077.02 (485.27)	55.96 (14.08)	41.95 (10.45)
$\hat{r}_{Bd}$	0.99 (NA)†	0.67 (0.27)	0.99 (NA)†	0.66 (0.28)	0.98 (0.38)
$\hat{\sigma}^2_{d\_ASS}$	0.01 (0.01)	87.34 (31.98)	660.14 (270.90)	51.31 (17.07)	16.12 (6.87)
$\hat{\sigma}^2_{d\_SCA}$	0.01 (0.00)	73.40 (32.03)	1518.70 (430.40)	41.05 (14.50)	26.02 (10.24)
$\hat{\sigma}^2_{e\_ASS}$	0.06 (0.01)	361.46 (36.61)	3754.15 (348.23)	199.53 (17.97)	90.17 (7.74)
$\hat{\sigma}^2_{e\_SCA}$	0.06 (0.01)	300.36 (33.42)	4033.30 (427.92)	144.11 (14.62)	108.15 (10.57)
$\hat{h}^2_{ind\_ASS}$	0.27 (0.04)	0.28 (0.04)	0.20 (0.04)	0.10 (0.04)	0.13 (0.04)
$\hat{h}^2_{ind\_SCA}$	0.34 (0.04)	0.40 (0.04)	0.22 (0.05)	0.18 (0.04)	0.19 (0.04)
$\hat{d}^2_{ind\_ASS}$	0.05 (0.04)	0.11 (0.04)	0.09 (0.04)	0.15 (0.05)	0.11 (0.04)
$\hat{d}^2_{ind\_SCA}$	0.06 (0.04)	0.10 (0.04)	0.16 (0.05)	0.13 (0.05)	0.12 (0.05)
$\hat{H}^2_{ind\_ASS}$	0.32 (0.05)	0.40 (0.05)	0.29 (0.05)	0.24 (0.05)	0.24 (0.05)
$\hat{H}^2_{ind\_SCA}$	0.39 (0.05)	0.50 (0.05)	0.38 (0.05)	0.31 (0.05)	0.31 (0.05)

See Table 2 for a description of traits.

[1] Variance components are described in Table S1. Within-site heritability estimates were calculated using the equations described in Table S3. All models converged.

† These terms were at boundary (very close to 1) after convergence of the model.

Table S6. For the complete **dataset 2**, variance components and within-site heritabilities estimated using the **final additive ABLUP-A and GBLUP-A models** (Eq. [7] in the manuscript[1]).

Variance components[2]	AV_{13}	WD_{13}	HT_{13}	DBH_{13}	VOL_{13}
**ABLUP-A**					
$\hat{\sigma}^2_{b\_ASS}$	0.02 (0.01)	62.50 (41.81)	1250.88 (704.81)	30.61 (18.73)	3.04 (1.87)
$\hat{\sigma}^2_{b\_SCA}$	0.01 (0.01)	24.33 (18.85)	687.06 (470.68)	15.37 (11.57)	2.78 (1.99)
$\hat{\sigma}^2_{p\_ASS}$	0.02 (0.01)	95.78 (33.22)	794.59 (219.00)	34.55 (10.16)	3.58 (1.02)
$\hat{\sigma}^2_{p\_SCA}$	0.01 (0.00)	66.73 (28.82)	597.20 (327.22)	18.34 (9.92)	3.53 (1.62)
$\hat{r}_{Ba}$	0.97 (0.05)	0.96 (0.07)	0.73 (0.12)	0.58 (0.17)	0.62 (0.16)
$\hat{\sigma}^2_{a\_ASS}$	0.07 (0.02)	357.80 (107.93)	2325.22 (739.18)	81.46 (28.43)	8.76 (2.96)
$\hat{\sigma}^2_{a\_SCA}$	0.05 (0.01)	232.12 (73.94)	4613.56 (1420.94)	91.34 (30.38)	16.14 (5.30)
$\hat{\sigma}^2_{e\_ASS}$	0.05 (0.01)	358.63 (66.02)	2504.11 (442.07)	133.58 (18.24)	13.04 (1.87)
$\hat{\sigma}^2_{e\_SCA}$	0.05 (0.01)	482.66 (52.96)	4778.36 (836.93)	171.47 (20.30)	26.18 (3.40)
$\hat{h}^2_{ind\_ASS}$	0.50 (0.12)	0.44 (0.11)	0.41 (0.11)	0.33 (0.10)	0.35 (0.10)
$\hat{h}^2_{ind\_SCA}$	0.43 (0.11)	0.30 (0.08)	0.46 (0.11)	0.32 (0.09)	0.35 (0.10)
**GBLUP-A**					
$\hat{\sigma}^2_{b\_ASS}$	0.02 (0.01)	66.47 (43.52)	1289.75 (726.66)	32.03 (19.52)	3.18 (1.95)
$\hat{\sigma}^2_{b\_SCA}$	0.01 (0.01)	25.96 (19.70)	695.15 (474.51)	15.51 (11.73)	2.87 (2.05)
$\hat{\sigma}^2_{p\_ASS}$	0.02 (0.01)	81.23 (31.56)	795.15 (225.94)	35.36 (10.47)	3.62 (1.05)
$\hat{\sigma}^2_{p\_SCA}$	0.02 (0.00)	60.99 (27.13)	603.23 (330.01)	20.22 (10.04)	3.64 (1.63)
$\hat{r}_{Ba}$	0.99 (0.05)	0.98 (0.06)	0.56 (0.16)	0.45 (0.20)	0.50 (0.18)
$\hat{\sigma}^2_{a\_ASS}$	0.05 (0.01)	320.22 (61.34)	1095.23 (305.39)	39.47 (12.87)	4.35 (1.33)
$\hat{\sigma}^2_{a\_SCA}$	0.04 (0.01)	247.26 (52.77)	2709.93 (659.49)	59.91 (17.05)	10.58 (2.90)
$\hat{\sigma}^2_{e\_ASS}$	0.06 (0.01)	397.29 (42.06)	3295.15 (271.69)	160.44 (12.61)	15.89 (1.27)
$\hat{\sigma}^2_{e\_SCA}$	0.05 (0.01)	469.35 (40.70)	5924.61 (502.60)	189.19 (15.06)	29.51 (2.41)
$\hat{h}^2_{ind\_ASS}$	0.40 (0.06)	0.40 (0.06)	0.21 (0.05)	0.17 (0.05)	0.18 (0.05)
$\hat{h}^2_{ind\_SCA}$	0.34 (0.06)	0.32 (0.06)	0.29 (0.06)	0.22 (0.06)	0.24 (0.06)

See Table 2 for a description of traits.

[1] The additive model fitted is described in Eq. [7], by excluding the dominance term $\boldsymbol{d}(\boldsymbol{s})$. All models converged.

[2] Variance components are described in Table S1. Within-site heritability estimates were calculated using the equations described in Table S3.

Table S7. For the complete **dataset 2**, variance components and within-site heritabilities estimated using the **final additive-dominance ABLUP-AD and GBLUP-AD models** (Eq. [7] in the manuscript[1]).

Variance components[1]	AV_{13}	WD_{13}	HT_{13}	DBH_{13}	VOL_{13}
**ABLUP-AD**					
$\hat{\sigma}^2_{b\_ASS}$	0.02 (0.01)	54.48 (36.92)	1158.85 (656.32)	26.67 (16.57)	2.69 (1.68)
$\hat{\sigma}^2_{b\_SCA}$	0.01 (0.01)	24.27 (18.81)	714.39 (484.80)	15.63 (11.63)	2.84 (2.01)
$\hat{\sigma}^2_{p\_ASS}$	0.02 (0.01)	75.83 (32.72)	723.01 (216.49)	29.33 (9.93)	3.10 (1.00)
$\hat{\sigma}^2_{p\_SCA}$	0.01 (0.00)	66.27 (28.67)	379.71 (315.85)	12.43 (9.62)	2.57 (1.57)
$\hat{r}_{Ba}$	0.97 (0.05)	0.99 (NA)†	0.59 (0.21)	0.25 (0.35)	0.36 (0.30)
$\hat{\sigma}^2_{a\_ASS}$	0.07 (0.02)	312.78 (108.59)	1719.38 (705.40)	47.12 (27.45)	5.84 (2.91)
$\hat{\sigma}^2_{a\_SCA}$	0.05 (0.01)	231.73 (73.98)	2366.36 (1295.41)	53.90 (28.98)	9.23 (5.08)
$\hat{r}_{Bd}$	NA†*	NA†*	0.99 (NA)†	0.99 (NA)†	0.99 (NA)†
$\hat{\sigma}^2_{d\_ASS}$	0.00 (NA)†	160.69 (103.47)	900.78 (705.30)	66.57 (41.35)	5.88 (3.86)
$\hat{\sigma}^2_{d\_SCA}$	0.00 (NA)†	0.00 (NA)†	3314.13 (1750.79)	72.44 (42.03)	12.61 (7.21)
$\hat{\sigma}^2_{e\_ASS}$	0.05 (0.01)	261.12 (93.07)	2132.55 (567.36)	100.83 (29.23)	10.08 (2.84)
$\hat{\sigma}^2_{e\_SCA}$	0.05 (0.01)	482.71 (52.96)	3403.84 (1200.11)	135.43 (30.78)	20.10 (5.15)
$\hat{h}^2_{ind\_ASS}$	0.50 (0.12)	0.39 (0.11)	0.31 (0.11)	0.19 (0.11)	0.23 (0.11)
$\hat{h}^2_{ind\_SCA}$	0.43 (0.11)	0.30 (0.08)	0.25 (0.13)	0.20 (0.10)	0.21 (0.11)
$\hat{d}^2_{ind\_ASS}$	0.00 (0.00)	0.20 (0.13)	0.16 (0.13)	0.27 (0.17)	0.24 (0.15)
$\hat{d}^2_{ind\_SCA}$	0.00 (0.00)	0.00 (0.00)	0.35 (0.18)	0.26 (0.15)	0.28 (0.16)
$\hat{H}^2_{ind\_ASS}$	0.50 (0.12)	0.58 (0.14)	0.48 (0.12)	0.47 (0.13)	0.47 (0.13)
$\hat{H}^2_{ind\_SCA}$	0.43 (0.11)	0.30 (0.08)	0.60 (0.14)	0.46 (0.12)	0.49 (0.13)

Table S7 [Continued]. For the complete **dataset 2**, variance components and within-site heritabilities estimated using the **final additive-dominance ABLUP-AD and GBLUP-AD models** (Eq. [7] in the manuscript[1]).

Variance components[1]	AV_{13}	WD_{13}	HT_{13}	DBH_{13}	VOL_{13}
**GBLUP-AD**					
$\hat{\sigma}^2_{b\_ASS}$	0.02 (0.01)	60.57 (40.10)	1210.06 (683.02)	28.57 (17.58)	2.92 (1.80)
$\hat{\sigma}^2_{b\_SCA}$	0.01 (0.01)	25.70 (19.46)	680.72 (461.58)	14.73 (11.05)	2.78 (1.98)
$\hat{\sigma}^2_{p\_ASS}$	0.02 (0.01)	73.91 (31.07)	689.70 (220.60)	30.38 (10.12)	3.12 (1.01)
$\hat{\sigma}^2_{p\_SCA}$	0.02 (0.00)	55.91 (26.87)	467.63 (320.06)	16.09 (9.73)	3.11 (1.59)
$\hat{r}_{Ba}$	0.99 (0.06)	0.99 (NA)†	0.42 (0.23)	0.20 (0.28)	0.30 (0.25)
$\hat{\sigma}^2_{a\_ASS}$	0.05 (0.01)	296.90 (62.18)	822.24 (292.56)	28.10 (12.28)	3.27 (1.28)
$\hat{\sigma}^2_{a\_SCA}$	0.03 (0.01)	239.73 (53.22)	1803.28 (623.62)	41.53 (16.30)	7.23 (2.78)
$\hat{r}_{Bd}$	0.99 (NA)†	0.67 (0.50)	0.81 (0.41)	0.99 (NA)†	0.99 (NA)†
$\hat{\sigma}^2_{d\_ASS}$	0.01 (0.01)	94.85 (54.19)	614.01 (338.75)	28.88 (13.90)	2.57 (1.31)
$\hat{\sigma}^2_{d\_SCA}$	0.01 (0.01)	46.95 (46.59)	1440.56 (599.60)	35.77 (15.90)	6.10 (2.68)
$\hat{\sigma}^2_{e\_ASS}$	0.06 (0.01)	336.15 (52.38)	2958.92 (328.84)	144.24 (14.42)	14.52 (1.42)
$\hat{\sigma}^2_{e\_SCA}$	0.05 (0.01)	435.12 (50.51)	5220.25 (589.61)	170.13 (17.15)	26.25 (2.77)
$\hat{h}^2_{ind\_ASS}$	0.37 (0.06)	0.37 (0.06)	0.16 (0.05)	0.12 (0.05)	0.14 (0.05)
$\hat{h}^2_{ind\_SCA}$	0.32 (0.06)	0.31 (0.06)	0.20 (0.06)	0.16 (0.06)	0.17 (0.06)
$\hat{d}^2_{ind\_ASS}$	0.06 (0.06)	0.12 (0.07)	0.12 (0.07)	0.12 (0.06)	0.11 (0.06)
$\hat{d}^2_{ind\_SCA}$	0.05 (0.05)	0.06 (0.06)	0.16 (0.07)	0.14 (0.06)	0.14 (0.06)
$\hat{H}^2_{ind\_ASS}$	0.43 (0.07)	0.49 (0.07)	0.28 (0.07)	0.25 (0.06)	0.25 (0.06)
$\hat{H}^2_{ind\_SCA}$	0.37 (0.07)	0.37 (0.07)	0.36 (0.07)	0.29 (0.07)	0.31 (0.07)

See Table 2 for a description of traits.

[1] Variance components are described in Table S1. Within-site heritability estimates were calculated using the equations described in Table S3. All models converged.

† These terms were at boundary (very close to 0 or 1) after convergence of the model.

* These type-B correlation terms were not estimable because the dominance variance is null on one or both sites (division by 0).

Table S8. For the complete **dataset 2**, across-site genetic parameters estimated using the ABLUP and GBLUP additive (A) and additive-dominance (AD) models.

	AV_{13}		WD_{13}		HT_{13}		DBH_{13}		VOL_{13}	
	A[1]	AD[2]	A[1]	AD[2]	A[1]	AD[2]	A[1]	AD[2]	A[1]	AD[2]
**ABLUP**										
$\hat{h}^2_{ind}$	0.46 (0.10)	0.46 (0.10)	0.35 (0.09)	0.34 (0.09)	0.32 (0.10)	0.16 (0.10)	0.19 (0.08)	0.05 (0.08)	0.22 (0.08)	0.08 (0.09)
$\hat{d}^2_{ind}$	—	0.00 (0.00)	—	0.10 (0.06)	—	0.28 (0.13)	—	0.27 (0.12)	—	0.27 (0.12)
$\hat{H}^2_{ind}$	—	0.46 (0.10)	—	0.44 (0.09)	—	0.44 (0.12)	—	0.32 (0.10)	—	0.34 (0.11)
$\hat{r}_{Ba}$	0.97 (0.05)	0.97 (0.05)	0.96 (0.07)	0.99 (NA)†	0.73 (0.12)	0.59 (0.21)	0.58 (0.17)	0.25 (0.35)	0.62 (0.16)	0.36 (0.30)
$\hat{r}_{Bg}$	—	NA*	—	0.81 (0.11)	—	0.76 (0.11)	—	0.68 (0.13)	—	0.70 (0.12)
AIC	**-1923**	-1917	**11130**	11133	15578	**15571**	10342	**10334**	7056	**7048**
BIC	**-1876**	-1854	**11178**	11196	**15626**	15635	**10390**	10398	**7104**	7113
$\hat{V}_A$ (%)[3]	—	100	—	77	—	37	—	15	—	23
$\hat{V}_D$ (%)[3]	—	0	—	23	—	63	—	85	—	77
**GBLUP**										
$\hat{h}^2_{ind}$	0.37 (0.05)	0.34 (0.05)	0.35 (0.05)	0.34 (0.05)	0.15 (0.05)	0.08 (0.05)	0.09 (0.04)	0.03 (0.04)	0.11 (0.05)	0.05 (0.04)
$\hat{d}^2_{ind}$	—	0.06 (0.04)	—	0.06 (0.05)	—	0.12 (0.05)	—	0.13 (0.04)	—	0.13 (0.04)
$\hat{H}^2_{ind}$	—	0.40 (0.05)	—	0.40 (0.06)	—	0.20 (0.06)	—	0.16 (0.05)	—	0.18 (0.05)
$\hat{r}_{Ba}$	0.99 (0.05)	0.99 (0.06)	0.98 (0.06)	0.99 (NA)†	0.56 (0.16)	0.42 (0.23)	0.45 (0.20)	0.20 (0.28)	0.50 (0.18)	0.30 (0.25)
$\hat{r}_{Bg}$	—	0.99 (0.05)	—	0.93 (0.11)	—	0.59 (0.17)	—	0.59 (0.15)	—	0.61 (0.14)
AIC	**-1938**	-1935	11091	11091	15594	**15577**	10351	**10334**	7066	**7051**
BIC	**-1890**	-1871	**11138**	11155	15642	15641	10399	10398	7115	7115
$\hat{V}_A$ (%)[3]	—	85	—	85	—	40	—	18	—	27
$\hat{V}_D$ (%)[3]	—	15	—	15	—	60	—	82	—	73

The parameters reported are individual narrow-sense ($\hat{h}^2_{ind}$) and broad-sense heritability ($\hat{H}^2_{ind}$), the dominance ratio ($\hat{d}^2_{ind}$), and the narrow-sense ($\hat{r}_{Ba}$) and broad-sense ($\hat{r}_{Bg}$) type-B genetic correlations. Standard errors of estimates are in parentheses. The AIC and BIC of the best models between the A and AD models are in bold (Δ>2). The details of all variance components and within-site genetic parameter estimates are in Tables S6 (A models) and Table S7 (AD models). See Table 2 for a description of traits.

[1] The additive model fitted is described in Eq. [7], by excluding the dominance term $\boldsymbol{d}(\boldsymbol{s})$.

[2] The additive-dominance model fitted is described in Eq. [7].

[3] The across-site additive ($\hat{V}_A = \hat{r}_{Ba}\overline{\hat{\sigma}^2_a}$) and dominance variances ($\hat{V}_D = \hat{r}_{Bd}\overline{\hat{\sigma}^2_d}$) are reported as a percentage of the total genetic variance ($\hat{V}_A + \hat{V}_D$).

† These terms were at boundary (very close to 1) after convergence of the model.

* This broad-sense type-B correlation was not estimable because the estimated dominance variance on both sites is zero (Table S7; division by 0).

Table S9. For the complete **dataset 2**, average across-site predictive ability (*PA*) and prediction accuracy (*PACC*) obtained from cross-validation using the ABLUP and GBLUP additive (A) and additive-dominance (AD) models.

	AV_{13}		WD_{13}		HT_{13}		DBH_{13}		VOL_{13}	
	A[1]	AD[2]	A[1]	AD[2]	A[1]	AD[2]	A[1]	AD[2]	A[1]	AD[2]
**ABLUP**										
**CV1**[3]										
PA_{BV}[4]	0.41 (0.02)	0.41 (0.02)	0.42 (0.05)	0.42 (0.05)	0.37 (0.01)	0.36 (0.01)	0.32 (0.01)	0.30 (0.01)	0.34 (0.01)	0.32 (0.01)
PA_{GV}[4]	—	0.41 (0.02)	—	0.43 (0.06)	—	0.39 (0.01)	—	0.34 (0.01)	—	0.36 (0.01)
$PACC_{BV}$[5]	0.67 (0.01)	0.70 (0.01)	0.69 (0.04)	0.71 (0.04)	0.75 (0.06)	0.85 (0.07)	0.73 (0.05)	0.81 (0.06)	0.74 (0.06)	0.83 (0.06)
$PACC_{GV}$[5]	—	0.65 (0.01)	—	0.65 (0.04)	—	0.68 (0.05)	—	0.66 (0.04)	—	0.68 (0.06)
**CV2**[3]										
PA_{BV}[4]	0.34 (0.02)	0.34 (0.03)	0.33 (0.02)	0.33 (0.02)	0.20 (0.02)	0.16 (0.05)	0.13 (0.03)	0.11 (0.03)	0.16 (0.02)	0.12 (0.03)
PA_{GV}[4]	—	0.34 (0.03)	—	0.33 (0.02)	—	0.16 (0.05)	—	0.11 (0.03)	—	0.12 (0.03)
$PACC_{BV}$[5]	0.56 (0.02)	0.58 (0.03)	0.55 (0.02)	0.57 (0.02)	0.41 (0.05)	0.37 (0.14)	0.30 (0.06)	0.29 (0.08)	0.34 (0.05)	0.31 (0.08)
$PACC_{GV}$[5]	—	0.53 (0.03)	—	0.51 (0.02)	—	0.28 (0.10)	—	0.21 (0.06)	—	0.23 (0.06)
**GBLUP**										
**CV1**[3]										
PA_{BV}[4]	0.43 (0.01)	0.43 (0.01)	0.45 (0.04)	0.45 (0.04)	0.36 (0.01)	0.34 (0.01)	0.30 (0.01)	0.29 (0.01)	0.32 (0.01)	0.31 (0.01)
PA_{GV}[4]	—	0.43 (0.01)	—	0.46 (0.04)	—	0.37 (0.01)	—	0.33 (0.01)	—	0.35 (0.01)
$PACC_{BV}$[5]	0.70 (0.02)	0.73 (0.02)	0.76 (0.02)	0.78 (0.03)	0.71 (0.04)	0.80 (0.04)	0.69 (0.03)	0.77 (0.03)	0.70 (0.04)	0.78 (0.04)
$PACC_{GV}$[5]	—	0.68 (0.02)	—	0.70 (0.02)	—	0.66 (0.03)	—	0.64 (0.02)	—	0.66 (0.04)
**CV2**[3]										
PA_{BV}[4]	0.33 (0.02)	0.32 (0.02)	0.37 (0.02)	0.38 (0.02)	0.18 (0.02)	0.18 (0.03)	0.13 (0.04)	0.14 (0.03)	0.15 (0.03)	0.15 (0.02)
PA_{GV}[4]	—	0.32 (0.02)	—	0.37 (0.02)	—	0.22 (0.03)	—	0.20 (0.03)	—	0.20 (0.02)
$PACC_{BV}$[5]	0.55 (0.03)	0.55 (0.04)	0.62 (0.02)	0.65 (0.02)	0.37 (0.05)	0.42 (0.09)	0.30 (0.07)	0.38 (0.07)	0.33 (0.05)	0.39 (0.06)
$PACC_{GV}$[5]	—	0.50 (0.03)	—	0.57 (0.03)	—	0.39 (0.07)	—	0.38 (0.04)	—	0.38 (0.05)

Standard deviations of estimates across repetitions are in parentheses. For the ABLUP-AD models and CV2, the predicted dominance deviations were null for all individuals, such that the predicted genetic values were equal to the predicted breeding values. Hence, the PA_{BV} was equal to PA_{GV}. See Table 2 for a description of traits.

[1] The additive model fitted is described in Eq. [7], by excluding the dominance term $\boldsymbol{d}(\boldsymbol{s})$.

[2] The additive-dominance model fitted is described in Eq. [7].

[3] CV1: trees were randomly split into 10 folds, making sure that each fold contained ~10% of the trees from each family; CV2: families were randomly split into 10 folds.

[4] The predictive ability of breeding values (PA_{BV}) and of genetic values (PA_{GV}) were calculated as the correlation between the predicted breeding or genetic values and the observed phenotypes, respectively, within each site. The average across sites is reported.

[5] The prediction accuracy of breeding values ($PACC_{BV}$) was equal to $PA_{BV}/\sqrt{\hat{h}^2_{ind}}$. The prediction accuracy of genetic values ($PACC_{GV}$) was equal to $PA_{GV}/\sqrt{\hat{H}^2_{ind}}$. The heritabilities obtained using GBLUP were used to calculate the *PACC* of ABLUP and GBLUP models. The average across sites is reported.

Table S10. For the complete **dataset 1**, average predictive ability (PA_{BV}) and prediction accuracy ($PACC_{BV}$) of breeding values obtained on site Asselin (ASS) and St-Casimir (SCA) using the GBLUP-A method and CV1. See Table 2 for a description of traits.

Trait	PA_{BV} ASS	PA_{BV} SCA	% difference	$PACC_{BV}$ ASS	$PACC_{BV}$ SCA	% difference
AV_{16}	0.46 (0.01)	0.50 (0.00)	8.7	0.85 (0.01)	0.84 (0.00)	1.9
WD_{16}	0.52 (0.00)	0.57 (0.00)	9.6	0.93 (0.01)	0.87 (0.01)	-6.5
HT_{16}	0.35 (0.00)	0.41 (0.00)	17.1	0.73 (0.01)	0.79 (0.00)	8.2
DBH_{16}	0.31 (0.00)	0.40 (0.00)	29.0	0.85 (0.01)	0.88 (0.01)	3.5
VOL_{16}	0.34 (0.00)	0.40 (0.00)	17.7	0.83 (0.01)	0.84 (0.01)	1.2

Table S11. For **dataset 1**, percentage of families for which both parents appeared in only one cross when varying the number of families in resampling simulations. These families were unrelated to the training dataset when they were assigned to the validation dataset in the CV2 cross-validation scenario.

Number of families sampled	Percentage of families with both parents appearing in a single cross	SD
12	62%	17%
20	30%	12%
28	23%	5%
36	8%	6%
48	3%	1%
60	0%	1%
72	0%	0%
