# Supplementary material for: Increasing genomic prediction accuracy for unphenotyped full-sib families by modeling additive and dominance effects with large datasets in white spruce
Source: Front Plant Sci. 2023 Mar 22;14:1137834. doi: 10.3389/fpls.2023.1137834 (PMC10073444; doi:10.3389/fpls.2023.1137834)
Supplement: Supplementary file 2 [file DataSheet_2.pdf]

SUPPLEMENTARY FIGURES:

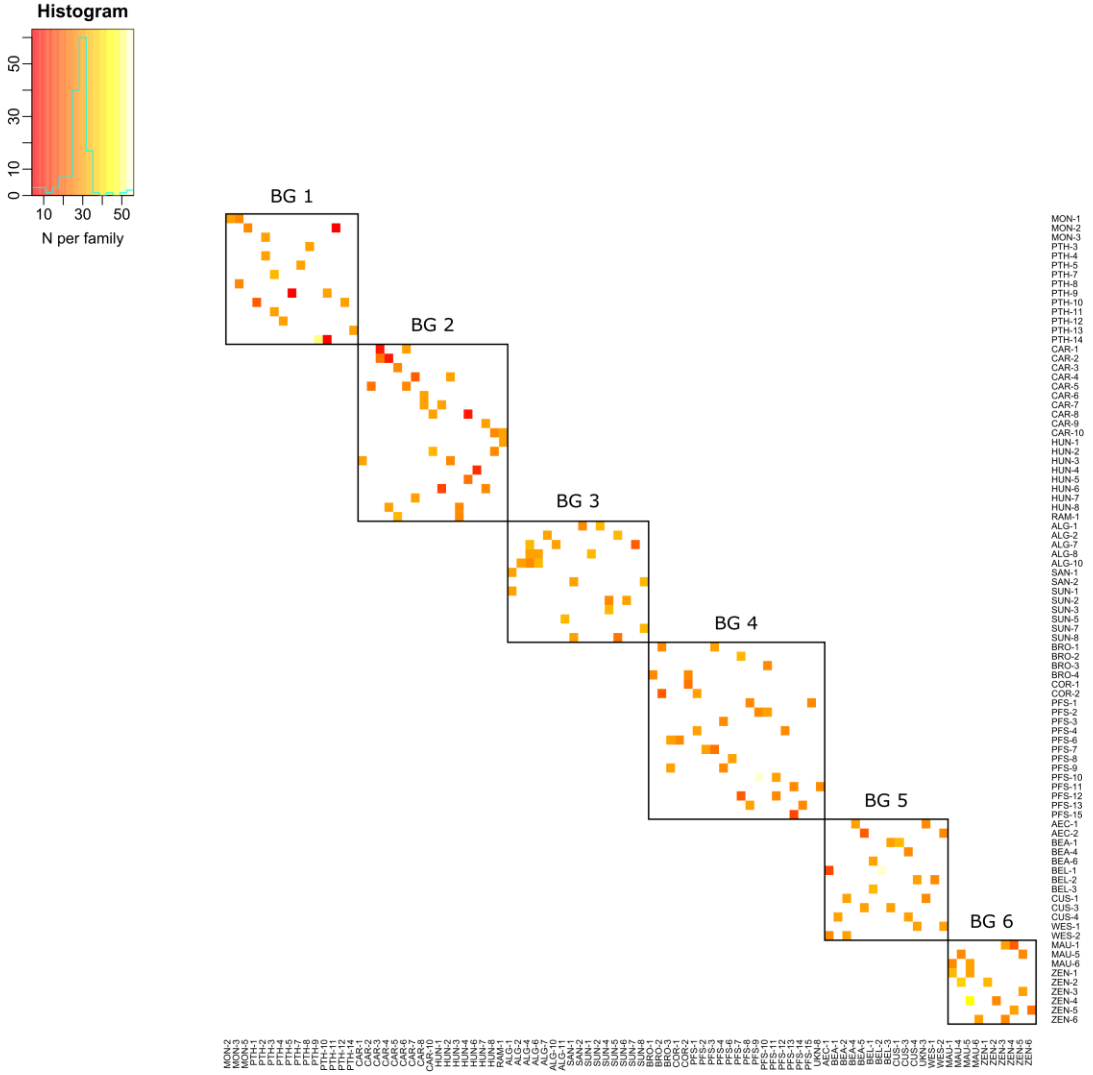

Figure S1. Partial diallel mating design for the six breeding groups (BGs, black rectangles) and the 4,066 trees used for analyses. Mothers and fathers are on the x and y axes, respectively. Each colored square represent a full-sib family and the colors indicate the number of trees sampled per full-sib family (top left color key with histogram). Dataset 1 included BGs 1, 2, 5 and 6, and dataset 2 included BGs 3 and 4.

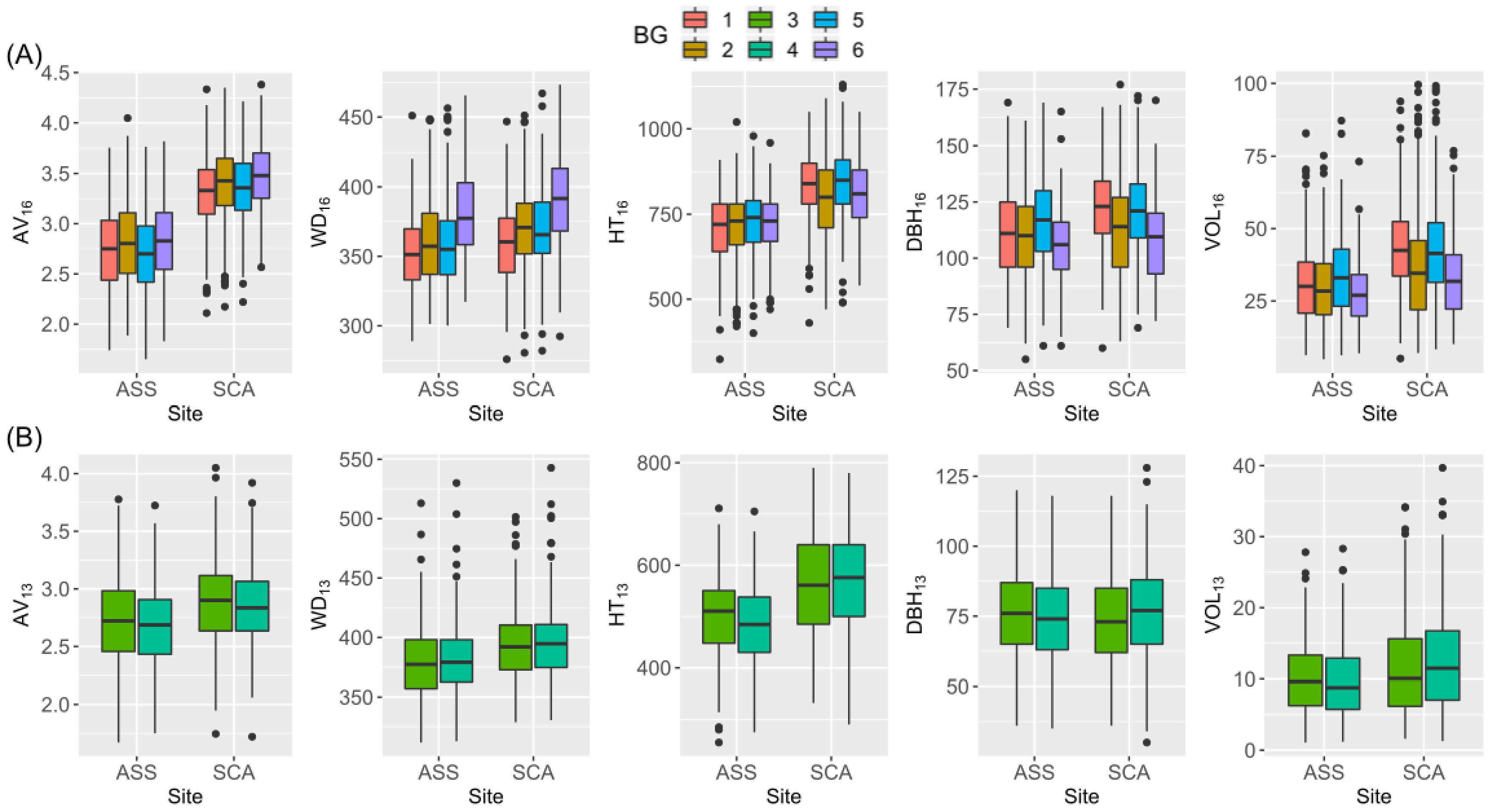

Figure S2. Boxplots of phenotypes for **(A)** dataset 1, that is breeding groups (BGs) 1, 2, 5, and 6; and for **(B)** dataset 2, that is BGs 3 and 4. Trees for BGs 1, 2, 5 and 6 were 16 years old, whereas those of BGs 3 and 4 were 13 yeas old. The phenotypes are grouped by sites Asselin (ASS) and St. Casimir (SCA) on the x-axis, and are colored by BG. See Table 2 for a description of traits.

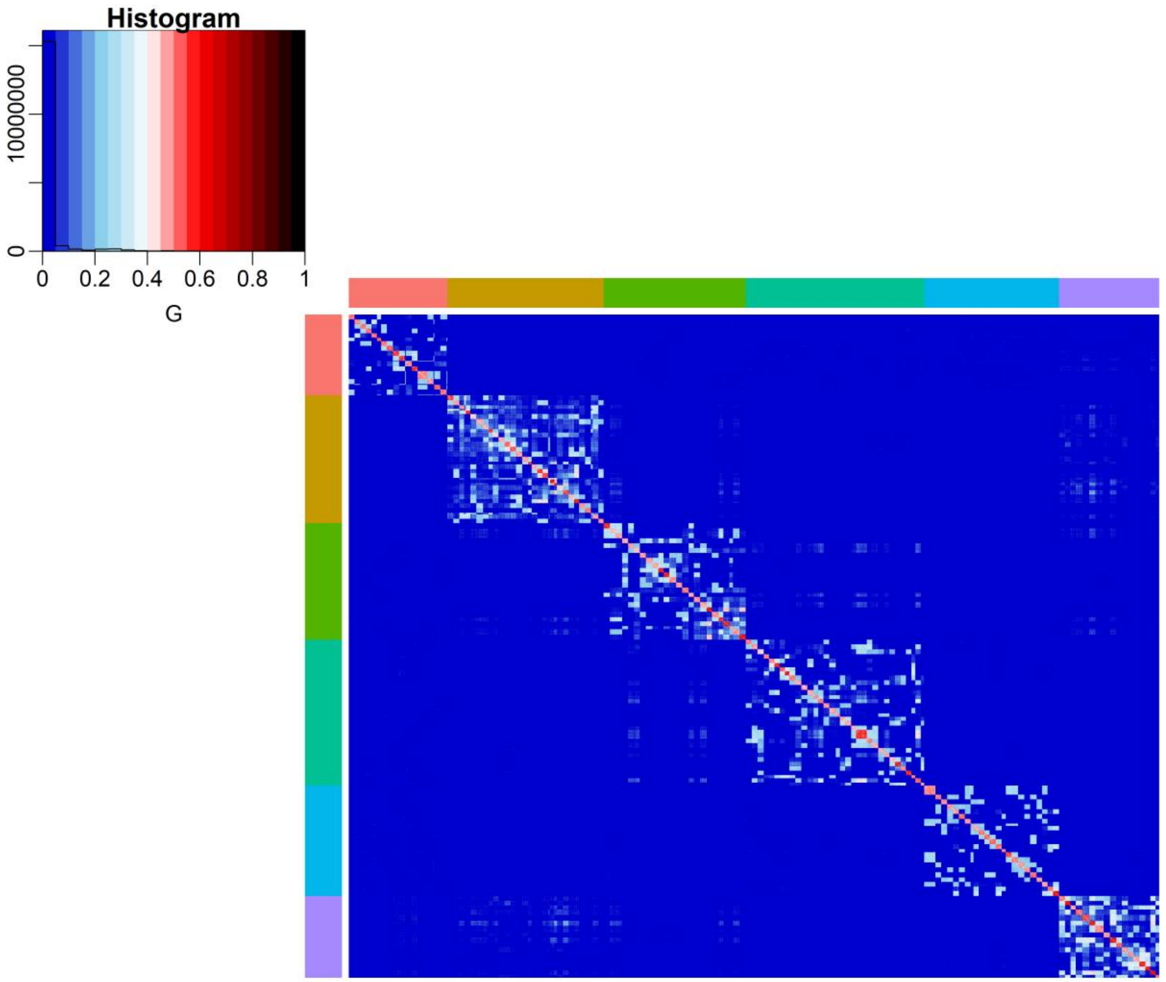

Figure S3. Realized genomic additive relationship matrix ($\boldsymbol{G_a}$). The histogram on the top left represents the color key for the values of $\boldsymbol{G_a}$. The side colors on the x and y-axes represent the six breeding groups (BGs) in order 1, 2, 3, 4, 5, and 6 from top to bottom. Dataset 1 included the BGs 1, 2, 5 and 6, and dataset 2 included the BGs 3 and 4. The values of $\boldsymbol{G_a}$ vary around 0.5 between full-sibs, around 0.25 between half-sibs, and around 0 for unrelated trees. The mean additive genomic relationship within full-sib families was 0.511 with a standard deviation of 0.072.

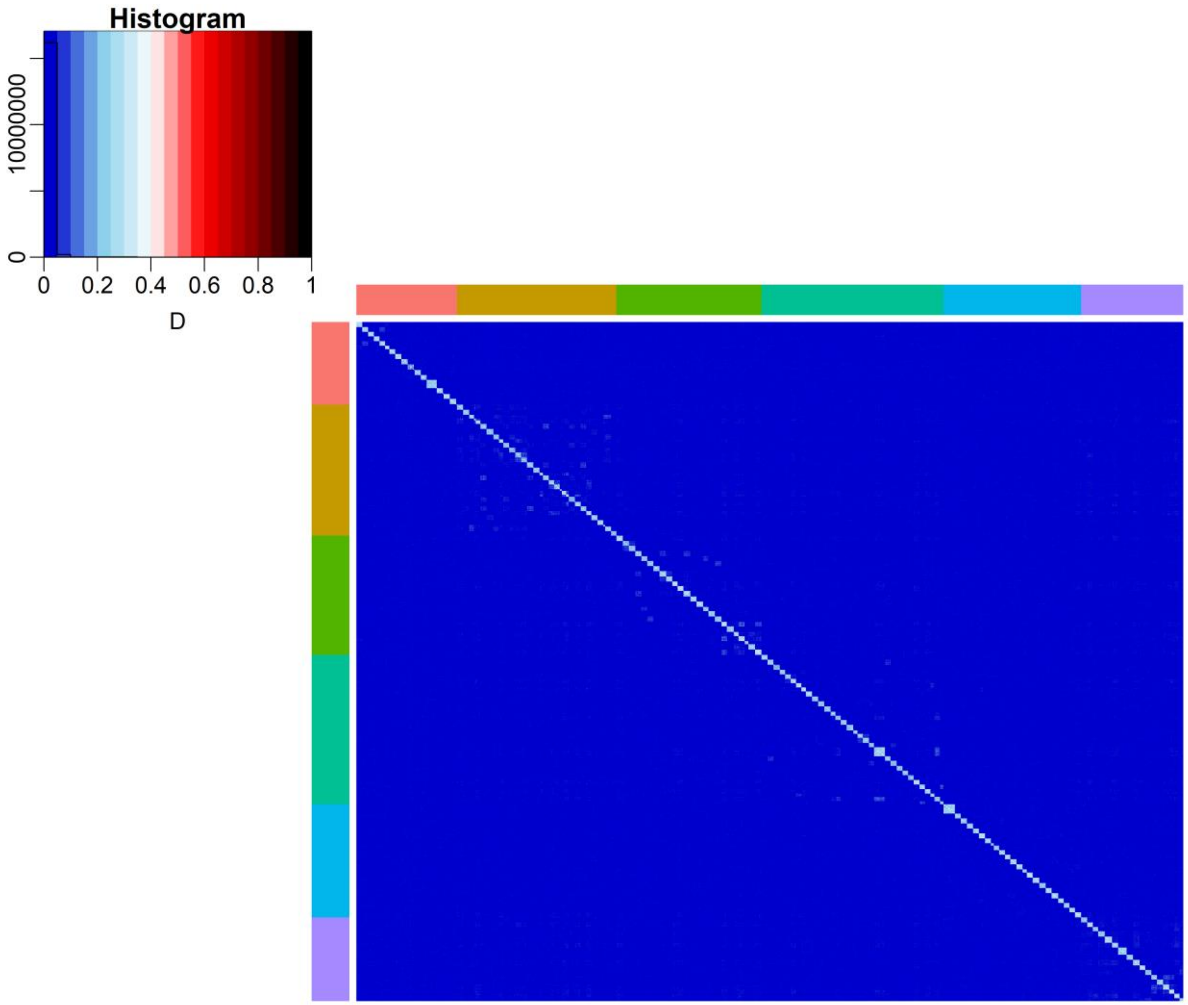

Figure S4. Realized genomic dominance relationship matrix ($\boldsymbol{G_d}$). The histogram on the top left represents the color key for the values of $\boldsymbol{G_d}$. The side colors on the x and y-axes represent the six breeding groups (BGs) in order 1, 2, 3, 4, 5, and 6 from top to bottom. Dataset 1 included the BGs 1, 2, 5 and 6, and dataset 2 included the BGs 3 and 4. The values of $\boldsymbol{G_d}$ vary around 0.25 between full-sibs, and around zero otherwise. The mean dominance genomic relationship within full-sib families was 0.251 with a standard deviation of 0.067.

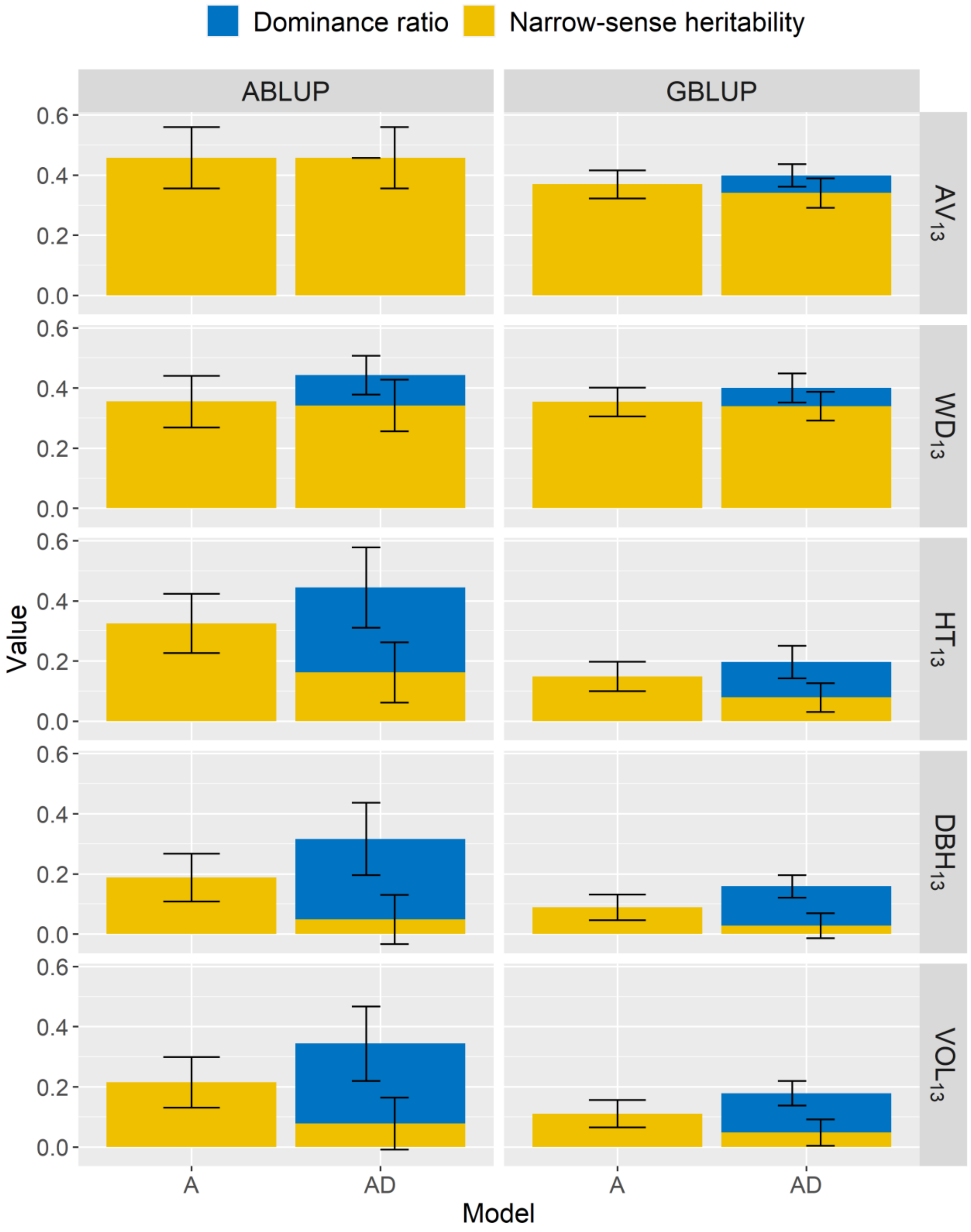

Figure S5. For the complete **dataset 2**, across-site narrow-sense heritabilities ($\hat{h}^2_{ind}$) and dominance ratios ($\hat{d}^2_{ind}$) estimated using additive (A) or additive-dominance (AD) models, with ABLUP or GBLUP. The broad-sense heritabilities ($\hat{H}^2_{ind}$) are estimated as the sum of $\hat{h}^2_{ind}$ and $\hat{d}^2_{ind}$. The error bars represent the approximated standard errors of estimates calculated using the delta method. See Table 2 for a description of traits.

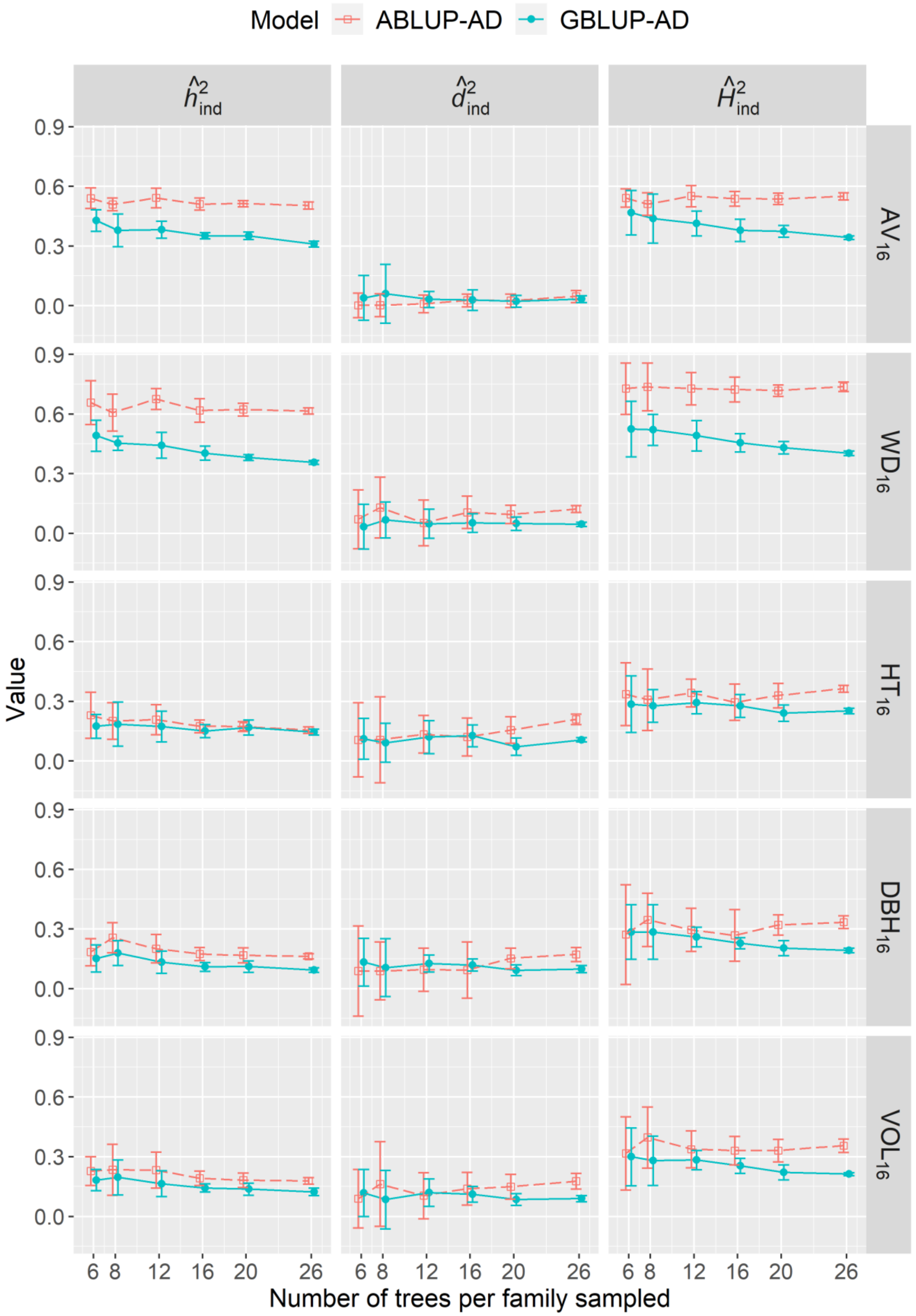

Figure S6. For **dataset 1**, across-site narrow-sense heritabilities ($\hat{h}^2_{ind}$), dominance ratios ($\hat{d}^2_{ind}$), and broad-sense heritabilities ($\widehat{H}^2_{ind}$) estimated when varying the number of trees per family sampled using the ABLUP-AD and GBLUP-AD additive-dominance models. The error bars represent the standard deviation of estimates across the 10 replications for each level of number of trees per family sampled. See Table 2 for a description of traits.

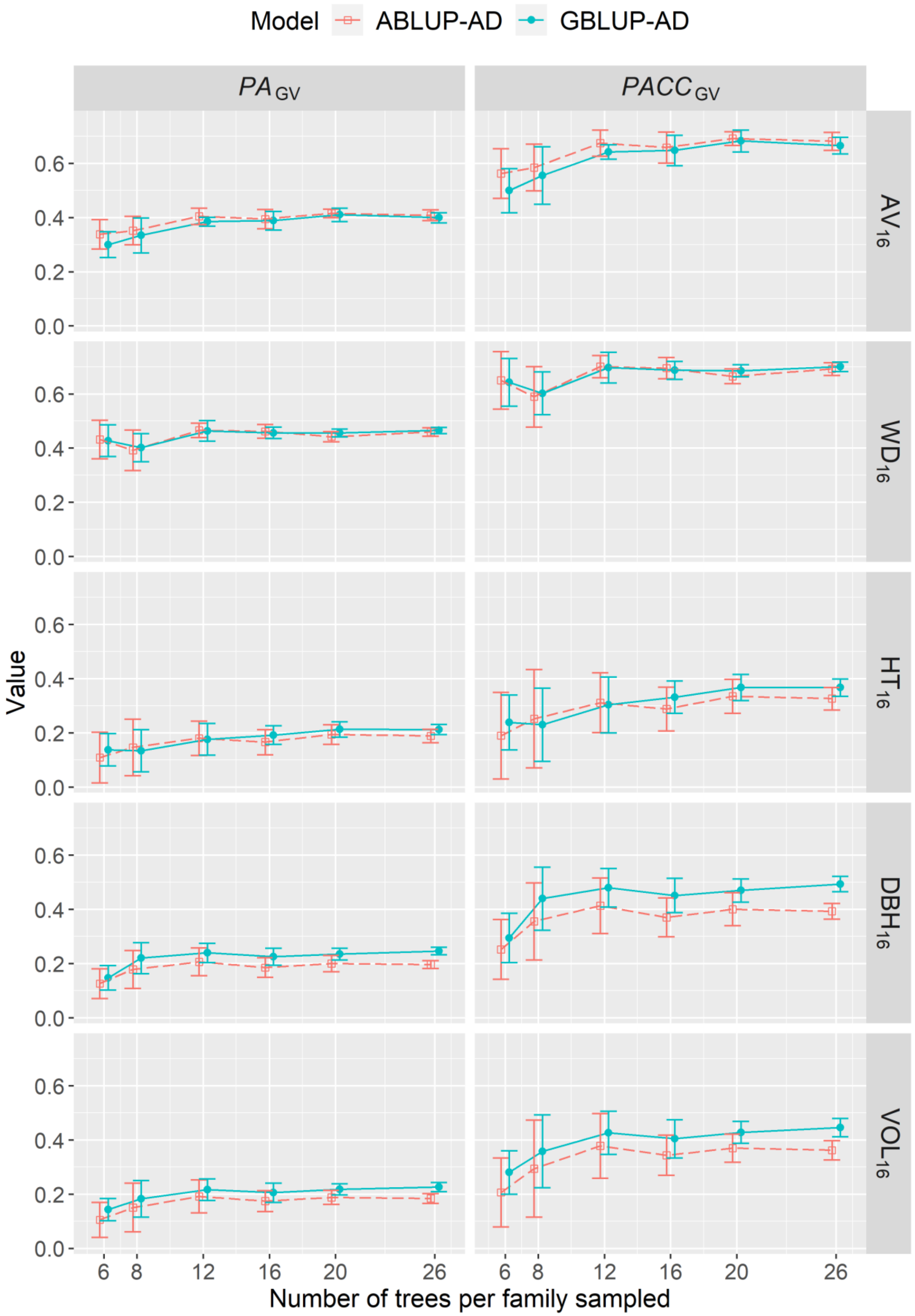

Figure S7. For **dataset 1**, Predictive ability (*PA_{GV}*) and prediction accuracy of genetic values (*$PACC_{GV}$*) estimated using CV2 and when varying the number of trees per family sampled using the ABLUP-AD and GBLUP-AD additive-dominance models. The error bars represent the standard deviation of estimates across the 10 replications for each level of number of trees per family sampled. See Table 2 for a description of traits.

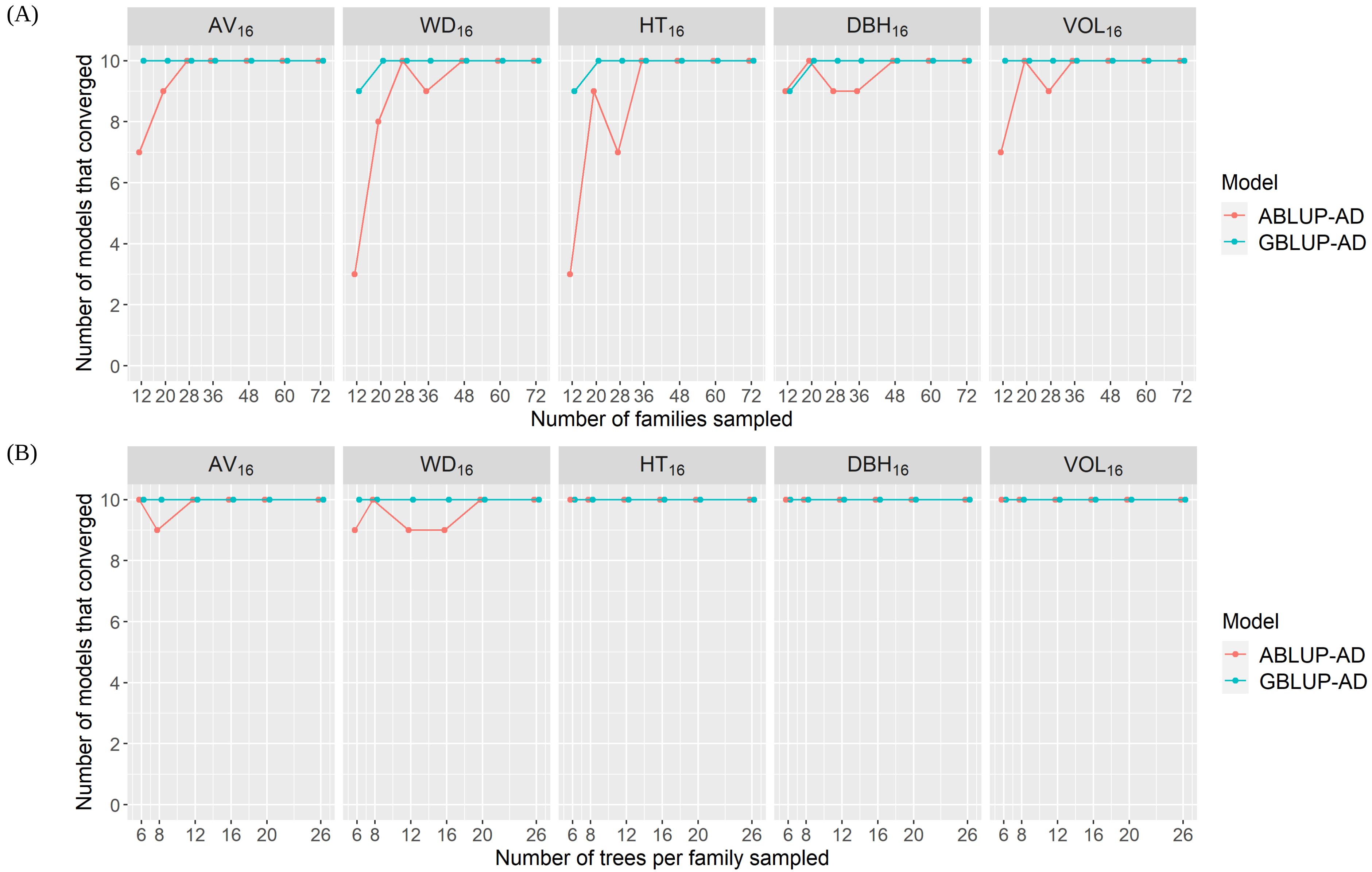

Figure S8. For **dataset 1**, number of models that converged using the ABLUP-AD and GBLUP-AD additive-dominance models when **(A)** varying the number of families sampled; and **(B)** varying the number of trees per family sampled. See Table 2 for a description of traits.
