# Supplementary material for: Increasing genomic prediction accuracy for unphenotyped full-sib families by modeling additive and dominance effects with large datasets in white spruce
Source: Front Plant Sci. 2023 Mar 22;14:1137834. doi: 10.3389/fpls.2023.1137834 (PMC10073444; doi:10.3389/fpls.2023.1137834)
Supplement: Supplementary file 3 [file DataSheet_3.pdf]

Supplementary Methods:

Production of the genotyping dataset

A total of 4,245 trees were genotyped using an Infinium iSelect SNP array (Illumina, San Diego, CA) containing 5,308 SNPs as described in Lenz et al. (2020a) and as a subset of Pavy et al. (2013). The 5,308 SNPs represented as many white spruce genes distributed over the 12 linkage groups. The average genotyping reproducibility rate was high (99.99%), as estimated from positive controls replicated on each genotyping plates. The following SNP quality criteria were applied: a genotyping reproducibility ≥ 98%, a call rate ≥ 85%, a fixation index $|F_e| \leq 0.50$, and a minor allele frequency (MAF) ≥ 0.01. The Mendelian segregation of each SNP was also verified using a subset of 2,700 white spruce trees from 80 full-sib crosses for which we had parent genotypes on the same set of SNPs. SNPs with ≥ 5% of genotyping errors, that is incompatible genotypes according to parent genotypes, or for which genotype frequencies departed from Mendelian expectations in more than 25% of families (Fisher exact test, P-value < 0.05) were discarded. We retained a total of 4,092 high-quality SNPs. Missing genotypes (only 0.9% of genotypes) were imputed using a k-nearest neighbor method based on linkage disequilibrium (LD-kNNi) with the software LinkImpute (Money et al., 2015). The software estimated an accuracy of 0.87 for imputed genotypes by randomly masking 10,000 genotypes.

Detection of pedigree errors

Prior to quantitative genetic analyses, we assigned parentage for the 4,243 retained trees using available SNP data with the software CERVUS v.3.0 (Marshall et al., 1998; Kalinowski et al., 2007) and compared the results against the registered pedigree. We ran CERVUS assuming that both female and male parents of the 4,243 trees were unknown (i.e., “parent pair-sexes

unknown analysis”) and including the 101 candidate parents genotyped on a subset of 657 SNPs. We determined significance of parental assignments by using the delta score. The critical delta score to assign parent pairs with 95% confidence was determined by simulating 10,000 offspring, assuming a genotyping error rate of 5% and that 90% of candidate parents were sampled. Out of the 431 trees (10.2%) for which parental assignments did not match the registered pedigree, 265 could be reclassified into an existing full-sib family based on both assigned parents. The rest of the trees showing pedigree errors (166) were discarded for further analyses. In addition, after checking model residuals of preliminary quantitative genetic analyses using the ABLUP and GBLUP models described in this study, we discarded four outlier trees: two outliers for both HT and DBH, and two outliers for AV. We also discarded six trees out of six pairs of trees with identical genotypes. Finally, one tree was discarded because all other trees in this family were discarded due to pedigree errors, thus resulting in a too small family for reliable statistical inferences. The final dataset comprised 4,066 trees from 146 full-sib families. Those families involved 101 parents, with an average of 27.9 trees per family and 14.0 trees per family on each site. The reclassification of individuals with pedigree errors into existing families led to a few large families of more than 40 trees (Table 1).

References

Kalinowski, S. T., Taper, M. L., and Marshall, T. C. (2007). Revising how the computer program cervus accommodates genotyping error increases success in paternity assignment. *Molecular Ecology* 16, 1099–1106. doi: 10.1111/j.1365-294X.2007.03089.x.

Lenz, P. R. N., Nadeau, S., Azaiez, A., Gérardi, S., Deslauriers, M., Perron, M., et al. (2020a). Genomic prediction for hastening and improving efficiency of forward selection in conifer polycross mating designs: an example from white spruce. *Heredity* 124, 562–578. doi: 10.1038/s41437-019-0290-3.

Marshall, T. C., Slate, J., Kruuk, L. E. B., and Pemberton, J. M. (1998). Statistical confidence for likelihood-based paternity inference in natural populations. *Molecular Ecology* 7, 639–655. doi: 10.1046/j.1365-294x.1998.00374.x.

Money, D., Gardner, K., Migicovsky, Z., Schwaninger, H., Zhong, G.-Y., and Myles, S. (2015). LinkImpute: Fast and Accurate Genotype Imputation for Nonmodel Organisms. *G3 Genes|Genomes|Genetics* 5, 2383–2390. doi: 10.1534/g3.115.021667.

Pavy, N., Gagnon, F., Rigault, P., Blais, S., Deschênes, A., Boyle, B., et al. (2013). Development of high-density SNP genotyping arrays for white spruce (*Picea glauca*) and transferability to subtropical and nordic congeners. *Molecular Ecology Resources* 13, 324–336. doi: 10.1111/1755-0998.12062.
