# Supplementary material for: Increasing genomic prediction accuracy for unphenotyped full-sib families by modeling additive and dominance effects with large datasets in white spruce
Source: Front Plant Sci. 2023 Mar 22;14:1137834. doi: 10.3389/fpls.2023.1137834 (PMC10073444; doi:10.3389/fpls.2023.1137834)
Supplement: Supplementary file 4 [file DataSheet_4.pdf]

Example R code to run the additive-dominance ABLUP-AD and GBLUP-AD models presented in Nadeau et al. (2023, Frontiers in Plant Science).

Simon Nadeau

February 23 2023

Loading the phenotype file

This file includes 2458 phenotyped trees from dataset 1.

```
pheno_DB.training.cleaned = read.csv("pheno_clean.csv", header = T, stringsAsFactors = F)

#Create a second, identical, ID column for modeling dominance.
pheno_DB.training.cleaned$Ind2 = pheno_DB.training.cleaned$Ind

#Converting to factors.
pheno_DB.training.cleaned$Ind = as.factor(pheno_DB.training.cleaned$Ind)
pheno_DB.training.cleaned$Ind2 = as.factor(pheno_DB.training.cleaned$Ind2)
pheno_DB.training.cleaned$Site = as.factor(pheno_DB.training.cleaned$Site)
pheno_DB.training.cleaned$Block = as.factor(pheno_DB.training.cleaned$Block)
pheno_DB.training.cleaned$BG = as.factor(pheno_DB.training.cleaned$BG)
pheno_DB.training.cleaned$Family = as.factor(pheno_DB.training.cleaned$Family)

#order by Ind.
pheno_DB.training.cleaned = pheno_DB.training.cleaned[order(pheno_DB.training.cleaned$Ind
),]

head(pheno_DB.training.cleaned)

##                          Ind Site Block BG Family    Mom    Dad Height_2015
## 1  E952C.01.02071.002.00407.00  ASS     1  5   2071 P77107 P77105         800
## 2  E952C.01.02071.003.00408.00  ASS     1  5   2071 P77107 P77105         870
## 31 E952C.01.02082.001.01701.00  ASS     1  6   2082 P80134 P80122         830
## 32 E952C.01.02082.002.01702.00  ASS     1  6   2082 P80134 P80122         670
## 61 E952C.01.02093.002.00727.00  ASS     1  6   2093 P80121 P80122         750
## 62 E952C.01.02093.004.00729.00  ASS     1  6   2093 P80121 P80122         730
##                         Ind2
## 1  E952C.01.02071.002.00407.00
## 2  E952C.01.02071.003.00408.00
## 31 E952C.01.02082.001.01701.00
## 32 E952C.01.02082.002.01702.00
## 61 E952C.01.02093.002.00727.00
## 62 E952C.01.02093.004.00729.00
```

```
summary(pheno_DB.training.cleaned)

##                              Ind          Site           Block       BG    
##  E952C.01.02071.002.00407.00:   1   ASS:1204   5      :346   1:495  
##  E952C.01.02071.003.00408.00:   1   SCA:1254   6      :318   2:784  
##  E952C.01.02082.001.01701.00:   1              7      :318   5:677  
##  E952C.01.02082.002.01702.00:   1              4      :311   6:502  
##  E952C.01.02093.002.00727.00:   1              3      :289          
##  E952C.01.02093.004.00729.00:   1              2      :276          
##  (Other)                    :2452              (Other):600          
##      Family           Mom                   Dad               Height_2015
##  2071   :  56   Length:2458        Length:2458        Min.   : 323  
##  2527   :  50   Class :character   Class :character   1st Qu.: 700  
##  2450   :  43   Mode  :character   Mode  :character   Median : 770  
##  2446   :  36                                         Mean   : 769  
##  2397   :  34                                         3rd Qu.: 840  
##  2443   :  33                                         Max.   :1130  
##  (Other):2206                                         NA's   :5     
##                              Ind2     
##  E952C.01.02071.002.00407.00:   1  
##  E952C.01.02071.003.00408.00:   1  
##  E952C.01.02082.001.01701.00:   1  
##  E952C.01.02082.002.01702.00:   1  
##  E952C.01.02093.002.00727.00:   1  
##  E952C.01.02093.004.00729.00:   1  
##  (Other)                    :2452
```

ABLUP data preparation

The registered pedigree was corrected using parental assignment softwares prior to this analysis.

Obtaining the inverse of the pedigree-based additive relationship matrix (*A*) using package ASReml

The “A.inv” object can be directly used in ASReml models.

```
###Get pedigree file from phenotype file.
ped.cleaned = pheno_DB.training.cleaned[,c("Ind", "Dad", "Mom")]


#Obtaining the inverse of the A-matrix (package asreml)
library(asreml)
A.inv = ainverse(ped.cleaned) #Returns the inverse (package ASReml).
head(A.inv)

##      Row Column Ainverse
## [1,]   1      1     67.5
## [2,]   2      2     45.0
## [3,]   3      3     59.5
## [4,]   4      1      7.5
## [5,]   4      4     50.5
## [6,]   5      5     36.0
```

Obtaining the inverse of the pedigree-based dominant relationship matrix (*D*)

The “D.inv” object can be directly used in ASReml models.

```
#Add parents to the pedigree.
parents = unique(c(ped.cleaned$Mom, ped.cleaned$Dad)) #Get a vector of parents.
parents = parents[!is.na(parents)] #Remove NAs if any
#Add parents in the top rows of the table.
ped.cleaned.2 = rbind(data.frame(Ind = parents, Mom = NA, Dad = NA), ped.cleaned)
colnames(ped.cleaned.2) = c("ID","Dam","Sire")


#Preparing the pedigree for the makeD function (package nadiv).
library(nadiv)
ped.cleaned.2 = prepPed(ped.cleaned.2)
D.inv = makeD(ped.cleaned.2)$listDinv #Returns the inverse (package nadiv).

## starting to make D....done
## starting to invert D....done

#Set "INVERSE" as an attribute. This is needed for ASReml-R V4.
attr(D.inv, "INVERSE") <- TRUE
head(D.inv)

##   row column Dinverse
## 1   1      1        1
## 2   2      2        1
## 3   3      3        1
## 4   4      4        1
## 5   5      5        1
## 6   6      6        1
```

GBLUP data preparation

Next, we will calculate the genomic equivalent of these additive and dominance relationship matrices.

The genotype file includes 2458 genotyped and phenotyped trees.

Genotypes must be coded as follow:
0 = 0 minor allele (homozygote for the major allele).
1 = 1 minor allele (heterozygote).
2 = 2 minor alleles (homozygote for the minor allele).

The missing genotyped were already imputed using the sotfware LinkImpute.

Import genotype file:

```
#import genotype file.
Marker_data=read.csv("marker_file.csv", header=T, stringsAsFactors = F)
Marker_data = Marker_data[order(Marker_data$Ind),]
row.names(Marker_data) = Marker_data$Ind #Set rownames.
```

```
Marker_data[1:5,1:5]

##                                                      Ind ss538945502 ss538953815
## E952C.01.02071.002.00407.00 E952C.01.02071.002.00407.00           1           1
## E952C.01.02071.003.00408.00 E952C.01.02071.003.00408.00           2           1
## E952C.01.02082.001.01701.00 E952C.01.02082.001.01701.00           1           0
## E952C.01.02082.002.01702.00 E952C.01.02082.002.01702.00           1           0
## E952C.01.02093.002.00727.00 E952C.01.02093.002.00727.00           0           0
##                             ss538945503 ss538945505
## E952C.01.02071.002.00407.00           0           0
## E952C.01.02071.003.00408.00           0           1
## E952C.01.02082.001.01701.00           0           0
## E952C.01.02082.002.01702.00           0           0
## E952C.01.02093.002.00727.00           0           1
```

Verify that all trees in phenotype file are genotyped.

```
sum(pheno_DB.training.cleaned$Ind %in% Marker_data$Ind) == nrow(pheno_DB.training.cleaned
)

## [1] TRUE
```

Calculating the additive genomic relationship matrix (***G**$_a$*) and its inverse

*Obtaining the **G**$_a$ matrix*

```
library(AGHmatrix)
M1<-Marker_data[,-1] #[,-1] to remove the first column of IDs.
#Obtain the matrix Ga (package AGHmatrix).
Ga = Gmatrix(SNPmatrix = as.matrix(M1), method = "VanRaden")

## Initial data:
##  Number of Individuals: 2458
##  Number of Markers: 4092
##
## Missing data check:
##  Total SNPs: 4092
##   0 SNPs dropped due to missing data threshold of 1
##  Total of: 4092  SNPs
## MAF check:
##  No SNPs with MAF below 0
## Monomorphic check:
##  No monomorphic SNPs
## Summary check:
##  Initial:  4092 SNPs
##  Final:  4092  SNPs ( 0  SNPs removed)
##
## Completed! Time = 37.91  seconds
```

*Obtaining the pedigree-based additive relationship matrix **A** for blending*

We obtain the matrix ***A*** (not the inverse) using the package AGHmatrix:

```
ped.cleaned.3 = ped.cleaned.2
#Replace NA values (grand-mothers) by 0.
ped.cleaned.3[is.na(ped.cleaned.3$Dam),"Dam"] = 0
```

```r
#Replace NA values (grand-fathers) by 0.
ped.cleaned.3[is.na(ped.cleaned.3$Sire),"Sire"] = 0
#Obtain the matrix A in a matrix format (package AGHmatrix).
A = Amatrix(data = ped.cleaned.3, dominance = FALSE)
```

```
## Verifying conflicting data...
## Organizing data...
## Your data was chronologically organized with success.
## Processing a large pedigree data... It may take a couple of minutes...
## Constructing matrix A using ploidy = 2
## Completed! Time = 0.023  minutes
```

Subsetting the matrix to only individuals present in the matrix $\boldsymbol{G_a}$ (i.e., excluding the parents).

```r
A = A[as.character(ped.cleaned$Ind), as.character(ped.cleaned$Ind)]
```

Making sure that the matrices are in the same order before blending:

```r
all.equal(row.names(A), row.names(Ga))
```

```
## [1] TRUE
```

```r
all.equal(colnames(A), colnames(Ga))
```

```
## [1] TRUE
```

Blending to make the matrix $\boldsymbol{G_a}$ *invertible and computing the inverse*

```r
Ga_blended = 0.98* Ga + 0.02*A
iGa<-solve(Ga_blended) #Get inverse of matrix Ga_blended by solving.
range(iGa)
```

```
## [1] -4.557693 17.396042
```

The range of values in the inverse should be small numbers.

Getting ready for ASReml-r V4 package

```r
#Transform to sparse matrix (i.e., sorting in a different way, only records were there ar
e no zeros).
iGa<-as(iGa,"sparseMatrix")
#Get lower diagonal of the matrix and get ready for use in ASReml (package MCMCglmm).
library(MCMCglmm)
Ga.inv<-sm2asreml(iGa)
#Set "INVERSE" as an attribute. This is needed for ASReml-R V4.
attr(Ga.inv, "INVERSE") <- TRUE
```

Calculating the dominant genomic relationship matrix (G_d) and its inverse

Obtaining the matrix G_d

```
M1<-Marker_data[,-1] #[,-1] to remove the first column of IDs.
#Obtain the matrix Gd (package AGHmatrix).
Gd = Gmatrix(SNPmatrix = as.matrix(M1), method = "Vitezica")

## Initial data:
##  Number of Individuals: 2458
##  Number of Markers: 4092
##
## Missing data check:
##  Total SNPs: 4092
##   0 SNPs dropped due to missing data threshold of 1
##  Total of: 4092  SNPs
## MAF check:
##  No SNPs with MAF below 0
## Monomorphic check:
##  No monomorphic SNPs
## Summary check:
##  Initial:  4092 SNPs
##  Final:  4092  SNPs ( 0  SNPs removed)
##
## Completed! Time = 33.94  seconds
```

Obtaining the pedigree-based dominant relationship matrix (D) for blending

We obtain the matrix ***D*** (not the inverse) using the package AGHmatrix:

```
D = Amatrix(data = ped.cleaned.3, dominance = TRUE) #Package AGHmatrix.

## Verifying conflicting data...
## Organizing data...
## Your data was chronologically organized with success.
## Processing a large pedigree data... It may take a couple of minutes...
## Constructing matrix A using ploidy = 2
## Constructing dominance relationship matrix
## Completed! Time = 0.9176667  minutes
```

Subsetting the matrix to only individuals present in the G-matrix (i.e., excluding the parents):

```
D = D[as.character(ped.cleaned$Ind), as.character(ped.cleaned$Ind)]
```

Making sure that the matrices are in the same order before blending:

```
all.equal(colnames(Gd), colnames(D))

## [1] TRUE

all.equal(row.names(Gd), row.names(D))

## [1] TRUE
```

Blending to make the matrix $\boldsymbol{G_d}$ invertible and computing the inverse

```
Gd_blended = 0.98* Gd + 0.02*D
iGd<-solve(Gd_blended) #Get inverse of matrix Gd_blended by solving.
range(iGd)

## [1] -2.256419  8.875406
```

The range of values in the inverse should be small numbers.

Getting ready for ASReml-r V4 package

```
#Transform to sparse matrix (i.e., sorting in a different way, only records were there ar
e no zeros).
iGd <-as(iGd,"sparseMatrix")
#Get lower diagonal of the matrix and get ready for use in ASReml (package MCMCglmm).
Gd.inv<-sm2asreml(iGd)
#Set "INVERSE" as an attribute. This is needed for ASReml-R V4.
attr(Gd.inv, "INVERSE") <- TRUE
```

Calculating the status number (Ns)

In the manuscript, we presented the Ns estimated using the corrected pedigree-based additive relationship matrix calculated as: $Ns = 1/(2 * \theta)$, where Θ is the group coancestry (equation [2] in the manuscript). Note that the coanscestry between individuals is half the relationship coefficient (the numbers in the $\boldsymbol{A}$ or $\boldsymbol{G_a}$ matrices). The group coancestry (Θ) is thus calculated as the average of the complete $\boldsymbol{A}$ or $\boldsymbol{G_a}$ matrices (diagonal and off-diagonals), divided by 2.

Using the corrected pedigree-based additive relationship matrix (progeny trees only):

```
1/(2*mean(as.vector(A)/2))

## [1] 53.42699
```

Thus, this calculation is equivalent:

```
1/mean(as.vector(A))

## [1] 53.42699
```

We can try with the additive genomic relationship matrix, but we obtain a very large number:

```
1/mean(as.vector(Ga))

## [1] 5.208763e+16
```

We also obtain a large number with the blended matrix $\boldsymbol{G_a}$:

```
1/mean(as.vector(Ga_blended))

## [1] 2671.349
```

Summary of objects used in modeling

The “A.inv” and “D.inv” objects are the inverse of additive and dominant pedigree-based relationship matrices, respectively.
The “Ga.inv” and “Gd.inv” objects are the inverse of additive and dominant genomic relationship matrices, respectively.

The “pheno_DB.training.cleaned” table contains the phenotypes.

Double check if all factors are correctly specified:

```
str(pheno_DB.training.cleaned)

## 'data.frame':    2458 obs. of  9 variables:
##  $ Ind        : Factor w/ 2458 levels "E952C.01.02071.002.00407.00",..: 1 2 3 4 5 6 7
8 9 10 ...
##  $ Site       : Factor w/ 2 levels "ASS","SCA": 1 1 1 1 1 1 1 1 1 1 ...
##  $ Block      : Factor w/ 10 levels "1","2","3","4",..: 1 1 1 1 1 1 1 1 1 1 ...
##  $ BG         : Factor w/ 4 levels "1","2","5","6": 3 3 4 4 4 4 3 3 3 2 ...
##  $ Family     : Factor w/ 90 levels "2071","2082",..: 1 1 2 2 3 3 4 4 5 6 ...
##  $ Mom        : chr  "P77107" "P77107" "P80134" "P80134" ...
##  $ Dad        : chr  "P77105" "P77105" "P80122" "P80122" ...
##  $ Height_2015: int  800 870 830 670 750 730 660 660 670 710 ...
##  $ Ind2       : Factor w/ 2458 levels "E952C.01.02071.002.00407.00",..: 1 2 3 4 5 6 7
8 9 10 ...
```

Pedigree-based additive-dominance models (ABLUP-AD)

The following model is run using the ASReml-r V4 package (see Eq. [7] in the manuscript). Note that the effect “at(Site):Block:Family” is the random plot effect, within site. Notice that we use a different ID column for the additive (“Ind”) and the dominance effects (“Ind2”) to avoid confusion in the model.

```
start_time <- Sys.time()
model_ABLUP_AD = asreml(fixed = Height_2015~Site,
                        random = ~at(Site):Block + at(Site):Block:Family +
                                 corh(Site):vm(Ind,A.inv) + corh(Site):vm(Ind2,D.inv),
                        residual = ~ dsum(~id(units)|Site),
                        data = pheno_DB.training.cleaned,
                        workspace = "1000mb") #Allow sufficient memory space.

## Multi-section model using the sigma parameterization.
## ASReml 4.1.0 Thu Feb 16 17:18:40 2023
##          LogLik        Sigma2     DF     wall     cpu
##  1     -12210.62          1.0   2451 17:18:41    0.2 (1 restrained)
##  2     -12188.35          1.0   2451 17:18:41    0.1 (1 restrained)
##  3     -12170.34          1.0   2451 17:18:41    0.1 (1 restrained)
##  4     -12163.36          1.0   2451 17:18:41    0.1
##  5     -12161.82          1.0   2451 17:18:41    0.1
##  6     -12161.76          1.0   2451 17:18:41    0.1
##  7     -12161.76          1.0   2451 17:18:42    0.1

end_time <- Sys.time()
end_time - start_time

## Time difference of 1.923848 secs
```

Obtaining variance components

```
summary(model_ABLUP_AD)$varcomp

##                                  component    std.error  z.ratio bound %ch
## at(Site, ASS):Block           2371.7893282 1219.5137183 1.944865     P   0
## at(Site, SCA):Block           1709.3646699  875.9389412 1.951466     P   0
## at(Site, ASS):Block:Family    1428.3038138  293.5040434 4.866385     P   0
## at(Site, SCA):Block:Family    1700.7494881  338.5036891 5.024316     P   0
## Site:vm(Ind, A.inv)!Site!cor     0.6210374    0.1948893 3.186616     U   0
## Site:vm(Ind, A.inv)!Site_ASS  2074.6390085  791.4386747 2.621352     P   0
## Site:vm(Ind, A.inv)!Site_SCA  2372.5739722 1062.3729643 2.233278     P   0
## Site:vm(Ind2, D.inv)!Site!cor    0.9471066    0.2248279 4.212585     U   0
## Site:vm(Ind2, D.inv)!Site_ASS 2004.0249312 1052.2514496 1.904511     P   0
## Site:vm(Ind2, D.inv)!Site_SCA 4380.7529220 1704.6812368 2.569837     P   0
## Site_ASS!R                    2291.9205162  764.2433081 2.998941     P   0
## Site_SCA!R                    1534.1406415 1171.5362169 1.309512     P   0
```

Calculating genetic parameters

Across-site narrow-sense heritability (equation [10] in the manuscript):

```
vpredict(model_ABLUP_AD, h2~( V5*( ((V6+V7)/2)/((V3+V4)/2+(V6+V7)/2+(V9+V10)/2+(V11+V12)/
2) ) ) )

##     Estimate         SE
## h2 0.1552746 0.08028215
```

Across-site dominance ratio (equation [11] in the manuscript):

```
vpredict(model_ABLUP_AD, d2~( V8*( ((V9+V10)/2)/((V3+V4)/2+(V6+V7)/2+(V9+V10)/2+(V11+V12)
/2) ) ) )

##    Estimate       SE
## d2 0.339969 0.126013
```

Across-site broad-sense heritability (equation [12] in the manuscript):

```
vpredict(model_ABLUP_AD, H2~( ( (V5*(V6+V7)/2) + (V8*(V9+V10)/2) )/((V3+V4)/2+(V6+V7)/2+(
V9+V10)/2+(V11+V12)/2) ) )

##     Estimate      SE
## H2 0.4952436 0.10544
```

Compare the results with Table 3 in the manuscript.

Obtaining predicted breeding and genetic values

Each individual tree is predicted on each site. The resulting table contains 5040 rows (2458 progeny trees + 62 parents, predicted on two sites).

Here is a function to remove spaces and parentheses to facilitate treatment:

```
toformat = function(x){
  x = gsub("\\(","",x)
  x = gsub("\\)","",x)
  x = gsub(" ","",x)
  return(x)
}
```

Get BLUPs of random additive effect for each individual tree and site (i.e. breeding values):

```
#This table contains estimates of all random effect coefficients.
BLUP = summary(model_ABLUP_AD, coef=TRUE)$coef.random
row.names(BLUP) = toformat(row.names(BLUP))
#Search and keep only BLUPs of additive effects.
BLUP_Add = BLUP[grepl("vmInd,A.inv",rownames(BLUP)),]
head(BLUP_Add)

##                                solution std.error     z.ratio
## Site_ASS:vmInd,A.inv_P77105 59.612541  30.46233  1.95692652
## Site_ASS:vmInd,A.inv_P80122 11.520481  31.20948  0.36913407
## Site_ASS:vmInd,A.inv_P77109 -1.414007  28.52119 -0.04957741
## Site_ASS:vmInd,A.inv_P80103  3.070170  31.28973  0.09812069
## Site_ASS:vmInd,A.inv_P79106 31.667591  32.41634  0.97690224
## Site_ASS:vmInd,A.inv_P80107  2.141998  30.74229  0.06967595

#Get the names of individuals.
ID = sapply(strsplit(rownames(BLUP_Add),"_"),function(x) x[3])
#Obtain the site. Each individual is predicted on each site.
Site = sapply(strsplit(rownames(BLUP_Add),":"),function(x) x[1])
Site = sub("Site_","",Site)
#Put ID and BLUPs together in a dataframe.
BLUPs = data.frame(ID, Site, BLUP_Add)
colnames(BLUPs) = c("Ind", "Site", "BV", "BV.std.error", "BV.z.ratio")
rm(BLUP_Add, ID, Site) #Save memory space.
head(BLUPs)

##                                Ind Site        BV BV.std.error  BV.z.ratio
## Site_ASS:vmInd,A.inv_P77105 P77105  ASS 59.612541     30.46233  1.95692652
## Site_ASS:vmInd,A.inv_P80122 P80122  ASS 11.520481     31.20948  0.36913407
## Site_ASS:vmInd,A.inv_P77109 P77109  ASS -1.414007     28.52119 -0.04957741
## Site_ASS:vmInd,A.inv_P80103 P80103  ASS  3.070170     31.28973  0.09812069
## Site_ASS:vmInd,A.inv_P79106 P79106  ASS 31.667591     32.41634  0.97690224
## Site_ASS:vmInd,A.inv_P80107 P80107  ASS  2.141998     30.74229  0.06967595
```

The BVs of the parents are on top of the table.

Get BLUPs of dominance effect for each individual tree (i.e., dominance deviations):

```
#Search and keep only BLUPs of dominance effects.
BLUP_Dom = BLUP[grepl("vmInd2,D.inv",rownames(BLUP)),]
#Get the names of individuals.
ID = sapply(strsplit(rownames(BLUP_Dom),"_"),function(x) x[3])
#Obtain the site. Each individual is predicted on each site.
Site = sapply(strsplit(rownames(BLUP_Dom),":"),function(x) x[1])
Site = sub("Site_","",Site)
#Put ID and BLUPs together in a dataframe.
BLUPs_Dom = data.frame(ID, Site, BLUP_Dom)
colnames(BLUPs_Dom) = c("Ind", "Site", "Dom.deviation", "Dom.std.error", "Dom.z.ratio")
#Merge breeding values and dominance deviations.
BLUPs = merge(BLUPs, BLUPs_Dom, by = c("Ind","Site"))
rm(BLUP, BLUP_Dom, BLUPs_Dom, ID, Site) #Save memory space.
```

Calculate total genetic values (breeding values + dominance deviations):

```
BLUPs$GV = BLUPs$BV + BLUPs$Dom.deviation
nrow(BLUPs) #5040 rows. 2458 progreny trees + 62 parents, predicted on two sites.

## [1] 5040

head(BLUPs)

##                            Ind Site        BV BV.std.error BV.z.ratio
## 1 E952C.01.02071.002.00407.00  ASS 60.539844     34.45363 1.75713983
## 2 E952C.01.02071.002.00407.00  SCA 78.048899     40.18957 1.94201886
## 3 E952C.01.02071.003.00408.00  ASS 75.565367     34.45363 2.19324841
## 4 E952C.01.02071.003.00408.00  SCA 88.029725     40.18957 2.19036257
## 5 E952C.01.02082.001.01701.00  ASS 39.721398     34.31500 1.15755189
## 6 E952C.01.02082.001.01701.00  SCA  2.078386     40.10220 0.05182722
##   Dom.deviation Dom.std.error Dom.z.ratio        GV
## 1      33.00004      36.57200   0.9023308  93.53989
## 2      50.11561      54.79781   0.9145551 128.16451
## 3      54.77291      36.57200   1.4976734 130.33828
## 4      80.59871      54.79781   1.4708381 168.62843
## 5      32.46496      36.79415   0.8823403  72.18636
## 6      45.79328      55.16764   0.8300750  47.87166
```

Genomic-based additive-dominance models (GBLUP-AD)

This is the same model as before (Eq. [7] in the manuscript), but using the genomic relationship matrices instead of pedigree-based matrices. The genetic parameters and genetic values can be obtained in the same way as for the ABLUP-AD model. Please see the results in the manuscript.

```
start_time <- Sys.time()
model_GBLUP_AD = asreml(fixed = Height_2015~Site,
                        random = ~at(Site):Block + at(Site):Block:Family +
                        corh(Site):vm(Ind,Ga.inv) + corh(Site):vm(Ind2,Gd.inv),
                        residual = ~ dsum(~id(units)|Site),
                        data = pheno_DB.training.cleaned,
                        workspace = "5000mb") #Allow sufficient memory space.end_time <-
Sys.time()
end_time - start_time

## Time difference of -3.003355 hours
```
